# Internet-Guided Cognitive Behavioral Therapy for Insomnia Among Patients With Traumatic Brain Injury: A Randomized Clinical Trial

**DOI:** 10.1001/jamanetworkopen.2024.20090

**Published:** 2024-07-09

**Authors:** Molly E. Malarkey, Adele J. Fu, Noushin Mannan, Olivia M. Shaw, Thaddeus J. Haight, Martin R. Cota, Nasreen C. Jahed, J. Kent Werner, David L. Brody

**Affiliations:** 1 The Henry M. Jackson Foundation for the Advancement of Military Medicine Inc, Bethesda, Maryland; 2 Military Traumatic Brain Injury Initiative (formerly the Center for Neuroscience and Regenerative Medicine), Bethesda, Maryland; 3 Department of Neurology, Uniformed Services University of the Health Sciences, Bethesda, Maryland

## Abstract

**Importance:**

Many military service members and veterans report insomnia after sustaining traumatic brain injury (TBI). Limitations of first-line treatment, cognitive-behavioral therapy for insomnia (CBT-I), include availability of qualified clinicians, low completion rates, and cost.

**Objective:**

To investigate the feasibility and efficacy of internet-guided CBT-I (eCBT-I) in military service members and veterans with insomnia and a history of TBI.

**Design, Setting, and Participants:**

This randomized clinical trial of fully remote internet-based interventions and evaluations was conducted from September 1, 2020, to June 30, 2021, with 3 months of follow-up. Participants included a volunteer sample of military service members and veterans aged 18 to 64 years with a history of mild TBI/concussion and at least moderately severe insomnia defined as an insomnia severity index (ISI) score of greater than 14 and Pittsburgh Sleep Quality Index of greater than 4. Self-reported race, ethnicity, and educational level were generally representative of the US military. Data were analyzed from October 21, 2021, to April 29, 2024.

**Intervention:**

Internet-based CBT-I delivered over 6 weekly lesson modules with assigned homework activities.

**Main Outcomes and Measures:**

The prespecified primary outcome measure was change in ISI score over time. Prespecified secondary outcome measures included self-reported measures of depression symptoms, posttraumatic stress disorder (PTSD) symptoms, sleep quality, migraine impact, and fatigue.

**Results:**

Of 204 people screened, 125 were randomized 3:1 to eCBT-I vs online sleep education, and 106 completed baseline evaluations (83 men [78.3%]; mean [SD] age, 42 [12] years). Of these, 22 participants (20.8%) were Hispanic or Latino and 78 (73.6%) were White. Fifty participants completed postintervention evaluations, and 41 completed the 3-month follow-up. Baseline mean (SD) ISI scores were 19.7 (4.0) in those randomized to eCBT-I and 18.9 (5.0) in those randomized to sleep education. After intervention, mean (SD) ISI scores were 13.7 (5.6) in those randomized to eCBT-I and 16.6 (5.7) in those randomized to sleep education. The difference in the extent of reduction in ISI scores between groups was 3.5 (95% CI,−6.5 to −0.4 [*P* = .03]; Cohen *d*, −0.32 [95% CI, −0.70 to −0.04]). In the eCBT-I group, the extent of insomnia improvement correlated with the extent of depressive symptom improvement (Spearman ρ = 0.68 [*P* < .001]), PTSD symptoms (ρ = 0.36 [*P* = .04]), sleep quality (ρ = 0.54 [*P* = .001]), and fatigue impact (ρ = −0.58 [*P* < .001]) but not migraine-related disability.

**Conclusions and Relevance:**

The findings of this randomized clinical trial suggest that fully remote eCBT-I was moderately feasible and effective for self-reported insomnia and depression symptoms in military service members and veterans with a history of TBI. There is great potential benefit for eCBT-I due to low availability and cost of qualified CBT-I clinicians, although optimization of completion rates remains a challenge. Future studies may use home-based objective sleep assessments and should increase study retention.

**Trial Registration:**

ClinicalTrials.gov Identifier: NCT04377009

## Key Points

**Question:**

What is the efficacy of fully automated internet-guided cognitive behavioral therapy for insomnia (eCBT-I) in military service members and veterans with traumatic brain injury (TBI) and moderate to severe insomnia?

**Findings:**

In this randomized clinical trial that included 50 military service members and veterans who completed postintervention evaluation, the Insomnia Severity Index score decreased by 6.0 points in those randomized to eCBT-I vs 2.3 points in those randomized to sleep education. The extent of insomnia improvement correlated with depressive symptom improvement in the eCBT-I group.

**Meaning:**

These findings suggest that when successfully completed, eCBT-I can provide clinical benefits in military service members and veterans with TBI and insomnia.

## Teaser

This randomized clinical trial compares internet-based cognitive behavioral therapy for insomnia and an internet-based education control intervention in veterans and military service members with a history of traumatic brain injury and moderate to severe insomnia.

## Introduction

Traumatic brain injury (TBI) has affected more than 468 000 US military service members between 2000 and 2022.^1^ Military service members and civilians with TBI have reported rates of insomnia between 20% and 70%,^2,3,4,5,6,7^ compared with approximately 10% in the general population.^8^ Insomnia is defined as difficulty initiating or maintaining sleep, causing impairment or distress during the daytime. Insomnia may impact mood, energy levels, cognitive ability, social and family relationships, physical health, daily tasks, suicidal ideation, and mortality risk.^9,10,11,12^ Insomnia is one of the most frequently reported reasons for mental health referrals in the military^13^; the frequency of referrals for insomnia within the US military rose by 372% between 2005 and 2014.^14^

Cognitive behavioral therapy for insomnia (CBT-I) is the first-line treatment for insomnia^15,16,17,18^ and typically requires in-person visits with a trained clinician over 6 to 8 weekly 1- to 2-hour sessions.^19^ Cognitive behavioral therapy for insomnia involves focus on healthy sleep-promoting habits and identification of behaviors that may affect optimal sleep. It can improve sleep outcomes, without the adverse effects of common pharmacotherapies for insomnia. Barriers to widespread implementation of CBT-I, however, include a shortage of CBT-I–trained behavioral sleep medicine clinicians, inadequate screening for insomnia in primary care settings, modest levels of referrals for sleep issues from primary care clinicians, and financial constraints.^16^

To address challenges of in-person CBT-I, an internet-based adaptation of CBT-I (eCBT-I) was developed in 2007 called Sleep Healthy Using the Internet (SHUTi). This program includes the intervention components and techniques associated with traditional CBT-I but does not require any direct involvement from a clinician, resulting in improved availability and cost. Studies of eCBT-I have demonstrated similar efficacy and tolerability to conventional CBT-I,^20,21,22^ along with improvements in quality of life, fatigue, and mood relative to control interventions.^23,24,25,26,27^

However, the efficacy of eCBT-I with no direct involvement from a clinician has not been fully established in the setting of TBI or among military service members and veterans. A previous pilot study of online CBT-I in civilians with TBI demonstrated feasibility and potential efficacy.^28^ Two previous studies of in-person CBT-I in military service members indicated reasonable efficacy as well.^29,30^ However, we are not aware of previous randomized clinical studies of eCBT-I with no direct involvement from a clinician in military service members with TBI.

The original program includes vignettes and lessons that are specific to scenarios commonly occurring in civilian life, which can be very different from those experienced by military service members. We developed a customized version for military use. Customization included patient vignettes, testimonials, and other online materials originally designed for non–military-specific audiences modified to be applicable to the military population, while retaining all of the therapeutic content of the original intervention. We hypothesized that use of eCBT-I would improve insomnia in military service members with TBI and that improvements in insomnia would correlate with improvements in other TBI-related domains.

## Methods

The protocol for this randomized clinical trial was approved by the Uniformed Services University Institutional Review Board. All participants provided written informed consent using an online portal. The consent document was reviewed with each participant by telephone, and participants had unlimited time to ask questions and to make a decision. Participants were required to provide informed consent in English; no surrogate consent was allowed. We followed the Consolidated Standards of Reporting Trials (CONSORT) reporting guideline.

### Study Design

We performed an internet-based randomized clinical trial of eCBT-I compared with an internet-based education control intervention in a 3:1 ratio. Randomization was performed using an electronic random number generator through a centralized computer database. Participant information was entered into the centralized database, and a randomization code was provided to the study team in a fully automated fashion. No block randomization was used. Participants randomized to eCBT-I were given access to an internet site that provided 6 weekly scheduled CBT-I lesson modules with assigned homework activities. Control participants were given online access to educational material about insomnia but did not receive CBT-I interventions. Participants could not be formally blinded, but they were not explicitly told which group (eCBT-I or education) was expected a priori to be more effective. The study was entirely remote; there was no in-person contact between the study team and the participants. The participants used the internet sites autonomously with no intervention from the study team. At the end of the study, participants randomized to the education intervention were offered open-label access to the program.

### Study Participants

US military service members and veterans aged 18 to 64 years with a documented or self-reported history of TBI more than 6 months prior to consent and at least moderately severe insomnia were recruited and enrolled in the study. Traumatic brain injury of any severity was permitted as part of the inclusion criteria (though only participants with mild TBI/concussion enrolled), and there was no upper limit to the time between TBI and consent. Participants with multiple TBIs were included if the most recent TBI occurred more than 6 months prior to consent. Moderately severe insomnia was defined as an Insomnia Severity Index (ISI) score of greater than 14^31,32,33^ and a Pittsburgh Sleep Quality Index (PSQI) of greater than 4.^34,35^ Participants were required to confirm reliable access to a telephone and the internet. A stable regimen of medications for sleep for at least 1 month prior to enrollment was permitted, and participants were not required to discontinue medication regimens. Participants were excluded or withdrawn if they had prior exposure to CBT-I, active bipolar disorder or psychosis, a rapidly progressive illness with life expectancy of less than 6 months, moderate to severe substance use disorder with the exception of nicotine, or routinely irregular work schedules or sleep patterns.

Race and ethnicity were self-reported and included the categories Asian, Black or African American, Hispanic or Latino, White, multiracial, and other race or ethnicity (no additional information was available for the latter category). These data were included because it is standard policy for US Department of Defense–supported studies to do so. These data were important to compare the study participants with the broader military service member and veteran population.

### Participant Assessment

**Figure 1.**
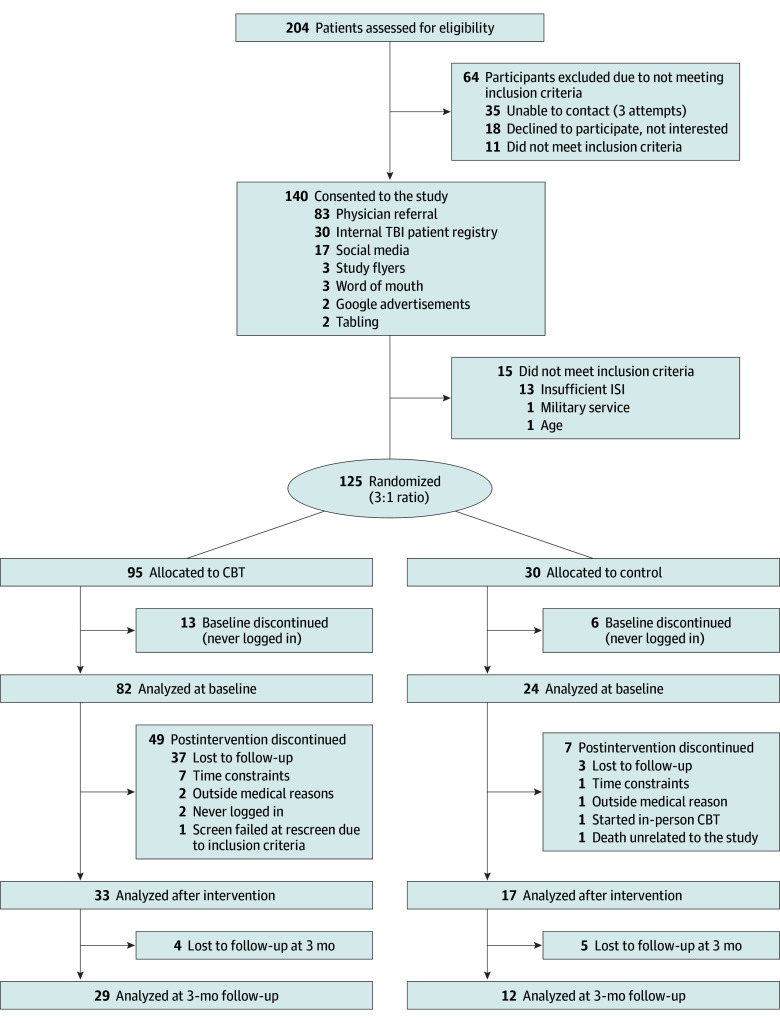
Study Flow Diagram CBT indicates cognitive behavioral therapy; TBI, traumatic brain injury. ISI indicates insomnia severity index.

Participants were assessed at baseline, post intervention at approximately 9 weeks following consent, and at 3-month follow-up. Participants were tracked at screening, enrollment, allocation, and follow-up in accordance with the CONSORT reporting guideline (Figure 1). The trial protocol is provided in Supplement 1. The prespecified primary outcome measure was change in ISI score between baseline and the postintervention assessment in an intention-to-treat analysis. An additional ISI analysis included changes at the 3-month follow-up. Secondary outcomes included changes over time in the following self-report measures: (1) depression symptom severity assessed using the Patient Health Questionnaire 9 (PHQ-9)^36^; (2) posttraumatic stress disorder (PTSD)–related symptoms assessed using the PTSD Checklist for the *Diagnostic and Statistical Manual of Mental Disorders* (Fifth Edition)^37^; (3) sleep quality assessed using the PSQI^35^ with addendum for PTSD^34^; (4) migraine-related disability assessed using the Migraine Disability Assessment (MIDAS; modified to consist of the sum of responses to questions 1, 3, and 5, as questions 2 and 4 are often misinterpreted by participants)^38^; and (5) fatigue-related symptoms assessed using the Functional Assessment of Chronic Illness Therapy–Fatigue.^39^

We also performed as-treated analyses involving only participants who completed all eCBT-I modules and all assessments. No participants allocated to eCBT-I received the control intervention, and no participants allocated to the control intervention received eCBT-I during the randomized phase, though it was made available to them during the crossover phase. In addition, participants were asked about their expectations of benefit prior to starting interventions. After completing interventions, they were asked about whether they thought they were in the active or control group; about the perceived efficacy of the intervention, the perceived benefit of the intervention, and perceived usability of the intervention; and whether they would recommend the intervention to family and friends using Likert scales.

### Statistical Analysis

Data were analyzed from October 21, 2021, to April 29, 2024. Distributions of baseline characteristics were compared between the eCBT-I and control groups with 2-sided *t* tests (continuous measures) and χ^2^ tests (categorical measures). Primary and secondary outcome measures included in the analysis were assessed for normality and outliers. Three participants were determined to be outliers due to their large modified MIDAS scores (>110), which included large changes in MIDAS scores between baseline and the 3-month follow-up. The outliers’ MIDAS scores were removed, and the remaining MIDAS scores were then transformed by using the log-square root function to obtain a normal distribution of the variable. Other outcome measures for these 3 participants were included in analyses. Other outcome measures did not have outliers and were normally distributed. Because of the missing data, data were assessed using a modified intention-to-treat analysis including all available data. For the primary outcome analysis, a linear mixed-effects model was applied to examine the difference in the change in mean ISI between baseline and the postintervention assessment in the eCBT-I group relative to the control group. A 2-sided *t* test and a significance level of *P* < .05 were assumed to assess group difference. Statistical power was assessed at the design phase of the study for a target sample size of 200 and a 3:1 group allocation and reassessed prior to data analysis with the collected study data. Statistical power with the achieved sample size was 0.7 with an assumed significance level of *P* < .05 and a 2-sided test (eMethods in Supplement 2). Separately, change in mean ISI between baseline and the 3-month follow-up between these 2 groups was assessed. As additional secondary outcome analyses, linear mixed-effects models were applied to examine change over time in secondary outcome measures in participants randomized to eCBT-I. Correlations between change in ISI scores between baseline and the postintervention assessment vs change in secondary outcome measures over the same time period were examined with Spearman rank correlations. Analyses were conducted using SAS, version 9.4 (SAS Institute Inc) and R, version 4.2.1 (R Project for Statistical Computing) software packages. Correlations between change in ISI vs change in secondary outcome measures were examined using GraphPad Prism, version 9.5.0 (GraphPad Software Inc). The statistical analysis plan is provided in Supplement 1.

## Results

A total of 204 people were screened and 125 qualifying participants were randomized in a 3:1 ratio of eCBT-I group to control group. Of these, 106 participants completed the baseline evaluations (23 women [21.7%] and 83 men [78.3%]; mean [SD] age, 42 [12] years), 82 of whom were randomized to eCBT-I and 24 of whom were randomized to the control intervention. In terms of race and ethnicity, 5 participants (4.7%) were Asian, 11 (10.4%) were Black or African American, 22 (20.8%) were Hispanic or Latino, 78 (73.6%) were White, 5 (4.7%) were multiracial, and 7 (6.6%) were of other race. A total of 50 participants completed the postintervention assessments, including the primary outcome measure (33 randomized to eCBT-I and 17 randomized to the control group). Of these participants, 39 (78.0%) were men and 11 (22.0%) were women; 1 participant (2.0%) was Asian, 4 (8.0%) were Black or African American, 11 (22.0%) were Hispanic or Latino, 36 (72.0%) were White, 4 (8.0%) were multiracial, and 5 (10.0%) were of other race. Forty-one participants completed the 3-month follow-up assessments, 29 randomized to eCBT-I and 12 randomized to the control group (Figure 1). Study enrollment occurred from September 1, 2020, to June 30, 2021. Enrollment ended before the full prespecified sample size of 200 because the end of the funding period was reached. Recruitment occurred primarily via online advertising, direct referrals from clinicians, flyers, and existing registries. The eCBT-I and control groups were similar in age (mean [SD], 42.6 [10.3] vs 41.8 [12.1] years, respectively), sex (19 of 82 [23.2%] vs 4 of 24 [16.7%] female, respectively), ethnicity (16 of 82 [19.5%] vs 6 of 24 [25.0%], respectively, Hispanic or Latino), educational level (eg, 9 of 24 [37.5%] vs 41 of 82 [50.0%], respectively, with some college or a college degree), military branch (eg, 15 of 24 [62.5%] vs 49 of 82 [59.8%], respectively, in the Army), rank (eg, 8 of 24 [33.3%] vs 33 of 82 [40.2%], respectively, noncommissioned officers), and military occupation type (eg, 16 of 24 [66.7%] vs 57 of 82 [69.5%], respectively, combat) (Table). When asked about race, those randomized to the eCBT-I group included 4 participants (4.9%) who were Asian, 7 (8.5%) who were Black, 65 (79.3%) who were White, 3 (3.7%) who were multiracial, and 3 (3.7%) who were of other race; those randomized to the control group included 1 participant (4.2%) who was Asian, 4 (16.7%) who were Black, 13 (54.2%) who were White, 2 (8.3%) who were multiracial, and 4 (16.7%) who were of other race. At baseline, mean (SD) ISI scores were 19.7 (4.0) in those randomized to eCBT-I and 18.9 (5.0) in those randomized to sleep education, indicating comparable moderate to severe insomnia in both groups. Those who completed the postintervention assessments and those who completed 3-month follow-up assessments also had comparable baseline insomnia severity and demographic characteristics between groups (eTables 1 and 2 in Supplement 2). Comparisons between those who completed the primary outcome and those who were lost to follow-up revealed no major differences in baseline clinical measures or demographics. There was a statistically significant but numerically small difference in PSQI between those who completed the 3-month assessment compared with those with missing assessments. There were statistically significant but numerically modest differences in age and educational level, with completers being older and having higher educational levels than those lost to follow-up (eTables 3-6 in Supplement 2). These differences are not likely to have affected the main results (eMethods in Supplement 2). All participants reported history compatible with mild TBI/concussion based on Department of Defense or Department of Veterans Affairs definitions. No participants with moderate, severe, or penetrating TBI were enrolled.

**Table.** Baseline Participant Characteristics

Characteristics	Study group^a^	
	Control (n = 24)	eCBT-I (n = 82)
Age, mean (SD), y	42 (12)	42 (10)
Sex		
Male	20 (83.3)	63 (76.8)
Female	4 (16.7)	19 (23.2)
Self-reported race		
Asian	1 (4.2)	4 (4.9)
Black or African American	4 (16.7)	7 (8.5)
White	13 (54.2)	65 (79.3)
Multiracial	2 (8.3)	3 (3.7)
Other^b^	4 (16.7)	3 (3.7)
Self-reported ethnicity		
Hispanic or Latino	6 (25.0)	16 (19.5)
Non-Hispanic or Latino	18 (75.0)	66 (80.5)
Self-reported educational level		
High school degree or less	2 (8.3)	11 (13.4)
Some college or college degree	9 (37.5)	41 (50.0)
Graduate degree	13 (54.2)	30 (36.6)
US geographic region		
West	2 (8.3)	4 (4.9)
Midwest	0	1 (1.2)
South	16 (66.7)	53 (64.6)
Northeast	0	2 (2.4)
No response	6 (25.0)	22 (26.8)
Active duty vs retired		
Active duty	19 (79.2)	63 (76.8)
Retired	5 (20.8)	18 (22.0)
No response	0	1 (1.2)
Employment status		
Full-time	17 (70.8)	61 (74.4)
Part-time	0	6 (7.3)
Unemployed	7 (29.2)	14 (17.1)
No response	0	1 (1.2)
Military branch		
Air Force	1 (4.2)	7 (8.5)
Army	15 (62.5)	49 (59.8)
Marine Corps	2 (8.3)	10 (12.2)
Navy	5 (20.8)	16 (19.5)
Other^b^	1 (4.2)	0
Rank		
Junior enlisted	6 (25.0)	11 (13.4)
Noncommissioned officer	8 (33.3)	33 (40.2)
Officer	8 (33.3)	31 (37.8)
Warrant officer	1 (4.2)	2 (2.4)
Other^b^	1 (4.2)	3 (3.7)
No response	0	2 (2.4)
Military occupation type		
Combat	16 (66.7)	57 (69.5)
Noncombat	8 (33.3)	25 (30.5)

^a^ Unless otherwise indicated, data are expressed as No. (%) of patients. Percentages have been rounded and may not total 100.

^b^ No additional information is available regarding the self-reported “other” categories.

**Figure 2.**
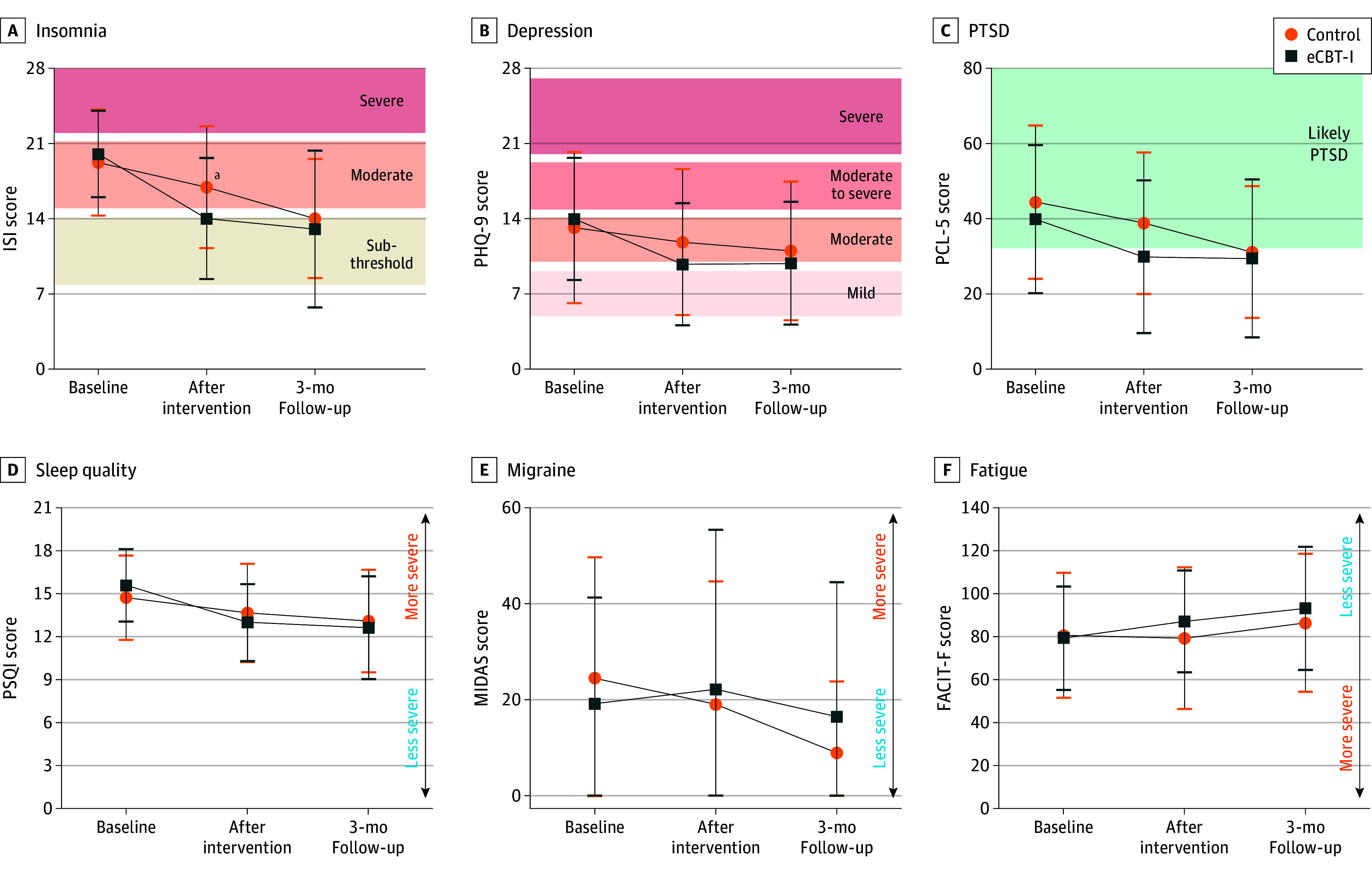
Modified Intention-to-Treat Analyses of Primary and Key Secondary Outcome Measures Including All Available Data A, The Insomnia Severity Index (ISI) scores range from 0 to 28, with scores greater than 14 indicating moderate to severe insomnia. Self-reported insomnia severity constituted the primary outcome measure. B, Depression symptom severity was measured using the Patient Health Questionnaire 9 for depression symptoms. Scores range from 0 to 27, with higher scores indicating more severe depression symptoms. C, Posttraumatic stress disorder (PTSD) symptom severity was measured using the PTSD Checklist for the *Diagnostic and Statistical Manual of Mental Disorders* (Fifth Edition). Scores range from 0 to 80, with higher scores indicating more severe PTSD symptoms. D. Self-reported sleep quality was measured with the Pittsburgh Sleep Quality Index (PSQI). Scores range from 0 to 21, with scores of 4 or greater indicating moderately severe insomnia. E, Migraine-related disability was measured using the Migraine Disability Assessment (MIDAS). Scores range from 0 to 60, with higher scores indicating more severe migraine disability. F, Fatigue impact was measured using the Functional Assessment of Chronic Illness Therapy–Fatigue (FACIT-F). Scores range from 0 to 140, with higher scores indicating less severe fatigue. Data were reported as a function of group (internet-based cognitive behavioral therapy for insomnia [eCBT-I] vs education control) and assessment point. Sample sizes were 82 participants for eCBT-I and 24 for the control groups at baseline, 33 for eCBT-I and 17 for control groups after intervention, and 29 for eCBT-I and 12 for control groups at 3-month follow-up. Error bars indicate SD. ^a^*P* = .03 for difference between groups.

There was a positive effect on the primary outcome measure. Between the baseline and postintervention assessments, improvement in ISI for those randomized to eCBT-I was statistically significant compared with those randomized to sleep education in the modified intention-to-treat analysis (Figure 2A). After intervention, mean (SD) ISI scores were 13.7 (5.6) in those randomized to eCBT-I, a reduction of −6.0. The ISI scores in those randomized to sleep education were 16.6 (5.7), a reduction of −2.3. The difference in the extent of reduction in ISI scores between groups was −3.5 (95% CI, −6.5 to −0.4; Cohen *d*, −0.32 [95% CI, −0.70 to −0.04]; *P* = .03). Three months after intervention, mean (SD) ISI scores were 12.7 (7.3) in those randomized to eCBT-I, a reduction of 7.4 from baseline and a reduction of 1.4 vs immediately after the intervention. Mean (SD) ISI score in the sleep education group was 13.7 (5.6), a reduction of 5.2 from baseline and a reduction of 2.3 vs immediately after the intervention. The difference between groups in the extent of reduction in ISI from baseline to 3 months post intervention was 2.2 (95% CI, −5.6 to 1.2; Cohen *d*, −0.20 [95% CI, −0.59 to 0.13]; *P* = .20). These results suggest that eCBT-I was more effective than sleep education in initially reducing self-reported insomnia, though the groups converged at 3 months.

Expected benefit prior to starting the intervention did not appear to influence the results (eFigure 1 in Supplement 2). Most participants had moderate to high expectation of benefit, and there were no differences in change in ISI between those with moderate vs high expectation of benefit.

Participants in the eCBT-I group who believed they had received the active intervention reported numerically greater decreases in insomnia than those who were uncertain or believed they had received the control intervention (eFigure 2 in Supplement 2). Most participants who received eCBT-I reported high efficacy, perceived benefit, and usability and would likely recommend the intervention to friends and family. Furthermore, high perceived efficacy, perceived benefit, usability, and likelihood of recommending the intervention to family and friends were all associated with numerically greater decreases in insomnia in those who received eCBT-I (eFigure 3 in Supplement 2). Participants in the control group reported less favorable ratings, and there were no associations between ratings and changes in insomnia severity. There was no statistically significant interaction between sleep medication use and group assignment in terms of change in ISI (eFigure 4 in Supplement 2).

Improvements occurred over time in secondary outcome measures, most notably in depression symptoms. However, there were no statistically significant differences between groups for the secondary outcome measures. Between the baseline and postintervention assessments, there was a statistically significant improvement in self-reported depression (PHQ-9 score) in those randomized to eCBT-I (Figure 2B). The reduction in PHQ-9 score from baseline to postintervention assessments was 3.6 (95% CI, −5.0 to −2.1 [*P* < .001]; Cohen *d*, −0.84 [95% CI, −1.53 to −0.60]). The reduction in PHQ-9 score from baseline to 3 months post intervention was 3.9 (95% CI, −5.4 to −2.3 [*P* = .002]; Cohen *d*, −0.92 [95% CI, −1.38 to −0.48]). Similar improvements were noted in measures of PTSD (Figure 2C) post intervention. There were no differences between groups in sleep quality (Figure 2D), migraine-related disability (Figure 2E) or fatigue (Figure 2F). The as-treated analyses revealed similar overall findings (eFigure 5 in Supplement 2), though effects on depression were attenuated. These associations appeared to be continuous; we did not observe evidence for dichotomous responders vs nonresponders in any of these domains.

**Figure 3.**
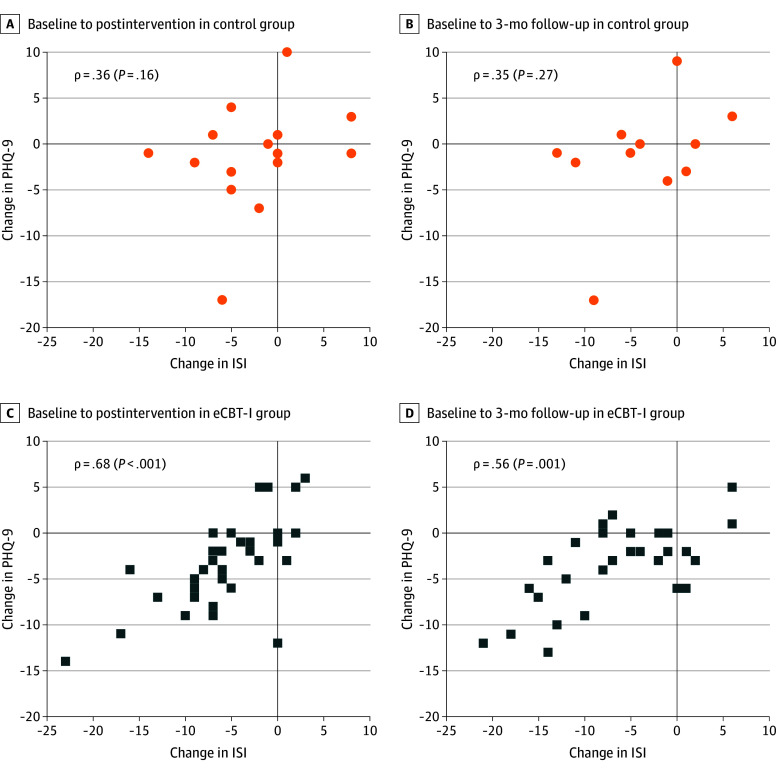
Correlation Between Changes in Self-Reported Insomnia and Changes in Depression Symptom Severity Correlations for the group with internet-based cognitive behavioral therapy for insomnia (eCBT-I) (blue squares) are plotted separately from correlations for control group (orange circles). Spearman nonparametric correlation coefficients are reported. ISI indicates Insomnia Severity Index; PHQ-9, Patient Health Questionnaire 9.

The extent of improvement in insomnia correlated with the extent of improvement in depression severity in those randomized to eCBT-I (Figure 3). The correlation was strongest for the postintervention assessments (Spearman ρ = 0.68 [*P* < .001]) with somewhat attenuated correlation for the 3-month follow-up assessments (Spearman ρ = 0.56 [*P* = .001]). There was no correlation in those randomized to the education control intervention. In the eCBT-I group, there were also correlations between the extent of improvement in insomnia and the improvement in PTSD symptoms (ρ = 0.36 [*P* = .04] after intervention; ρ = 0.42 [*P* = .02] at follow-up) (eFigure 6 in Supplement 2), sleep quality (ρ = 0.54 [*P* = .001] after intervention; ρ = 0.69 [*P* < .001] at follow-up) (eFigure 7 in Supplement 2), and fatigue impact (ρ = −0.58 [*P* < .001] after intervention; ρ = −0.40 [*P* = .04] at follow-up) (eFigure 8 in Supplement 2) but not migraine-related disability (eFigure 9 in Supplement 2). The correlations in those randomized to the education control intervention were markedly attenuated.

There were no important harms attributed to study participation. No unintended effects were reported by any of the participants.

## Discussion

This randomized clinical study demonstrated that fully remote eCBT-I treatment without clinician intervention was moderately feasible and effective for self-reported insomnia in military service members and veterans with a history of mild TBI/concussion. Furthermore, greater improvements in insomnia were quantitatively correlated with greater improvement in depression, PTSD, sleep quality, and fatigue. While the study could not be fully blinded, the preintervention expectation of benefit did not appear to bias the results.

The implications for clinical care of fully remote eCBT-I treatment without required clinician intervention are substantial. At present, CBT-I and other evidence-based psychotherapies delivered by trained clinicians are limited by clinician availability, participant schedules, cost, and stigma associated with therapy. Internet-based CBT-I provides the availability to anyone with access to the internet, flexible scheduling, low cost per participant once the digital therapeutic intervention has been developed, and improved privacy, which may reduce stigma. Nonetheless, barriers to eCBT-I and other digital therapeutics include incomplete understanding of efficacy, relatively low awareness, and unknown community-wide acceptance.

### Strengths and Limitations

Strengths of this study include the randomized design of an intervention that addresses an unmet need and the fully remote operation of the trial. Most participants who received eCBT-I reported high efficacy, perceived benefit, and usability, and stated that they would likely recommend the intervention to friends and family.

This study also has some limitations. The rate of loss to follow-up was high, which caused the sample size and statistical power to be lower than anticipated. Loss to follow-up may have been higher than in previous trials of fully remote digital therapeutics (mean, 25% [range, 0-43%]).^21^ It is possible that eCBT-I may be more challenging in this population than in other populations. Loss to follow-up is also a challenge for in-person psychotherapeutic interventions.^40,41^ Second, we did not objectively assess sleep physiology or potential insomnia confounds such as obstructive sleep apnea. Polysomnography would have been impractical for a 100% remote study design. There are many innovations in home-based sleep assessment tools, but at the time this study was designed and funded (2018-2019), these tools had not been sufficiently validated to justify their use. Third, we have not directly compared eCBT-I with in-person clinician-delivered CBT-I in this population or other digital therapeutics; it may be most appropriate to consider them to be different interventions. Fourth, we have not assessed effects in other populations with TBI such as younger, older, or civilian patients and those with more severe TBI. Our study enrolled substantially more men than women, as would be expected for this population; the study was not sufficiently powered to address differences in efficacy as a function of smaller subgroups. Additional limitations are discussed in the eAppendix in Supplement 2.

## Conclusions

The domain of fully remote digital therapeutics has tremendous potential to benefit many types of patients with neurological and psychiatric conditions. This study adds to the evidence base by demonstrating that a remote cognitive behavioral therapy for insomnia intervention improves insomnia, depression, and PTSD symptoms in military service members and veterans with a history of TBI. Future insomnia studies may use home-based objective sleep assessment tools, several of which are in the process of being validated.^42,43^ Improving trial retention is a major goal for clinical trial teams in this domain. Anecdotally, completion of the sleep diaries was reported by several participants to be the most onerous part of the study, and alternatives to daily diaries will be explored. Likewise, digital therapeutics have not yet been integrated into the standard practice of the military heath system and Veterans Affairs medical system.

## Notes

Supplement 1.
Trial Protocol and Statistical Analysis Plan

Supplement 2. **eFigure 1.** Expected Benefit Prior to Starting the Intervention vs Change in ISI From Baseline to Post Intervention **eFigure 2.** Believed Group Assignment vs Change in ISI From Baseline to Post Intervention **eFigure 3.** Participant Ratings of the Intervention vs Change in ISI From Baseline to Post Intervention **eFigure 4.** Interaction Between Sleep Medication Use and Group Assignment in Effects on ISI **eFigure 5.** As-Treated Analyses of Primary and Key Secondary Outcome Measures Involving Only Participants Who Completed All Online Modules and All Assessments **eFigure 6.** Correlations Between Changes in Self-Reported Insomnia and Changes in PTSD Symptom Severity, With Spearman ρ Values **eFigure 7.** Correlations Between Changes in Self-Reported Insomnia and Changes in Self-Reported Sleep Quality, With Spearman ρ Values **eFigure 8.** Correlations Between Changes in Self-Reported Insomnia and Changes in Self-Reported Fatigue Impact, With Spearman ρ Values **eFigure 9.** Correlations Between Changes in Self-Reported Insomnia and Changes in Migraine-Related Disability, With Spearman ρ Values **eTable 1.** Demographic Characteristics of Participants Who Completed Immediate Post Intervention Follow-Up Evaluations **eTable 2.** Demographic Characteristics of Participants Who Completed 3-Month Follow-Up Evaluations **eTable 3.** Baseline Clinical Scores for Participants Who Completed vs Were Missing Immediate Postintervention Follow-Up Evaluations **eTable 4.** Baseline Clinical Scores for Participants Who Completed vs Were Missing 3-Month Post-Intervention Follow-Up Evaluation **eTable 5.** Demographics for Participants Who Completed vs Were Missing Immediate Post-Intervention Follow-Up Evaluations **eTable 6.** Demographics for Participants Who Completed vs Were Missing at 3-Month Post Intervention Follow-Up Evaluations **eMethods.** Statistical Analysis **eAppendix.** Additional Limitations
eReferences

Supplement 3.
Data Sharing Statement
