# Supplementary material for: Internet-Guided Cognitive Behavioral Therapy for Insomnia Among Patients With Traumatic Brain Injury: A Randomized Clinical Trial
Source: JAMA Netw Open. 2024 Jul 9;7(7):e2420090. doi: 10.1001/jamanetworkopen.2024.20090 (PMC11234237; doi:10.1001/jamanetworkopen.2024.20090)
Supplement: Supplement 1. — Trial Protocol and Statistical Analysis Plan [file jamanetwopen-e2420090-s001.pdf]

UNIFORMED UNIVERSITY OF THE HEALTH SCIENCES AT BETHESDA, MD

GENERAL INFORMATION

Protocol Title:
A Randomized, Controlled, Double-blinded Study of Internet-guided Cognitive Behavioral Therapy for Insomnia in Military Service Members with History of Traumatic Brain Injury

Principal Investigator:
David L. Brody, MD, PhD
Professor, Department of Neurology
Uniformed Services University
Director, Center for Neuroscience and Regenerative Medicine
314-537-6453
david.brody@usuhs.edu

Associate Investigator(s):
Martin Cota
Senior Clinical Trials Specialist
Henry M. Jackson Foundation
Center for Neuroscience and Regenerative Medicine
Uniformed Services University
301-295-9685
martin.cota.ctr@usuhs.edu

Alura Johnston
Senior Regulatory Affairs Specialist
Henry M. Jackson Foundation
Center for Neuroscience and Regenerative Medicine
Uniformed Services University
301-295-9207
alura.johnston.ctr@usuhs.edu

John Kent Werner, MD, PhD
Staff Neurologist / Sleep Medicine Fellow
Walter Reed National Military Medical Center
301-346-2696
john.k.werner.mil@mail.mil

Thaddeus Haight, PhD
Biostatistician
Henry M. Jackson Foundation
Center for Neuroscience and Regenerative Medicine
Uniformed Services University
301-295-9700
thaddeus.haight.ctr@usuhs.edu

Bahareh Kost
Data Manager
Henry M. Jackson Foundation
Center for Neuroscience and Regenerative Medicine

Uniformed Services University
301-295-6455
Bahareh.kost.ctr@usuhs.edu

Collaborator(s):
Lee M. Ritterband, PhD
Professor, Psychiatry and Neurobehavioral Sciences
University of Virginia School of Medicine
434-924-5988
Lr5b@hscmail.mcc.virginia.edu

STUDY DETAILS

Abstract/Summary:

Purpose

To investigate the feasibility and efficacy of Internet-guided Cognitive Behavioral Therapy for Insomnia (eCBT-I) in active and retired military service members with insomnia and a history of traumatic brain injury (TBI).

Subject Population

Male and female active or retired service members, eligible for care at Department of Defense (DoD) facilities, between the ages of 18 and 64 with insomnia and a history of TBI.

Research Design

Internet-based, double-blinded, controlled, prospective, randomized interventional trial with an optional subsequent open-label intervention. Up to 200 participants will be randomized to either active eCBT-I or education control groups in a 3:1 ratio, respectively.

Methodology /Technical Approach

This study seeks to validate an eCBT-I program as an alternative to traditional in-person therapy for participants with insomnia. Up to two hundred (n=200) active or retired service members with history of TBI will be enrolled over a period of two years. Participants will be randomized to active intervention or education control groups in a 3:1 ratio, respectively. Participants receiving active eCBT-I will receive online portal access and follow a 9-week eCBT-I intervention program requiring completion of regular follow-up and out
come assessments via telephone and the Internet. Participants randomized to the control group will receive insomnia education and monitoring only through a mimetic online education control portal and will complete a follow-up and outcome assessment schedule identical to the active intervention group. Content available to control participants through the education control portal is not individually tailored and does not provide an online means for ongoing insomnia symptoms assessment or allow for documentation of sleep diaries. Participant experience and interaction with the study team will be entirely electronic or by telephone so as to increase the likelihood of study procedure compliance, potential for immediate benefit, enrollment, and generalizability to possible policy changes within military health system (MHS) favoring eCBT-I as first line intervention for insomnia.

The primary outcome measure of efficacy will be the Insomnia Severity Index (ISI). Primary analysis of the primary outcome will be percent improvement in ISI scores assessed pre- and post-intervention in an “intention to treat” fashion. Secondary analyses of the primary outcome will include “as treated” analyses and retained improvement in ISI scores assessed between pre-intervention baseline and long-term follow up at 3 months. Secondary outcome measures will capture known and suspected insomnia precursors, predictors, or correlates including Posttraumatic Stress Disorder Checklist for DSM-5 criteria (PCL-5),

Patient Health Questionnaire 9 for Depression (PHQ-9) with question #3 regarding sleep redacted for redundancy, and Migraine Disability Assessment (MIDAS). Given that many service members with TBI also have PTSD, we will use the Pittsburgh Sleep Quality Index (PSQI) with Addendum for PTSD (PSQI-A) as secondary measures to assess insomnia. Additional secondary outcomes will assess quality of life through the Functional Assessment of Chronic Illness Therapy for Fatigue (FACIT-F), qualitative feedback from participants including assessment of blinding efficacy, and study team blinding efficacy. We will assess attitudes towards telemedicine versus in-person therapies of this type. As part of this investigation, it is expected that participants will not only significantly benefit from the active intervention as compared to the control but also increase the likeliness of help-seeking and compliance behavior given the flexibility, broad availability and immediately impactful benefit of this internet-based approach.
Study team members interacting with participants will be blinded to group assignment until completion of long-term follow up, at which point participants randomized to the education control group will be given the option to receive open-label eCBT-I intervention for an equivalent period of 9 weeks.

Key Words:
Cognitive Behavioral Therapy, Insomnia, Concussion, Mild Traumatic Brain Injury

Background and Significance:

Traumatic Brain Injury (TBI) has emerged as one of the most frequently diagnosed medical conditions, affecting upwards of 371,000 service members between 2000 and 2017 (Defense and Veterans Brain Injury Center, 2017). TBI has profound clinical and resource implications for the Department of Defense (Swanson et al., 2017). Despite being the most common neurological injury sustained by service members, it is hypothesized that TBI frequently remains undiagnosed and untreated (Defense and Veterans Brain Injury Center, 2017). A 2007 report from the President's Commission on Care for America's Returning Wounded indicated that 10-20% of apparently healthy service members returning from conflicts in Iraq and Afghanistan met diagnostic criteria for concussive TBI, also known as mild TBI (President's Commission on Care for America's Returning Wounded Warriors, 2007). A high percentage of these service members with suspected or confirmed TBI are also diagnosed with insomnia and other sleep disorders.

Insomnia sleep disorder is broadly defined as the presence of one or more of the following symptoms without clear secondary etiological or biological cause: difficulty initiating sleep, difficulty maintaining sleep, waking up too early, or experiencing nonrestorative or low quality sleep (Roth, 2007; Ancoli-Israel and Roth, 1999). Recent findings suggest a growing number of active and retired service members suffer from symptoms of insomnia. In a 2013 study of 110 military personnel returning from combat within 18 months of deployment, 63.6% of participants met diagnostic criteria for insomnia, while 62.7% met diagnostic criteria for obstructive sleep apnea (OSA) (Mysliwiec et al., 2013). Other studies in both military and civilian populations support a growing body of evidence demonstrating persons sustaining a TBI are more likely to experience insomnia with greater frequency and severity (Bryan, 2013; Hou et al., 2013; Viola-Saltzman & Watson, 2012; Ponsford et. al, 2012). These collective findings suggest a markedly higher risk of insomnia among active and retired service members when compared with the general population, which has an observed incidence of approximately 10% (Singareddy et al., 2012).

Although etiology is unclear, many service members did not have insomnia prior to brain injury, or their symptoms substantially worsened after initial and repeated injury. Studies investigating an active duty military cohort have reported incidence of insomnia was 20.4% following single TBI and 50.0% for those with multiple TBIs (Bryan, 2013). The incidence of insomnia following TBI varies significantly, with estimates ranging from 30% to as high as 70% (Viola-Saltzman & Watson, 2012; Ponsford et al.,

2012). Our current understanding is further complicated by high rates of comorbid disorders, including depression and posttraumatic stress disorder (PTSD). Not surprisingly, with a large number of TBIs and its high comorbidity among service members, insomnia is one of the most frequently reported reasons for mental health referrals in the military (Cozza et al., 2004). While the severity of insomnia is generally positively correlated with injury severity, the greatest number of persons reporting post-injury sleep complaints are those with mild TBI (mTBI), which accounts for approximately 83% of reported TBIs (Department of Defense, 2017). Disconcertingly, recent studies have also shown the frequency of referrals for insomnia within the US military have risen by as much as 372% between the years 2005 and 2014, indicating a growing awareness and concern for providers that necessitates aggressive prioritization and intervention (Caldwell et al., 2017).

Cognitive Behavioral Therapy for Insomnia (CBT-I)

The standard intervention approach for CBT-I consists of cognitive-behavioral strategies intended to improve outcomes in overall sleep quality, staying asleep, and falling sleep. Intervention strategies focus on sleep-promoting habits and identification of behaviors that may affect a person's ability to maintain optimal sleep tendencies. For a period of approximately 1-2 weeks, patients are instructed to maintain sleep diaries that document general information about their sleep habits such as time to rise, time to bed, number of awakenings, sleep onset latency, time of final awakening, overall sleep quality, frequency of naps, and similar related questions. The purpose of the initial sleep assessment and observation period is to establish a baseline for subsequent intervention techniques. In-person visits with the provider are conducted weekly for 6 or more sessions lasting approximately 2 hours per session (Morin et al., 2006). Subsequent study visits teach patients specific techniques in several domains.

1. Stimulus Control advises patients on procedural and lifestyle changes to remove or limit exposure to sleep-curtailing behaviors. Practitioners will recommend to patients that they avoid partaking in known stimulatory activities several hours before and during the process of going to bed, such as avoiding vigorous exercise, caffeine, responding to work emails, and related activities. Additional practitioner recommendations are designed to reassociate the sleep environment with sleep and encourage the implementation of healthy routines. These recommendations include only using the bed for sleep and sexual activity, only going to bed when feeling tired, getting out of bed when an individual is unable to sleep, establishing a consistent daily waking time, and avoiding napping. Controlled studies investigating the therapeutic potential of stimulus control therapy have suggested (Epstein et al., 2012; Morin et al., 1994; Riedal et al., 1998; Turner & Ascher, 1979).
2. Sleep Restriction imposes limitations on patients as to the total duration and times of day they may spend asleep or in bed. Recommended sleep restriction guidelines are developed using baseline sleep diaries and limit patients to only using their bed during the predicted period of actual sleep. For example, if a patient reports spending 9 hours in bed daily while only sleeping 5 hours, the patient would be advised to spend only 5 hours in bed per day during specific times. Adjustments to sleep restriction guidelines are made throughout the therapy course gradually increasing the allowable time in bed as the patient's sleep efficiency (the time spend asleep / the time spend in bed) improves. Sleep restriction is hypothesized to be one of the most influential determinants of therapeutic success when utilized independently or as part of a multi-component CBT-I approach (Epstein et al., 2012; Morin et al., 1994).
3. Relaxation therapies are taught to help alleviate cognitive and psychosomatic arousal before bed (Morin et al., 1994; Turner & Ascher, 1979). Examples of relaxation techniques typically employed are intended include meditation, thought stopping, imagery training, progressive muscle relaxation, and biofeedback.
4. Cognitive Therapy, also known as paradoxical intention, is a technique intended to treat performance anxiety patients may have regarding sleep. A practitioner will advise the patient to

stop making an active effort to sleep under the premise that sleep-related performance anxiety inhibits successful and natural sleep onset (Morin et al., 1994; Turner & Ascher, 1979).

5. Sleep Hygiene employs various educational components that teach patients about health practices such as poor diet, limited exercise, substance abuse, other medical conditions, as well as light and noise exposure and how they may contribute to insomnia (Morin et al., 1994). Although the therapeutic efficacy of sleep hygiene regimens have not been conclusively demonstrated in controlled studies, practitioner recommendations are believed to be an important factor of a multi-component CBT-I intervention (Stepanski & Wyatt, 2003; Morin et al., 1994)Sleep hygiene employs various educational components that teach patients about health practices such as exercise, diet, substance abuse, other medical conditions, as well as light and noise exposure and how they may contribute to insomnia.

CBT-I has been extensively validated for efficacy in a general population for lessoning of insomnia symptoms. A recent meta-analysis of randomized controlled studies of CBT-I conducted between 1990 and 2009 confirmed efficacy according to intra-group CBT-I and CBT-I versus control group comparisons (Okajima et al., 2010). Intra-group comparison of CBT-I efficacy revealed medium to large effect sizes of intervention on subjective sleep variables at the post-intervention endpoint that were largely maintained at long-term follow up. Between-group comparisons revealed CBT-I was more effective than the control condition for improving subjective sleep variables when measured post-intervention and long-term follow-up. These findings are in agreement with previous literature that shows CBTI-I as effective when compared to placebo intervention with effect sizes of 0.88 for sleep latency (time spent before successfully falling asleep) and 0.65 for time awake after sleep onset (total duration of awakenings after initially falling sleep) (Morin et al., 1994; Morin et al., 2006; Buscemi et al., 2005). Other studies have since confirmed the efficacy of CBT-I as a standalone intervention when compared to other therapies for insomnia (Davidson et al., 2017; Wu et al., 2015). When compared to drug interventions, there is also evidence to suggest CBT-I may have equal or greater impact on improved sleep outcomes than certain pharmaceutical interventions (Mitchell et al., 2012; Jacobs et al., 2004). Following successful implementation of insomnia intervention, studies have demonstrated potential health and quality of life improvements in secondary but related domains such as decreased depression (Taylor et al., 2007) and PTSD (Nappi et al., 2012; Margolies et al., 2013).

The primary limitations of CBT-I and challenge for care-seeking patients are a relative paucity of qualified providers (Davy et al., 2013) and lack of patient reimbursement for online therapy received outside of Military Healthcare System (MHS). In addition to a shortage of available providers, those able to provide assistance may be inaccessible or impractical for many patients due to geographic and time availability considerations. Not surprisingly, patient compliance and feasibility of intervention delivery are hypothesized to play a significant role in overall efficacy rates, although inconsistent sleep outcomes and intervention adherence reporting complicate scientific interpretation (Matthews et al., 2013).

The inherent limitations of traditional CBT-I may be particularly evident within the MHS (Ulmer et al., 2017), where patient availability and frequent relocation of military service members or providers pose additional challenges in for the management of insomnia. In order to help close the patient-practitioner gap and provide better care, recent trials have utilized actigraphic wristwatches. These actigraphy wristwatches serve as wearable biofeedback devices capable of capturing traditionally self-reported outcomes such as sleep onset latency, sleep duration, sleep efficiency, and circadian rhythm data (Devine et al., 2017; Kravitz et al., 2015). Compared to polysomnography, electroencephalography, and other traditional “gold standard” biofeedback technology, the use of actigraphy in sleep medicine has demonstrated reasonable scientific validity and usability (Sadeh, 2011; Morganthaler et al., 2007). However, while these newer technologies may provide greater sensitivity in conditions such as circadian rhythm or sleep-schedule disorders when the comparative outcome is polysomnography (Ancoli-Israel et al., 2003; Sack et al., 2007), recent studies have demonstrated that objective outcomes and self-report

measures of sleep quality are only moderately correlated and therefore likely associated with distinct psychological and biological processes. For instance, Jackowska and colleagues (2016) found that self-report measures of sleep quality such as the PSQI were predictive of overall psychological wellbeing, whereas objective measures of sleep duration and efficiency were unrelated to psychological outcomes. Incidentally, Jackowska et al. also found that sleep duration derived from diaries was highly correlated with objective duration, yet neither were correlated with psychological wellbeing. In studies specifically investigating insomnia, actigraphy measures have consistently overestimated sleep time due to users lying motionless for long periods while attempting to fall asleep (Sadeh, 2011; Hauri and Wisbey, 1992). In addition, actigraphy watches impose significant cost and logistical burden with still-limited reliability relative to gold standard sleep medicine technologies found in fully-equipped laboratories (Blackwell et al., 2008; Marino et al., 2013). Self-reported sleep outcomes have been corroborated for many years as scientifically valid and pragmatic in the context of numerous insomnia and other sleep-related investigations (Gagnon et al., 2013; Bastien et al., 2001; Jenkins et al., 2015; Morin et al., 2011; Carpenter and Andrykowski, 1998; Buysse et al., 1989). Collectively, these studies suggest that self-report measures of sleep quality such as the PSQI are ideal for determining the effects of disrupted sleep on overall psychological health and wellbeing. Furthermore, self-report measures such as the ISI and PSQI have been shown to be particularly sensitive to detecting changes in sleep quality outcomes most relevant to studying CBT-I (Geiger-Brown et al., 2015).

Sleep Health Using the Internet (SHUTi)

In an effort to address these challenges and provide a robust national care network, an Internet-guided adaptation of CBT-I (eCBT-I) has recently been developed as a viable alternative to conventional in-person therapy. Sleep Healthy Using the Internet (SHUTi) was created in 2007 by the University of Virginia (UVA, 1215 Lee St, Charlottesville, VA 22908) to be an interactive online alternative to in-person CBT-I for treating adult insomnia sleep disorder. SHUTi is designed to include all intervention components, techniques, and corresponding benefits associated with traditional CBT-I without potential availability, financial, or practical limitations that face many patients seeking conventional forms of assistance.

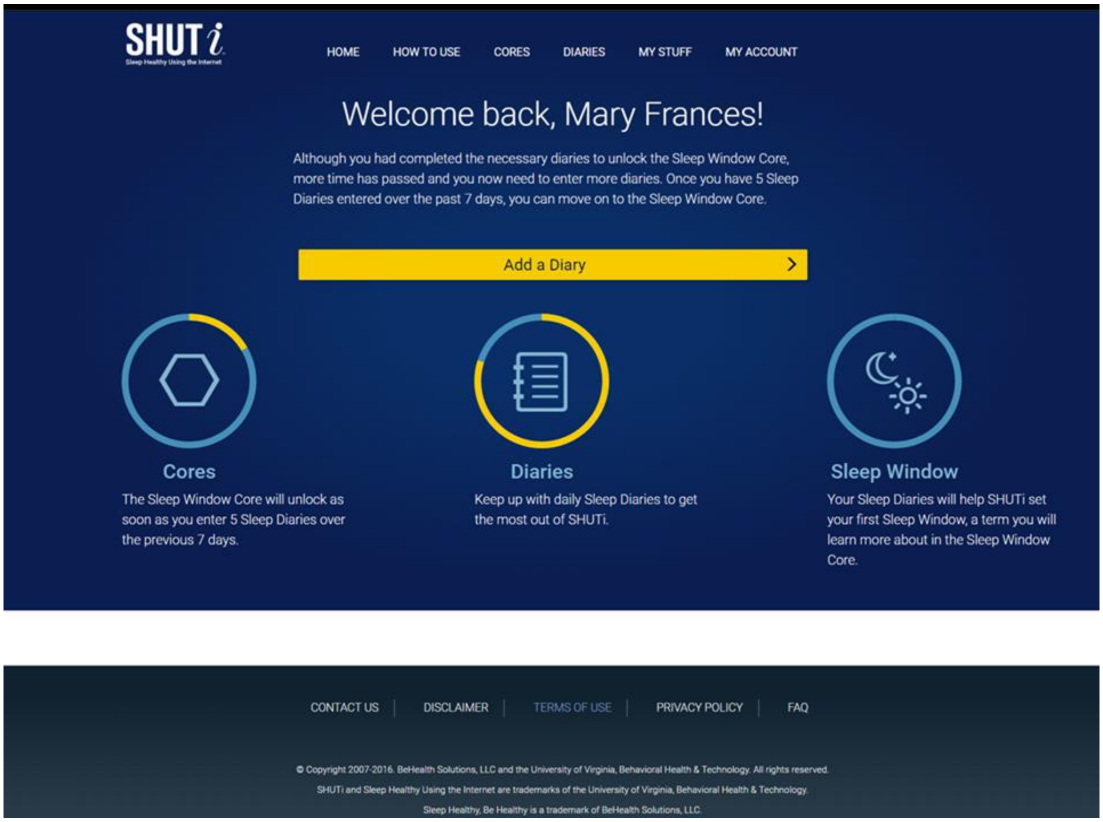

Figure 1. Example welcome page for SHUTi's online intervention portal. Patients going through intervention have access to customized insomnia educational modules, sleep diary logs, and learning comprehension assessments.

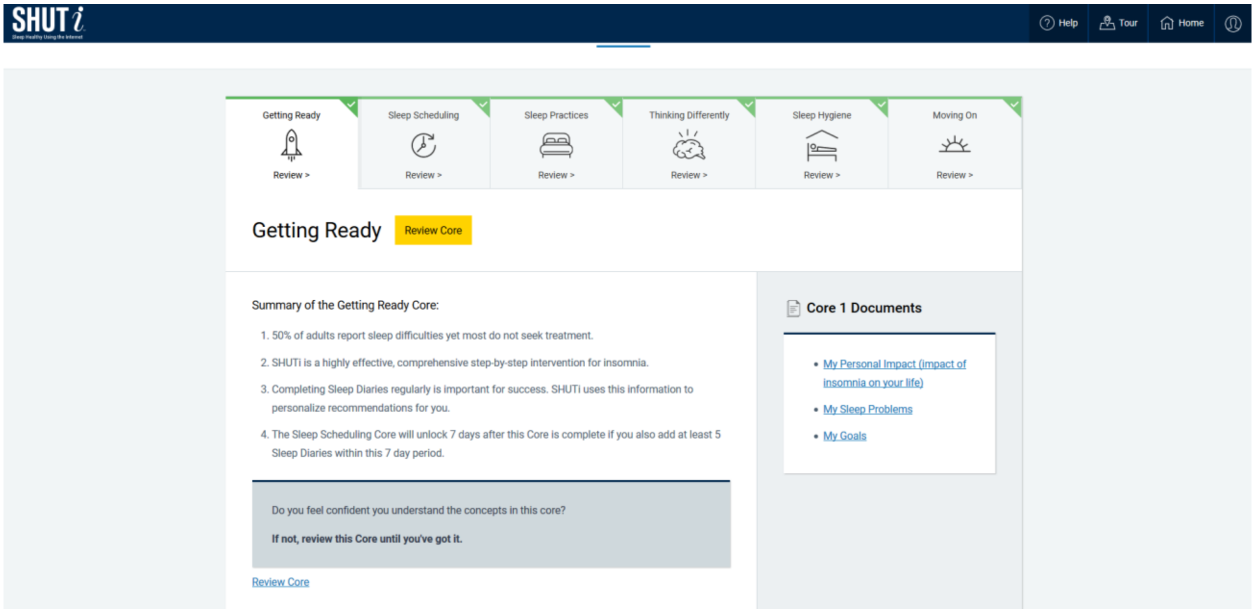

Figure 2. Example eCBT-I training module, or SHUTi "sleep core", in which patients are introduced to key behavioral intervention techniques to help decrease their insomnia symptoms. The online portal contains 6 training modules following a week long baseline assessment period.

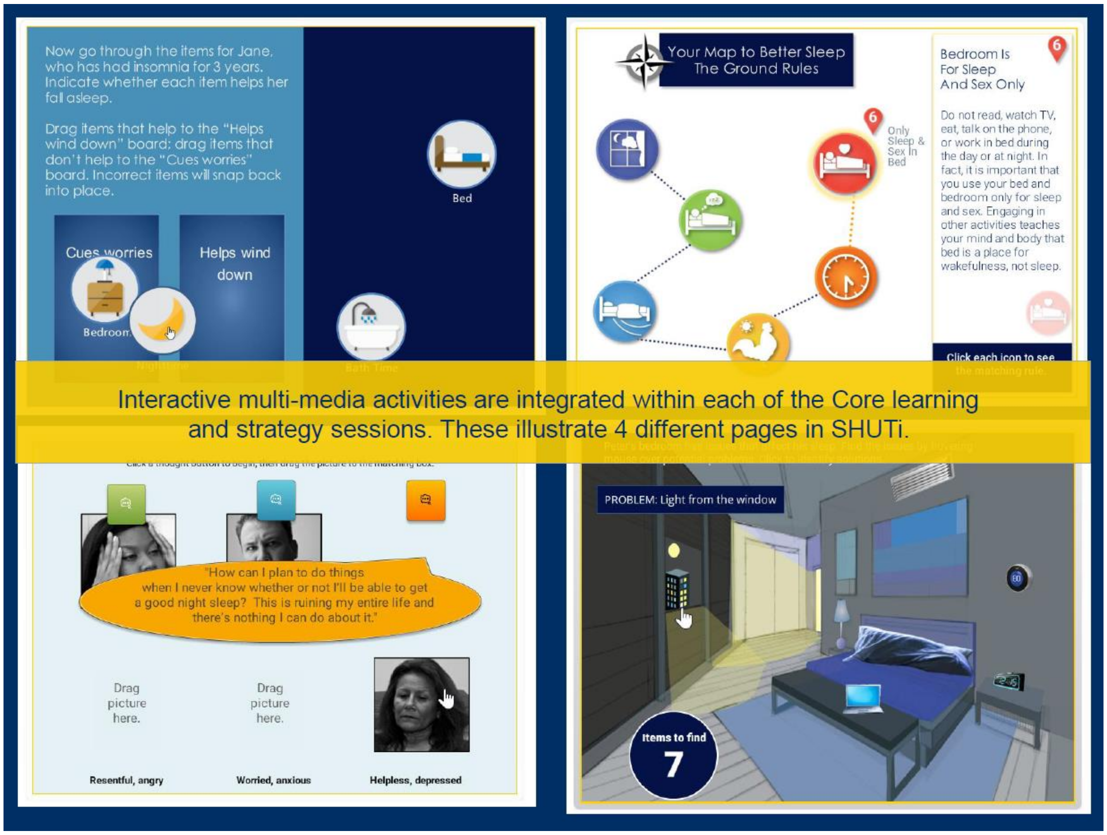

Figure 3. Easy to understand and entertaining quizzes or games assess and improve patient comprehension of training module content.

The Internet-based SHUTi portal is organized into six weekly or bi-weekly scheduled lesson modules, also known as "Sleep Cores", in which participants will be able to self-educate themselves regarding key intervention techniques adapted from in-person traditional CBT-I content. Each Sleep Core is comprised of a lesson plan and test modules intended to intellectually engage participants and encourage content comprehension.

1. *"Getting Ready"* – Introduces participants to the intervention portal and explains the basic user interface, navigation, and what is to be expected as they progress through the program. Collection of sleep diaries begin immediately in order to establish a insomnia severity baseline.
2. *"Sleep Scheduling"* – The technique of Sleep Restriction is introduced including a brief explanation of theory, common questions, and potential challenges. Participants are given an algorithm-determined sleep schedule to follow which is intended to impose a mild, progressively decreasing, period of sleep limitation.
3. *"Sleep Practices"* – Participants are introduced to the concepts of Stimulus Control and asked to assess their current sleep habits for related areas of potential improvement. A brief knowledge assessment is given in the form of true/false or multiple choice style quizzes. An adjustment or continuation of the previously advised sleep schedule is determined based on change in insomnia severity reported in daily diaries.
4. *"Thinking Differently"* – Participants are encouraged to lessen anxiety an anxiety-provoking behaviors regarding their insomnia. Alternative approaches to managing sleepless nights are encouraged. An adjustment or continuation of the previously advised sleep schedule is determined based on change in insomnia severity reported in daily diaries.

5. *“Sleep Hygiene”* – Participants learn about the concepts of Sleep Hygiene and how various seemingly unrelated behaviors, lifestyle activities, and health decisions may be contributing to their insomnia. An adjustment or continuation of the previously advised sleep schedule is determined based on change in insomnia severity reported in daily diaries.
6. *“Moving On”* – Intended to be the final sleep core module in the program, this section reengages participants with techniques introduced in earlier cores based on self-reported challenges and changes in insomnia severity during the intervention period. A comprehensive summary of portal activity, progress, and change in insomnia severity is presented to users. Participants are encouraged to revisit and work through challenging eCBT-I techniques as well as remain self-accountable with daily sleep diaries.

Studies of SHUTi have demonstrated similar efficacy and tolerability to conventional CBT-I in a general population. An early investigation in 2009 of 45 adults suffering from insomnia assigned to intervention (n=22) or control groups (n=23) demonstrated significant improvement in symptoms with decreasing ISI scores from 15.73 (95% CI, 14.07 to 17.39) to 6.59 (95% CI, 4.73 to 8.45) in the active versus control groups, with participants retaining improvements at 3 month follow-up (Ritterband et al., 2009). Participants in the same study demonstrated increases in sleep efficiency and decreases in wake after sleep onset (Ritterband et al., 2009). The beneficial intervention effects of eCBT-I were examined more fully in a 2013 follow-up investigation by an associated team of investigators for their relationship to improvement in quality of life, fatigue, and secondary psychological outcomes resulting from decreased insomnia severity (Thorndike et al., 2013). The randomized controlled trial of 44 participants receiving active eCBT-I (n=22) or control (n=22) found notable secondary improvements in depression, anxiety, and overall mental health for participants receiving active intervention versus the control group (Thorndike et al., 2013). The most recent and robustly designed trial investigating SHUTi was a randomized controlled study conducted with 1149 participants in Australia receiving active intervention (n=574) or control education (n=575) (Christensen et al., 2016). The active group participants received access to the online SHUTi portal for 9 weeks while control participants received access to HealthWatch, an Australia-based online health portal designed to provide an attention-matched placebo without any integrated insomnia or sleep-related interventions, for an equivalent period of time. Researchers showed significant symptom improvements in active versus control groups for the primary outcome of major depression co-occurring with insomnia. Secondary outcomes of insomnia severity, suicidality, generalized anxiety, and overall disability or functional impairment also showed significant improvements (Christensen et al., 2016). These results provide encouraging evidence as to the potential efficacy, relevance, and large-scale feasibility of utilizing eCBT-I within the MHS.

Related Therapies

Other adaptations to traditional in-person CBT-I have been developed in recent years with varying success. Brief Behavioral Therapy for Insomnia (BBTI) was developed to be a shortened adaptation of traditional therapy whereby patients are instructed to complete 4 intervention sessions, 2 of which may conducted via telephone, delivered over a 4-week period. The rationale and advantages to the BBTI adaptation is such that patients may complete intervention in a shorter duration of time with fewer accessibility and logistical burdens, namely access to a scarce network of providers. BBTI has been studied for effectiveness in older adults suffering from insomnia with success approaching traditional in-person therapy (Buysse et al., 2011; Troxel et al., 2013). At present, the majority of investigations studying the efficacy of BBTI have focused on older adult populations, rather than a demographic representative of all military TBI patients, which may limit its generalizability. Additionally, BBTI remains dependent on in-person intervention sessions, which may impose a significant burden on military TBI patients and dissuade them from seeking help.

A smartphone-based application was also developed recently in collaboration with the Department of Veteran Affairs (VA). CBT-I Coach, which aims to provide an interactive service designed to enhance patient experience for those undergoing CBT-I, is a smartphone application capable of capturing self-report outcomes, patient feedback, enhanced communication with providers, and homework modules to improve patient breadth of understanding and overall compliance (Kuhn et al., 2016). A randomized controlled pilot study for CBT-I Coach in 18 patients undergoing standard intervention for clinical insomnia examined the application in terms of feasibility, acceptability, and potential impact on adherence to intervention recommendations and outcomes reporting (Koffel and Kuhn et al., 2016). While patients reported the application as highly usable and noncompromising in terms of benefits of CBT-I, no significant added benefit in terms of patient time spent on homework, number of days completing homework, or number of days completing sleep diaries were observed comparing application-adjunctive versus control/non-application CBT-I intervention groups (Koffel and Kuhn et al., 2016). Another study assessing pre- and post-implementation of the smartphone app within VA found that while a majority of clinicians (87%) believed the app could very likely improve care as initially reported, less than 60% of patients reported using it two years later (Kuhn et al., 2016). An inherent limitation of CBT-I Coach is that the application was developed to serve as an adjunctive or complementary modality along with in-person intervention. Furthermore, CBT-I Coach has not been tested in large randomized trials to our knowledge. These marked disadvantages greatly limit the deployability and benefit of CBT-I Coach. Fully independent intervention platforms such as SHUTi address the shortcomings of both BBTI and CBT-I Coach. Not only is SHUTi designed to act as an independent, stand-alone intervention, its efficacy has been documented in large randomized controlled trials.

Scientific Justification

To date, only one published investigation has compared the use of tele-delivered CBT-I to in-person therapy within a military population to which both interventions were found to be efficacious in reducing insomnia symptoms (Taylor et. al, 2017). This study suggests a general trend that tele-delivered therapies tend to be slightly less effective than traditional in-person therapy. However, this finding must also take into consideration the current lack of clinical resources and subsequent barriers to accessing assistance facing the majority of insomnia patients within MHS.

In addition to improved intervention efficacy and availability for insomnia therapy, eCBT-I may produce significant improvements in secondary outcomes that have particularly significant relevance to military populations. For instance, multiple studies have examined the link between insomnia and depression, PTSD, and suicidal ideation. A large university student cohort (n=1149) study of eCBT-I using SHUTi demonstrated a significant improvement in depression scores as measured by the PHQ-9 at 9 weeks and 6 months following active intervention when compared with controls. (Christensen et al., 2016). In another study, investigators concluded decreased incidence and severity of insomnia symptoms may be an important consideration in shaping suicide prevention plans (Ribeiro et al., 2012). Other studies have demonstrated overlapping neurobiology and symptomology with depression (Benca and Peterson, 2008; Riemann and Voderholzer, 2003), PTSD (DeViva et al., 2004; Koffel and Khawaja et al., 2016), and overall suicide risk (Li et al., 2010).

It is expected that many participants in this study will suffer from longstanding comorbidities that may demonstrate significant cross-outcome improvements as a result of improved sleep and indirectly-related health outcomes. Although outside the scope of this investigation, there is evidence to suggest chronic insomnia increases risk for diabetes (Knutson et al., 2006), heart disease (Phillips et al., 2007), hypertension (Phillips et al., 2007), and higher rates of overall mortality (Parthasarathy et al., 2015). A large scale military trial evaluating the feasibility and efficacy of eCBT-I with comorbid TBI could

therefore provide significant multi-domain benefits to service members as well as inform future models of standard care within MHS.

The investigators have designed the proposed 3:1 imbalanced randomization scheme based on two key benefits. The emphasis of this investigation is to provide potential immediate therapeutic benefit to the greatest number of participants possible to which randomization favoring active intervention facilitates. Additionally, statistical power simulations performed by the study team suggest the greatest potential to detect effects on military-relevant secondary outcomes in PCL-5 and PHQ-9 scores using a proportionally larger active intervention group, while not significantly impairing power of the primary outcome analysis.

Human Participants Justification

CBT-I and other Cognitive Behavioral Therapy (CBT) techniques include a broad toolset of empirically-validated psychotherapeutic interventional strategies based on present understanding of human cognition. The foundation of CBT and related therapies rely on the hypothesis that the way an individual perceives a situation is closely connected to their handling of the situation (Field, Beeson, and Jones, 2015). Therefore, the most important and impactful components of intervention are those that help patients change unhelpful or counterproductive thinking and behaviors in ways that cause either direct or indirect improvement of their condition. These behavioral interventions are the active therapeutic component of CBT intervention for patients suffering from insomnia or other mood-related conditions, and they may be viewed as analogous to the active drug compound in a similarly aimed pharmaceutical intervention. However, unlike many pharmaceutical interventions which represent significant risk in terms of contraindications and potential side effects, CBT-I employs behavioral techniques intended to alter subject lifestyle in a way that is naturally favorable to healthy outcomes. Although various mechanisms have been demonstrated to cause secondary insomnia as a result of foreign substance administration (Richardson, 2007), medical and psychiatric comorbidity (Doufas et al., 2012), or genetic alteration (Revel et al., 2009), there are at present no well-studied or scientifically representative animal models that accurately reproduce the condition of insomnia as experienced by humans. Based on these considerations, the expected benefits of participation in the proposed study of eCBT-I far outweigh the potential dangers of participants not receiving assistance for their insomnia.

Objectives/Specific Aims/Research Questions:

Primary Objective

- To determine the feasibility and efficacy of active eCBT-I compared to education control for insomnia in US military service members with history of TBI.
    - *Primary Hypothesis:* Active eCBT-I intervention will lead to greater reductions of symptoms for insomnia compared to education control in active and retired service members with history of TBI.
    - *Primary Outcome Measure:* Insomnia Severity Index (ISI) (Gagnon et al., 2013; Bastien et al., 2001; Morin et al., 2011)
    - *Primary Analysis (Intention to Treat*): Comparison of changes in ISI scores from baseline to post-intervention between those randomized to active eCBT-I versus education control. A clinically meaningful change will be defined as a >=25% reduction in total symptom score.
    - *Secondary Analyses:*
        - Comparison of changes in ISI scores from baseline to post-intervention between those who fully complete active eCBT-I versus those assigned to education control (“as treated” analysis)

- Comparison of retained change in ISI scores from baseline to 3 month follow-up in intention-to-treat and as-treated analyses.
- Fraction of participants with ISI <15, below threshold for clinically significant insomnia, at post-intervention and 3 month follow-up time points.

Secondary Objectives

- To assess changes in depression symptom severity as reflected by the Patient Health Questionnaire 9 for Depression (PHQ-9) (Kroenke et al., 2001).
  - *Primary Analysis:* Comparison of changes in PHQ-9 scores from baseline to post-intervention between those randomized to active and education control.
  - *Secondary Analysis:* Comparison of changes in PHQ-9 scores from baseline to 3 month follow-up between those randomized to active and education control.
- To assess changes in PTSD-related symptoms as reflected by the PTSD Checklist for DSM-5 (PCL-5) (Belvins et al., 2015).
  - *Primary Analysis:* Comparison of changes in PCL-5 scores from baseline to post-intervention between those randomized to active and education control.
  - *Secondary Analysis:* Comparison of changes in PCL-5 scores from baseline to 3 month follow-up between those randomized to active and education control.
- To assess changes in migraine-related symptoms as reflected by the Migraine Disability Assessment (MIDAS) (Stewart et al., 2001).
  - *Primary Analysis:* Comparison of changes in MIDAS scores from baseline to post-intervention between those randomized to active and education control.
  - *Secondary Analysis:* Comparison of changes in MIDAS scores from baseline to 3 month follow-up between those randomized to active and education control.
- To assess changes in sleep quality as reflected by the Pittsburgh Sleep Quality Index (PSQI) (Grandner et al., 2006) with Addendum for PTSD (PSQI-A) (Germain et al., 2005).
  - *Primary Analysis:* Comparison of changes in PSQI and PSQI-A scores from baseline to post-intervention between those randomized to active and education control.
  - *Secondary Analysis:* Comparison of changes in PSQI and PSQI-A scores from baseline to 3 month follow-up between those randomized to active and education control.
- To assess changes in fatigue-related symptoms as reflected by the Functional Assessment of Chronic Illness Therapy-Fatigue (FACIT-F) (Butt et al., 2013)
  - *Primary Analysis:* Comparison of changes in FACIT-F scores from baseline to post-intervention between those randomized to active and education control.
  - *Secondary Analysis:* Comparison of changes in FACIT-F scores from baseline to 3 month follow-up between those randomized to active and education control.
- To assess changes in sleep diary outcomes and calculated sleep efficiency: bed time, sleep onset latency, number of awakenings, total duration of awakenings, wake time, arising time, daytime naps, soundness of sleep, sleep quality, and sleep medication or alcohol use.
- To assess investigator blinding efficacy as reflected by mid-intervention and post-intervention questionnaires to be completed by the study team.
- To assess participant expectation of benefit and blinding efficacy as reflected by pre-intervention and post-intervention questionnaires.
- To assess concurrent medications, psychotherapeutic therapies, or lifestyle changes, specific CBT-I training techniques, and their correlation with intervention efficacy.
- To assess participant satisfaction and help-seeking behavior as reflected by a 3 month follow-up questionnaire.

Study Design:

Internet-based, double-blinded, controlled, prospective, randomized interventional trial with an optional open-label intervention. Up to 200 participants will be randomized to either eCBT-I or education control groups in a 3:1 ratio, respectively.

Target Population:

Study design is intended to be as inclusive as possible for all interested participants while retaining basic scientific and logistical controls. Results from this study are expected to be specifically relevant for patients within MHS suffering from insomnia with history of TBI.

Benefit to the DoD:

Strong empirical evidence in recent years supporting the use of CBT-I intervention for insomnia has led to its adaptation as a first-line intervention for many institutions and healthcare professionals (Siebern & Manber, 2011; Taylor & Pruiksma, 2014). Unfortunately, this recommendation has not been fully implemented within the MHS standard of care due to the geographic complexities of military life, limited numbers of trained CBT-I providers, and heavily burdened psychological health centers. For these reasons, current therapeutic strategies favor pharmacological interventions directed at primary psychiatric comorbidities such as major depression disorder, PTSD, and suicidality. A remotely-accessible intervention platform would enable dramatically greater numbers of MHS patients to benefit from available intervention options.

ECBT-I intervention has the inherent benefits of improved safety and limited side effects when compared to currently utilized drug therapies (Mitchell et al., 2012). This may be of particular benefit to active and retired service-members suffering from previous injuries that require multiple long-term drug therapies and potentially side effects or contraindications.

ECBT-I also presents an opportunity to reduce the personnel and financial burden within MHS. A standard intervention course of self-guided eCBT-I costs approximately $75 per patient, whereas traditional therapy costs $1200 to $1800 per patient with weekly 2-hour sessions for a period of 6 or more weeks. A recent cost analysis of TBI care within the VA estimated total expenditure to be $2.2 billion in the coming 10 years (FY2016-FY2025) (Bagalman, 2015). As noted, eCBT-I is expected to significantly improve insomnia as well as secondary outcomes relating to depression and PTSD. These combined maladies represent a significant portion of the resource and financial cost of TBI within MHS.

The UVA will consult with the investigators in developing a customized investigational plan using the online SHUTi portal. As part of the customization processes, standard patient vignettes, testimonials, and other online materials previously developed for a non-military-specific audience will be modified to be appropriate for the targeted intervention population. The modifications also include direct integration of study outcome measures in the form of paired online pop-up windows through the partnering survey provider company, Qualtrics (333 West River Park Drive. Provo, UT 84604). This will allow for an intuitive online environment that is easy for participants to complete as they navigate through the SHUTi portal. For the proposed investigational study, integrated Qualtrics forms will be tailored to collect specific outcome measures relying on self-report in a scalable and standardized format that meets 21 CFR Part 11 and HIPAA-compliant information system security guidelines. It is worth noting that the proposed investigation is entirely funded by the CNRM and is not privately sponsored or supported by external parties. Services at UVA and Qualtrics are being engaged at-cost and as such may not put any restrictions or contingencies on the analysis, dissemination, or publication of study findings regardless of the outcomes.

If the expected positive outcomes are obtained, the results of this study may inform clinical policy and support the immediate implementation of eCBT-I as a first-line intervention for insomnia within the

MHS. This potential change in standard of care would have a tremendous impact on quality of life for active and veteran service members suffering from insomnia.

STUDY PROCEDURES AND DATA MANAGEMENT

Study Procedures:

Describe step-by-step how the study will be conducted from beginning to end

Procedures

This study will be a double-blinded, controlled, prospective randomized trial with up to 200 participants. An overview of the participant timeline is presented below.

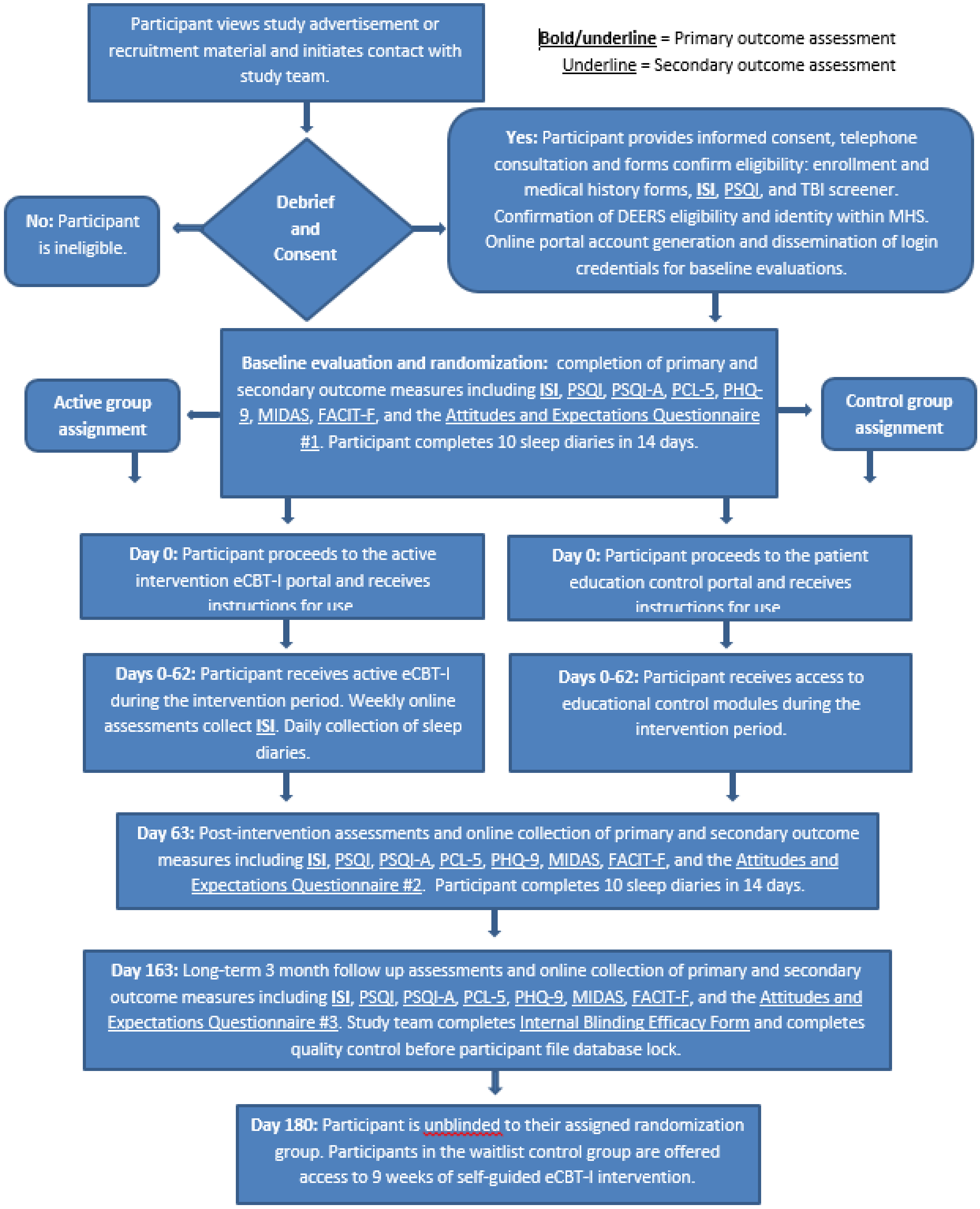

Baseline Evaluation

Participants will complete their baseline evaluation after consent and upon enrollment into the study. Successful completion of the baseline evaluation will be documented in two processes: (1) a telephone consultation following consent that emphasizes screening of relevant medical history and

inclusion-exclusion criteria and (2) primary and secondary outcome assessments administered through the online portal. All assessments and forms have been submitted with this proposal. For a description of each measure, see the section titled 'Data Collection'.

The baseline evaluation will include:

- Review and electronic signature of Informed Consent for participation and medical review
- Enrollment Forms
    - Participant Contact Information
    - Demographics Questionnaire
    - Global Unique Identifier (GUID) Request
- Medical History Forms
    - Basic Medical History including medications, current, and previous therapies for insomnia or other sleep disorders
    - TBI Screener
- Primary Outcome Assessment
    - Insomnia Severity Index (ISI)
- Secondary Outcome Assessments
    - Posttraumatic Stress Disorder Checklist for DSM-5 (PCL-5)
    - Patient Health Questionnaire 9 (PHQ-9)
    - Pittsburgh Sleep Quality Index (PSQI)
    - Pittsburgh Sleep Quality Index Addendum for PTSD (PSQI-A)
    - Migraine and Disability Assessment (MIDAS)
    - Functional Assessment of Chronic Illness Therapy for Fatigue (FACIT-F)
    - Attitudes and Expectations Questionnaire

To remain clinically pragmatic and inclusive to all interested patients, participants will not be required to discontinue current therapy for insomnia or concurrent conditions and will be advised to continue following all recommendations from their standard of care clinical provider. This includes continuation of any medication regimen or behavioral therapy, so long as the participant has not currently or previously received CBT-I intervention and meets all other inclusion and exclusion criteria. Participants not meeting the eligibility criteria as assessed during the baseline evaluation will be documented as screen failures. Furthermore, patients will not be required to use actigraphy to monitor sleep because of the pragmatic challenges of doing so and the generally good correlations between actigraphy and self-report measures of insomnia.

The baseline phone evaluation will require approximately 1 hour to complete including review of informed consent, completion of enrollment and medical history forms, medications, and TBI Screener. Participant access to the online portal login and credentials is expected to take approximately 3 days for account generation. Following account generation, participants will be prompted to login to the online portal, confirm their access, and complete baseline surveys for the primary and secondary outcome assessments. The entire process of baseline evaluation from time of consent to collection of all outcome assessments is expected to take approximately one week.

Intervention Period Evaluations (Days 0-62)

Eligible participants will be randomly-assigned by computer to active or education control groups in a 3:1 ratio, respectively. A block randomization schedule will be developed to ensure balanced group assignments. There are too many potentially interested clinical subgroups to allow meaningful blocked randomization (for additional details, see section titled 'Statistical Analysis Plan') and no evidence to date that specific subgroups of patients respond differentially to CBT-I. The randomization schedule will be maintained by the study data manager and will link participants coded study identification numbers with their randomized group assignment.

The regulatory monitor will be blinded to group assignment unless otherwise required due to safety and reporting considerations. The principal investigator (PI), biostatistician, and all study personnel interacting with participants will be blinded to group assignment as well. The data manager will be unblinded to group assignment and responsible for final data quality control.

Study participants will routinely access the online portal during the intervention period allowing them to complete study modules and outcome assessments specific to their assigned study group schedule.

Arm 1, active CBT-I intervention:

1. Participants receive 9 weeks of active CBT-I through the online program. As part of the program, participants receive self-guided educational reading modules, patient testimonials and vignettes, and brief quizzes to improve learning and comprehension of key CBT-I techniques. The online portal is designed for a personal computer or tablet device but works well on most modern smartphones with large screens. Participants will be able to complete the active eCBT-I in as little as 6 weeks. The 9-week window is designed to allow flexibility in situations where participants' ability to complete eCBT-I related activities may be temporarily interrupted. See Figures 1-3 regarding example content accessible through the active intervention portal.
2. Participants are instructed to complete daily sleep diaries including self-reported bed time, sleep onset latency, number of awakenings, total duration of awakenings, wake time, arising time, daytime naps, soundness of sleep, sleep quality, and sleep medication or alcohol use.
3. The online portal will require participants to complete sleep diaries a minimum of 5 days each week for the beginning and final two weeks, or for 4 weeks cumulatively, of the intervention period.
4. The online portal will require participants complete the ISI weekly during the intervention period.

Arm 2, patient education control program:

1. Participants are given 9 weeks access to the patient education control program, an online portal designed to inform participants about healthy lifestyle activities and general insomnia information. Participation in the control portal provides participants with access to information about insomnia that they would externally access through websites such as WebMD or The National Sleep Foundation homepage. Education control group participants are requested to read educational modules to control for attention-matching when compared to active eCBT-I intervention. User experience, design, and logistical details for the control portal are designed by the study team to match all components of the active group procedures excluding utilization of established CBT-I intervention techniques. Participant experience in the control portal is non-dynamic and does not provide ongoing feedback in response to outcome assessment results or reported insomnia symptoms. See Figures 4-6 regarding example content accessible through the education control portal.
2. The online portal will require participants to complete sleep diaries a minimum of 5 days each week for the beginning and final two weeks, or for 4 weeks cumulatively, of the intervention period.

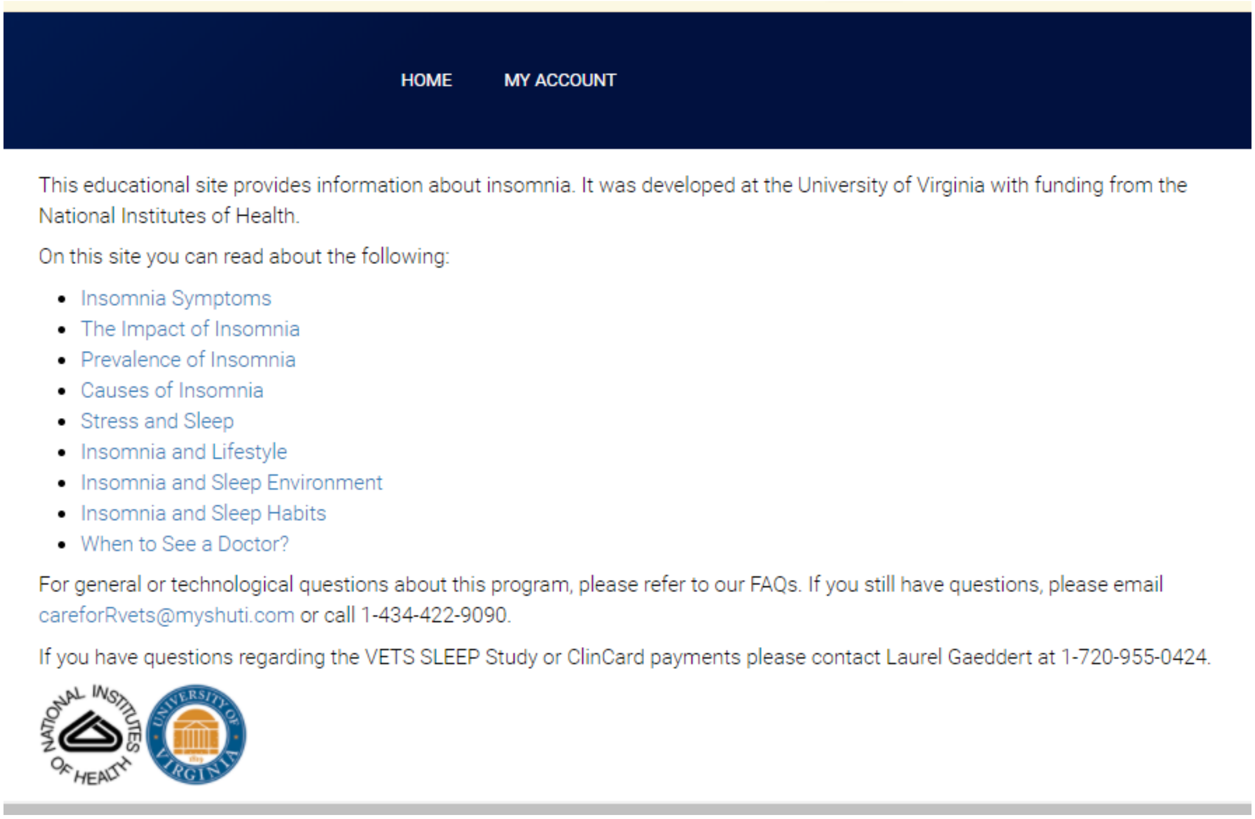

Figure 4. Example educational pages available to education control participants through the online education control portal. Information available is generalized and non-specific to the subject based on reported insomnia symptoms and severity.

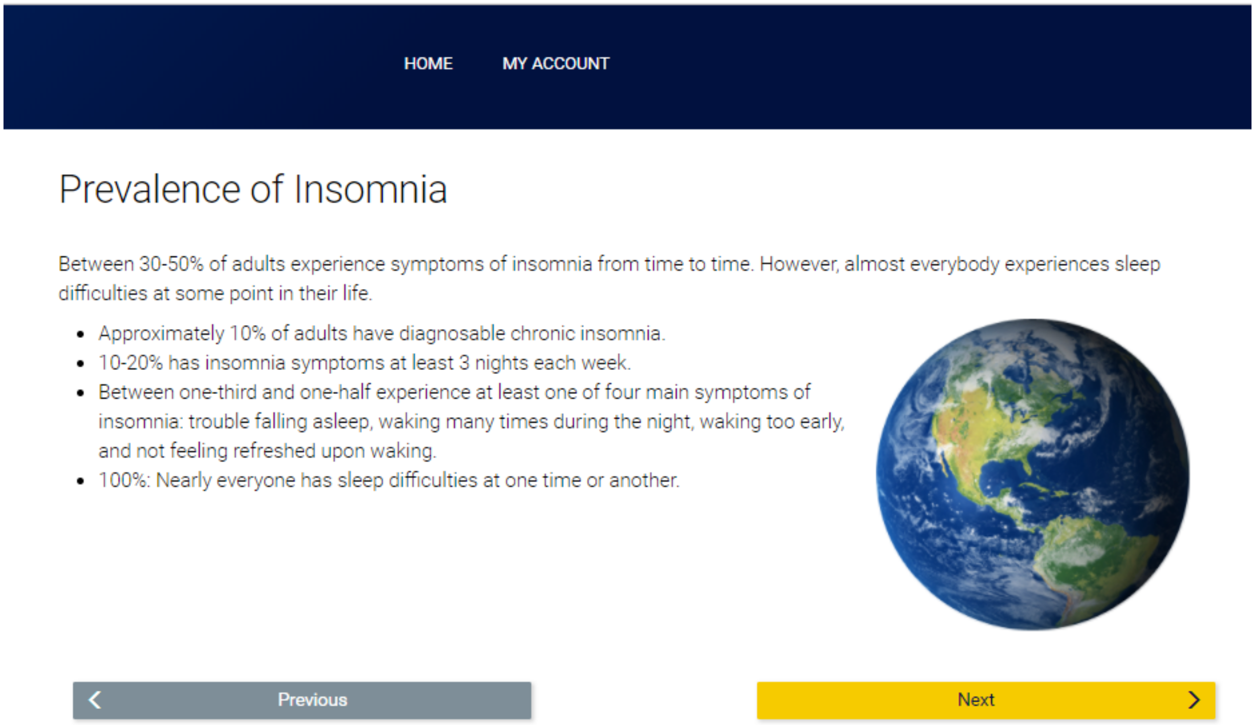

HOME MY ACCOUNT

Prevalence of Insomnia

Between 30-50% of adults experience symptoms of insomnia from time to time. However, almost everybody experiences sleep difficulties at some point in their life.

- Approximately 10% of adults have diagnosable chronic insomnia.
- 10-20% has insomnia symptoms at least 3 nights each week.
- Between one-third and one-half experience at least one of four main symptoms of insomnia: trouble falling asleep, waking many times during the night, waking too early, and not feeling refreshed upon waking.
- 100%: Nearly everyone has sleep difficulties at one time or another.

Previous Next

Figure 5. Example educational pages available to education control participants through the online education control portal. Information available is generalized and non-specific to the subject based on reported insomnia symptoms and severity.

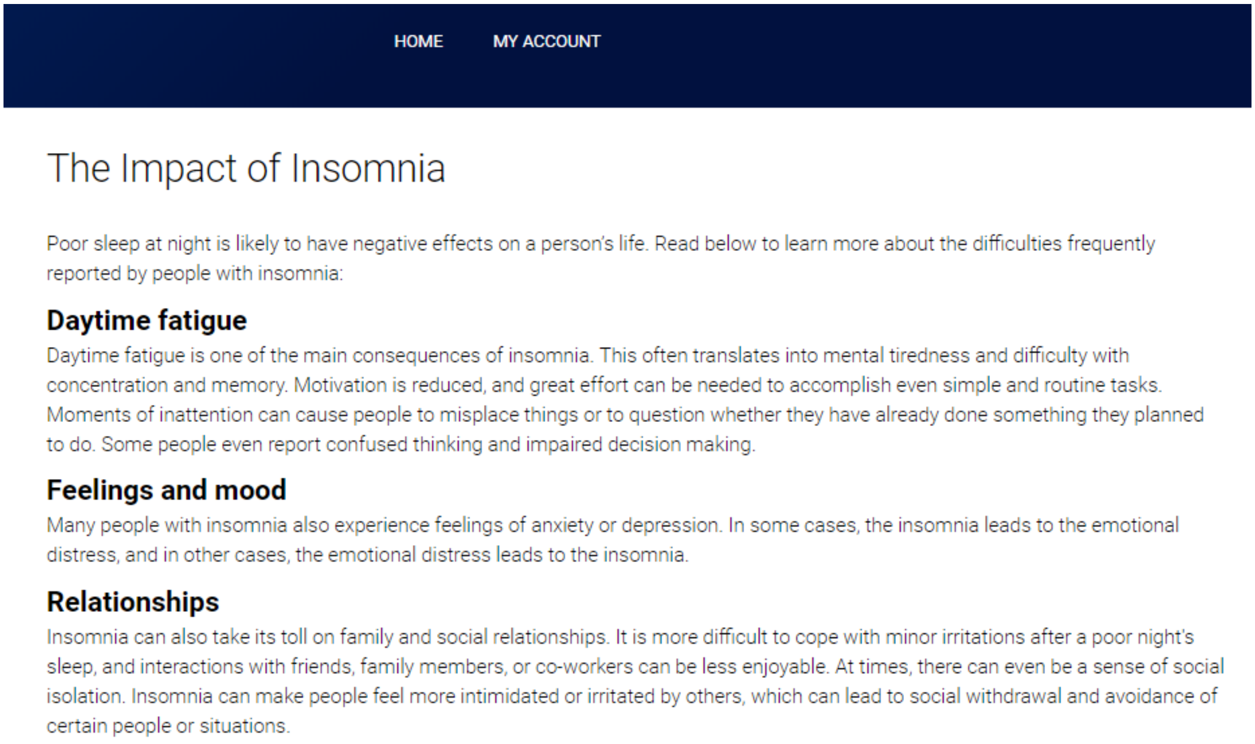

HOME MY ACCOUNT

The Impact of Insomnia

Poor sleep at night is likely to have negative effects on a person's life. Read below to learn more about the difficulties frequently reported by people with insomnia:

Daytime fatigue

Daytime fatigue is one of the main consequences of insomnia. This often translates into mental tiredness and difficulty with concentration and memory. Motivation is reduced, and great effort can be needed to accomplish even simple and routine tasks. Moments of inattention can cause people to misplace things or to question whether they have already done something they planned to do. Some people even report confused thinking and impaired decision making.

Feelings and mood

Many people with insomnia also experience feelings of anxiety or depression. In some cases, the insomnia leads to the emotional distress, and in other cases, the emotional distress leads to the insomnia.

Relationships

Insomnia can also take its toll on family and social relationships. It is more difficult to cope with minor irritations after a poor night's sleep, and interactions with friends, family members, or co-workers can be less enjoyable. At times, there can even be a sense of social isolation. Insomnia can make people feel more intimidated or irritated by others, which can lead to social withdrawal and avoidance of certain people or situations.

Figure 6. Example educational pages available to education control participants through the online education control portal. Information available is generalized and non-specific to the subject based on reported insomnia symptoms and severity.

Post-intervention Evaluation (Day 63)

The post-intervention evaluation includes a repeat evaluation of the primary and secondary outcome measures that were conducted at the baseline evaluation. Scheduling of this evaluation into single or several online sessions is permissible to accommodate participant availability so long as all data is collected within 10 days of intervention completion.

3 Month Follow-Up Evaluation (Day 163)

The 3 month follow-up evaluation includes a repeat evaluation of the primary and secondary outcome measures that were conducted at the baseline evaluation. Scheduling of this evaluation into single or several online sessions is permissible to accommodate participant availability so long as all data is collected within 10 days of the 3 month time point following intervention completion. The study team will also complete the internal blinding efficacy form immediately following completion of the 3 month follow up evaluation.

Open-Label Intervention (Day 180):

After completion of the 3 month follow-up evaluation and internal blinding efficacy form, data regarding each participant is given a quality control review prior to participant finalization (e.g., participant file database lock). Quality control reviews and participant file database locks are performed on a rolling basis as the study progresses. After data locking, participants are then contacted and unblinded as to their group assignment. Participants randomized to the education control group will be offered free-of-charge access to open-label eCBT-I intervention for a period of 9 weeks. No additional data collection will be performed following completion of a participant's data lock and unblinding. However, the study team will facilitate continued technical and logistical support for participants deciding to participate in the open-label intervention. Once a given participant completes the main study period, the blind is broken and if the participant had been randomized to the control group they will be offered the actual intervention.
In order to preserve the equipoise of the study team, until the trial is completed (i.e each patient has completed participation and the data have been cleaned) no efficacy analyses of aggregate data will be conducted, other than the interim analyses already specified in the protocol.

Team Member Roles and Responsibilities:

Name	Responsibilities	Access to identifiable information?
David Brody	Principal investigator, protocol design and overall management, design of intervention portal, interpretation results, handling of safety events	Yes
Martin Cota	Associate Investigator, protocol design, clinical interaction blinded to randomization, recruitment, interpretation of results, regulatory correspondences	Yes
Alura Johnston	Associate Investigator, protocol design, clinical interaction unblended to randomization, recruitment, interpretation of results, regulatory correspondences	Yes
Baharer Kost	Associate Investigator, primary database designer and data quality control	Yes

Kent Werner	Associate Investigator, protocol design, scientific advisor, interpretation of results	No
Thaddeus Haight	Associate Investigator, protocol design, scientific advisor, statistical design, interpretation of results	No
Lee Ritterband	Collaborator at University of Virginia, design of intervention portal, protocol design, scientific advisor, interpretation of results, point of contact regarding administrative matters at the UVA and Qualtrics	No

Data Collection:

Method of Collection from Participants and Investigators
The following types of information will be collected by study investigators. Assessments will be administered for research purposes only.

A description of each data type will follow:
- Enrollment Forms
- Medical History Forms
- Primary Outcome Assessment
- Secondary Outcome Assessments
- Internal Blinding Efficacy Form

Enrollment Forms
Timepoint: baseline (within 10 days prior to initiating intervention)
Data Gathered: Enrollment forms are designed to collect participant demographic information. Forms will be completed after consenting during a baseline telephone interview and will be labeled with coded patient identifiers and dates of collection.

- *GUID Request*: The GUID is used to assign an ID number to the participant. The process for assigning a GUID is described in the section 'Managing Data (Data Management and/or Sharing Plan) for this Study'. The self-report form will take 1-2 minutes to complete.
- *Participant Contact Information*: The Participant Contact Information form collects emergency and participant contact information to be used throughout their participation including name, primary and secondary phone numbers, email address, and physical address. Information on additional contact sources (family members, friends, other service members, etc.) will be requested in order to increase the likelihood of complete data collection at later time points. However, provision of additional contact source information will be optional. The self-report form takes approximately 3-5 minutes to complete.
- *Demographics Questionnaire*: The Demographics Questionnaire contains questions pertinent to this research effort, including: educational background, military service, etc. The self-report form will be labeled with coded patient identifiers and dates of collection.

Medical History Forms
Timepoint: baseline
Data Gathered: Medical history forms are designed to collect participant medical history relevant to the study inclusion/exclusion criteria and specific aims. Forms will be completed after consenting and transcribed via telephone interview labeled by coded patient identifier and date of collection.

- *Basic Medical History*: The Basic Medical History Form will collect general medical information from participants including: medical history, concomitant medications/therapies, injury-related outcomes, and medication history. The basic medical history form will also include elements of the STOP-Bang questionnaire (Ji and Kang, 2017; Naqappa et al., 2015), which assesses for other sleep-related conditions such as obstructive sleep apnea (OSA) and rapid-eye movement sleep behavior disorder (RBD). The form will be completed via telephone by trained study personnel. Participants with personal copies of their medical records may voluntarily provide such records to study personnel as a supplement to medical history review. Patients who screen positive for untreated OSA will be instructed to seek a definitive diagnosis from their clinical care providers.
- *TBI Screener*: The TBI Screener Form is designed to elicit information regarding the participant TBI(s). The form utilizes the Ohio State University Traumatic Brain Injury Identification Method (OSU-TBI-ID) (Corrigan and Bogner, 2007) as its standardized procedure for obtaining the lifetime history of participant TBI. The screener will be administered by trained study personnel and will take approximately 15-20 minutes to complete.

Primary Outcome Assessment

Timepoints: (1) baseline; (2) weekly during the intervention period; (3) post-intervention, approximately 9 weeks following consenting; (4) 3 month follow-up.

Data Gathered: The primary outcome assessment will be completed within the online portal and will be labeled by coded patient identifier and date of collection.

- *Insomnia Severity Index (ISI)*: The ISI is an extensively validated self-report questionnaire designed to assess the presence and severity of insomnia sleep disorder and is empirically-validated in general and military populations (Gagnon et al., 2013; Bastien et al., 2001; Jenkins et al., 2015; Morin et al., 2011). An ISI score of approximately 15 or greater has been demonstrated to be an appropriate cutoff for confirming the presence of mild clinical insomnia (Morin et al., 2011; Gagnon et al., 2013). The ISI takes approximately 3-5 minutes to complete.

Secondary Outcome Assessments

Timepoints: (1) baseline; (2) post-intervention, approximately 9 weeks following consenting; (3) 3 month follow-up.

Data Gathered: Self-report measures will be completed via within the online portal and be labeled by coded patient identifier and date of collection.

- *Functional Assessment of Chronic Illness Therapy – Fatigue (FACIT-F)*: The FACIT-F is a self-report questionnaire designed to assess symptoms of physical and emotional fatigue in a variety of different illnesses (Butt et al., 2013). The FACIT-F takes approximately 5-10 minutes to complete and will be administered via electronic survey within the online portal.
- *Pittsburgh Sleep Quality Index (PSQI) with Addendum for PTSD (PSQI-A)*: The PSQI-A is a modified version of the well-validated PSQI self-report questionnaire (Grandner et al., 2006) that includes additional sleep quality and insomnia symptoms specifically in participants with diagnosed or suspected PTSD such as nightmares (Germain et al., 2005). A PSQI score of 5 or greater has been demonstrated as a reasonable cut-off for confirming the presence of clinical insomnia (Grandner et al., 2006). The PSQI and PSQI-A take approximately 5-10 minutes to complete and will be administered via electronic survey within the online portal.
- *Patient Health Questionnaire 9 for Depression (PHQ-9)*: The PHQ-9 is a self-report assessment for signs and symptoms associated with major depression disorder (Kroenke et al., 2001). The PHQ-9 takes approximately 3-5 minutes to complete and will be administered via electronic survey within the online portal.
- *PTSD Checklist for DSM-5 (PCL-5)*: The PCL-5 is a self-report questionnaire designed to assess symptoms of PTSD (Belvins et al., 2015). The PCL-5 takes approximately 5-10 minutes to complete and will be administered via electronic survey within the online portal.

- *Migraine Disability Assessment (MIDAS)*: The MIDAS is a self-report questionnaire designed to assess migraine headache frequency, duration, severity, and life impact (Stewart et al., 2001). The MIDAS takes approximately 5-10 minutes to complete and will be administered via electronic survey within the online portal.
- *Attitudes and Expectations Questionnaire*: The Attitudes and Expectations Form will collect qualitative information from participants as to their expected benefit from participating in this study (collected at baseline), believed group randomization assignment (collected post-intervention), and subjective perceived benefit from eCBT-I intervention (collected at 3 month follow-up). The form will also include general questions regarding participant satisfaction after having completed the study. This form takes approximately 5-10 minutes to complete.

Internal Blinding Efficacy Form
Timepoint: Following completion of participant 3 month follow-up evaluation, ±10 days, and preceding participant file database lock and offering of open-label intervention.
Data Gathered: Study investigators will complete with coded patient identifiers and dates of collection.

- *Internal Blinding Efficacy Form*: The Internal Blinding Efficacy Form will document the study team's believed participant group randomization assignment prior to participant file database lock. This form takes approximately 1 minute to complete.

Electronic Capture Outcome Forms
All electronic capture outcome forms will be completed through the SHUTi portal via integrated Qualtrics questionnaires. This information shall be hosted and temporarily stored in a coded database format on remotely-located Amazon Web Services (AWS) servers. The UVA will be responsible for the initial setup of the electronic capture outcome forms database and ongoing maintenance will be managed by the study team. During the intervention period and for each participant, the study team will routinely pull data from the server and transcribe the data to secure electronic and paper records stored internally at the CNRM. Following a participant's completion of the intervention period, a final quality control check will be performed for all study records and outcomes before scrubbing the coded data from the AWS server. Further information regarding data flow and sharing processes is included in protocol section 10.14 and the appendix item 'Data Sharing Application Agreement Draft'.

If you are obtaining SSNs, provide a justification as to why and explain why a substitute cannot be used

The study team must collect participant social security numbers (SSN) for the purpose of verifying DEERS eligibility and GUID issuance.

Managing Data (Data Management and/or Sharing Plan) for this Study:

Data Collection procedures are outlined in the section titled 'Data Collection'.

Enrolled participants will be assigned a unique study ID consisting of the prefix, 'Insomnia-1-, followed by a random three digit number. A Master List linking enrolled protected health information (PHI) to the study ID will be stored in a password-protected database within a secure DoD server. Hard copies of the list with randomization assignment will be stored in a locked cabinet and individually sealed participant envelopes, within a locked office at the CNRM headquarters. Access to the database and list will be provided to approved study personnel.

Enrolled participants will also be assigned a GUID. The CNRM GUID is a number assigned by the CNRM Informatics Core. The Informatics Core has established an encrypted system and will provide

access to the site for generation of a GUID from PHI data. A Master List matching GUIDs to PHI will be maintained on a DoD server with access limited to designated study personnel. Electronic Master List records will be backed up electronically at least monthly. The mapping from PHI to GUID will not be stored by or known to the CNRM Informatics Core or NIH Center for Information Technology personnel, but the central registration of issued GUIDs will help ensure uniformity of identifiers across sites. CNRM Master Lists will contain the following information: GUID, last name, SSN, date of birth, and/or medical record number. Participants will retain initially-assigned study IDs and GUIDs throughout their participation. All data will be stored and linked to the study ID and/or GUID. The Master List will be kept in a secure location for a period of 5 years after study closeout and will then be securely destroyed.

As part of the enrollment process, potential participants will undergo a telephone and electronic consenting process using a customized Google Forms online portal following 21 CFR Part 11 and HIPAA-compliant guidelines. The Google Forms consenting portal will be hosted under the secure USU Google domain. Electronic records of the online consents form database will be housed on a secure DoD server at USU. Back-up hardcopies for all informed consent forms will be stored in a securely locked room and cabinet at the CNRM Twinbrook administrative offices with access limited to approved study personnel.

Data collected may be captured via telephone or in web-based format. Records acquired via telephone and transcribed by the study team to paper format will be stored in a locked cabinet, in a locked room, at CNRM with access of documents provided to approved study personnel. Records acquired via telephone and transcribed to PDF or electronic format will be stored in a secure DoD server, with access of documents provided to approved study personnel. Secure electronic data capture (EDC) will be completed by the study team via the CNRM Informatics Core System. Although collaborators at UVA will retain administrative access to the SHUTi portal for the purposes of development and testing, all Internet services will be hosted externally via Amazon Web Services (AWS) and Qualtrics. Deidentified and coded outcome measures collected via AWS and Qualtrics data servers will be transcribed by the CNRM study team onto the CNRM Informatics Core System which will serve as the master electronic study record. All data being stored in CNRM Informatics Core System will undergo quality control processing by the data manager before being entered. Data entered into the system is then transmitted via Secure Sockets Layer to a database protected by firewall. Although behavioral outcome assessments will be personal in nature, no identifiable information except a temporary session-based IP address will be captured by AWS and Qualtrics data servers. AWS and Qualtrics data servers will not retain participant IP addresses or identifiable information and will instead solely use coded study ID numbers as means of communication between each other and the study team. Parties at UVA, AWS, and Qualtrics will at no point have access to directly identifiable information. Names and electronic signatures of participants will be collected on Google Forms under the secure USU Google domain as part of the consenting process. After completion of consent and printing of hardcopy records, electronic records will be promptly deleted from the USU Google Forms domain. Access to all study data will be provided only to approved study personnel.

Data transcribed to paper may be scanned as needed and saved on the DoD secure network for record keeping. Documents collected via web-based format or transcribed to electronic format may be printed and stored in a locked room and cabinet at CNRM.

To protect participant confidentiality, documents will be labeled with the study ID, time point, and/or date of interaction (for more detailed information regarding labeling, see section ‘Data Collection’. Research records will be kept indefinitely.

At the conclusion of the study, the CNRM Informatics Core will provide for the identifier-free research records to be transferred and stored in the Federal Interagency Traumatic Brain Injury Research (FITBIR) and the CNRM Data Repository, as appropriate.

Confidentiality of records will be protected to the fullest extent possible. However, information regarding participants' health may be required to be reported to appropriate medical and/or command authorities per participant safety and legal requirements. The reporting of sensitive information (e.g., suicidal ideation) by military personnel participating in the study will be reported to their Commanding Officer. In these instances, referral sources for assistance will also be provided.

Participants will be informed that these results may be published for scientific purposes, provided their identity is not revealed. Members of CNRM, sponsor representatives, Uniformed Services University, Henry M. Jackson Foundation, US Department of Defense, and NIH may have access to the study data for auditing purposes. At no point during this investigation will Defense Health Agency (DHA) data be transferred outside of the DHA. Data agreements will be included as deemed necessary.

Managing Data for Future Research:

Data collected throughout the study may be used for future research. Identifier-free data will be shared with the following repositories: the CNRM Data Repository and the FITBIR Database.
The CNRM Data Repository is a data repository that contains a collective of de-identified research data from CNRM funded studies. The FITBIR database is an informatics system and central data repository developed by DoD and NIH to store and link together phenotypic, diagnostic, treatment, and outcome data derived from persons who participated in TBI research studies. To protect privacy and confidentiality, data stored in these repositories will be linked to the participant's GUID. Data will stored in these repositories indefinitely. During the consent process, participants will be informed that their data may be used for future research. Participants will also be informed that this data may be used for a variety of research purposes that may not be able to be specified during consent time. Before a dataset is shared, identifiable information will be redacted and stored as a coded dataset until the time the Master List link is destroyed. The Master List link connecting GUIDs to identifiable information will remain with approved study personnel.

The CNRM Informatics Core will provide for the identifier-free research records to be transferred and stored in the FITBIR and/or the CNRM data repositories, as appropriate. Identifiers will be removed and then transferred into the FITBIR and/or the CNRM data repositories database, as appropriate. All elements of PHI and personal identifying information (PII) will be removed prior to the sharing of data with the CNRM Data Repository and the FITBIR database. Access to the data located in the CNRM Repository will be determined by the CNRM Data Quality, Access, and Publication Committee. Investigators requesting access to the data located in the CNRM Data Repository are expected to provide the committee a list of investigators and collaborators who will have access to the data along with documentation of Ethical Conduct of Research and Human Participants Protection Training, as well as documentation of IRB approval of the research project.

Data in FITBIR will be open for access through the FITBIR system to qualified researchers who have requested access to the data.

As required by U.S. Law, this study will be registered with ClinicalTrials.gov prior to initiation of recruitment. On an ongoing basis and following project completion, updated results and study status will also be provided to the site.

STATISTICAL ANALYSIS PLAN

Primary Outcome

Change in ISI score pre- versus post-intervention represents the primary outcome measure of interest. Analyses will be based on intention-to-treat (ITT) as it pertains to participant's original group assignment. First, an initial standard analysis will examine the ISI change between week 1 (pre-intervention) and week 6-9 (post-intervention) between the active and control groups. Assuming ISI measures are normally distributed, the following mixed model could be applied to assess changes in ISI score post-intervention compared to pre-intervention in active and control groups:

$$E[Y_{ijk}] = \alpha_i + \beta_0 + \beta_{1j}\,\text{Intervention} + \beta_{2k}\,\text{Time} + \beta_{3jk}\,\text{Intervention x Time}$$

… where Y_{ijk} represents ISI score in the *i*th person, *j*th group (1=active, 0=control) and *k*th timepoint (1=6-9 weeks, 0=1 week). Based on the model, β_1 represents the mean difference in ISI score between groups at week 1, β_2 represents the mean difference in ISI at 6-9 weeks vs. 1 week in the control group, and β_3 represents the mean difference in ISI score at 6-9 weeks vs. 1 week in the intervention relative to the control group. A two-sided test with alpha=0.05 would test the significance of the intervention x time effect represented by the β_3 coefficient. Additional parameters in the model include β_0 which represents the mean ISI score in the control group at 1 week and α_i which represents an individual's random effect (i.e., ISI measure at week 1) to account for within-subject correlation.

Given subject-to-subject variability with respect to completion of the eCBT-I protocol within the intervention window period ranging between 6 to 9 weeks, an additional analysis of group differences in ISI scores will be examined using area under the curve (AUC). Based on AUC methods proposed by Faraone et al. (2000), the AUC method applied to the current study would examine ISI differences (week 6-9 – week 1) - i.e., negative differences would indicate symptom improvement - between the active and control groups. Specifically, differences in ISI score (D) for the entire study group would be ranked from lowest (i.e. negative) to highest (positive differences). For each D, the cumulative percentage of respondents from the active and control groups would be determined, plotted with respect to y and x axes representing proportion of respondents in these two groups, and an intervention-response curve would be drawn (See Figure 7 below courtesy of Faraone et al.). The AUC, measured with respect to this intervention – response curve, will be examined with respect to AUC=0.5 (i.e., null difference), as typically reported in ROC analyses, using a two-sided test and α=0.05.

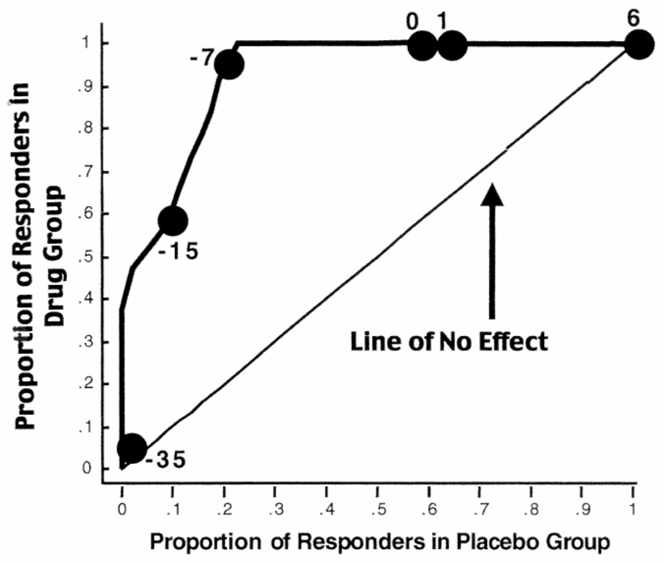

Figure 7. Example intervention response curve between proportion responders in active and control groups (Faraone et al., 2000). The protocol herein does not include a drug group.

A separate analysis will examine ISI differences between week 1 and 3 month follow-up following intervention. Similar to the analysis above that examined pre- versus post-intervention differences in ISI score, the following mixed model could be applied to assess changes in ISI score at 3 month follow-up compared to week 1 between the active and control groups:

$$E[Y_{ijk}] = \alpha_i + \beta_0 + \beta_{1j}\,\text{Intervention} + \beta_{2k}\,\text{Time} + \beta_{3jk}\,\text{Intervention x Time}$$

… where Y_{ijk} represents ISI score in the *i*th person, *j*th group (1=active, 0=control) and *k*th timepoint (1=3 months, 0=1 week). Based on the model, β_1 represents the mean difference in ISI score between groups at week 1, β_2 represents the mean difference in ISI at 3 months vs. 1 week in the control group, and β_3 represents the mean difference in ISI score at 3 months vs. 1 week in the intervention relative to the control group. Similarly, a two-sided test with α=0.05 would test the significance of the intervention x time effect represented by the β_3 coefficient, and additional parameters would be included as previously shown.

Secondary Outcomes

Change in measures considered to be associated with ISI that could potentially be affected by the CBT-I intervention will be examined using different models for the intervention group. Initially, these different secondary outcomes will be examined for normality. In the case that the measures are normally distributed, a mixed model similar to the one below would be applied to examine differences in the measure of interest pre- versus post-intervention assessment. For example, in the following model:

$$E[Y_{ijk}] = \alpha_i + \beta_0 + \beta_{1j}\,\text{Post-intervention} + \beta_{ik} X_{ik}$$

… where Y_{ijk} represents the secondary outcome measure of interest for the *i*th treated subject, *j*th assessment (1=post-intervention, 0=pre-intervention), with covariate distribution $X_{k,}$ where X_k could represent age, sex, education in addition to other potential secondary outcome measures. Based on this model, β_1 would represent the mean change in the measure of interest (e.g., PHQ-9) post-intervention vs. pre-intervention, accounting for differences in age, sex, education status, and other measures between participants. Similarly to the previous model, a random intercept would be included to account for within-subject correlation. Additional parameters (e.g., random slope) could be added to the model for each subject to examine between-subject variability not explained by other model parameters.

In the case that the different secondary outcome measures are not normally distributed, non-parametric methods and/or modeling strategies (e.g. generalized linear models) will be applied, in the context of repeated measures, to evaluate the effects of interest.

Interim Analyses

Interim analyses will be conducted with n=100 and n=150 participants, respectively, who completed the protocol. To maintain a type 1 error = 0.05 and power = 0.9, based on an analysis of the entire sample (n=200), larger critical values were calculated for the first and second interim analyses required for rejecting the null hypothesis (i.e., no mean difference in ISI score between active and control groups). These larger critical values correspond with significance tests (p-values) of 0.004 and 0.0196 at the first and second analysis, respectively. The significance test required for the third (and final) analysis, based on the full sample, is slightly lower ($p < 0.045$) as result of including the interim analyses. Stopping boundaries were based on methods developed for sequential design and provide critical values at different stages that would approximate α=0.05 given an analysis of the full sample (O'Brien and Fleming, 1979). Analyses that indicate significant difference in insomnia severity, depression, plus PTSD scores (scores for ISI, PHQ-9, and PCL-5, respectively) jointly, between pre- and post-intervention assessments, based on these reduced p-values, will result in early termination of the trial due to intervention efficacy. To

assess group differences with respect to the joint distribution of these measures, we will employ multivariate analysis of variance (MANOVA). Changes in scores for the respective outcome measures will be examined as dependent variables with respect to group assignment. An overall test, as well as individual tests of difference, of the dependent measures will be assessed and a decision will be made to terminate the study. Only the statistician will have access to the interim analysis results while the rest of the investigators will remain blinded.

Missing Data

It should be noted that in order to advance through the different eCBT-I training (online study portal) modules, participants need to complete questions related to the primary and some secondary outcome measures (i.e., ISI, fatigue, daytime sleepiness). Therefore, data will not be missing intermittently during the trial period (i.e., 1 week - 9 week). However, participants may decide to drop out of the study before completion of the entire protocol which could result in right censored or missing data beyond the time point at which participant leaves the trial. Different analytical strategies will be applied. If the data are missing at random between the active and control groups, linear mixed-effects models will be utilized which essentially impute the missing values with mean data of participants who completed the protocol (Peters et al., 2012; Bell et al., 2013). If data are not missing at random, such as informative censoring, the distribution of missing data will be examined with respect to available prognostic indicators in control and active groups at baseline and over follow-up. Analytical strategies such as inverse-probability-of-censoring weighted methods will be utilized which “upweight” data of s participants who have completed the protocol who share characteristics of those who dropped out of the study as a way to adjust estimates for dropout (Robins et al., 2000). Other analytical tools will be used and results will be compared to determine and correct for potential bias in effect estimates (Bell et al., 2013; Pericleous, 2016).

Exploratory analyses

In addition to examination of primary and secondary outcome measures with respect to intervention, analyses will be conducted utilizing data based on secondary measures (i.e., sleep diaries, etc.) and examined with respect to the primary (i.e. ISI) and secondary outcome measures (i.e., PCL-5, PHQ-9). These secondary measures represent compliance measures (i.e., measure of participant willingness to record and track sleep patterns based on protocol instructions consistent with good sleep practices). Analyses will examine compliance (i.e., missing secondary measures versus non-missing secondary measures) with respect to outcome measures and statistical tests will be performed (i.e., two-tailed t-test). We hypothesize that participants who record daily sleep diaries throughout the intervention period will have more significant benefits in terms of ISI change than participants who record sleep diaries intermittently.

Power Analyses

Different power analyses were applied to evaluate whether potential effects of interest would be significantly based on the different analyses above. Previous reports that have examined ISI with respect to eCBT-I intervention and have found significant effects (Ritterband et al., 2009; Ritterband et al., 2017). Given reported distributions from these reports, data were simulated and power to detect given effects was evaluated based on different conditions including: (1) sample size (pre-specified for N=200, but investigated for lower N); (2) arm imbalance (3:1 vs 1:1); (3) variability of the outcome measures under evaluation; (4) study attrition; and (5) adjustment for covariates with respect to analyses of secondary outcomes.

Power curves were generated in the case of the first analysis described above to examine AUC based on hypothetical intervention-response curves. AUC was varied relative to AUC = 0.5 (i.e., no intervention effect). Differences in sample size were compared in the different curves to reflect potential dropout rates of 0%, 20% and 40% in the study (Figure 8).

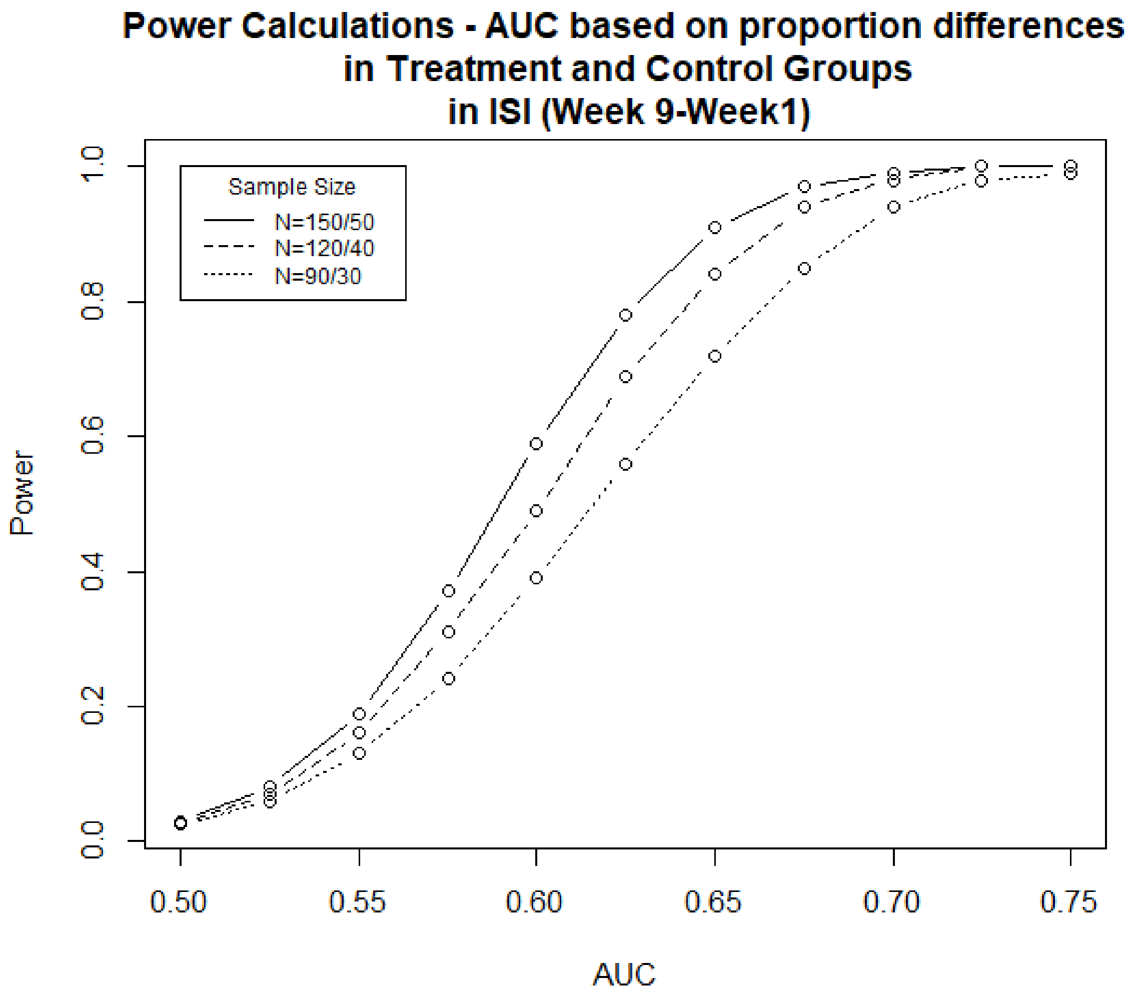

Figure 8. Calculated power of AUC analysis as affected by varying estimated sample size and participant attrition rates.

In addition to the power curves generated for AUC, power estimates were generated based on a complementary set of analyses to investigate the effect of intervention on difference in ISI score (9 week – 1 week), using instead a repeated-measures analysis of variance (ANOVA) to model the effects of interest – i.e., Y~Intervention + Time + Intervention x Time. Power was evaluated with respect to the Intervention x Time effect from this model – i.e., the parameter that would indicate an effect of intervention on ISI score differences (9 week-1 week), based on a two-sided test and α=0.05. In this analysis, different conditions were modified to evaluate their respective effects on power. Differences in group means in ISI score were simulated to be close to zero at week 1, while effect size reflects differences in these group means at week 9 (note: Ritterband et al. (2017) reported mean differences of ~ 0 (SD = 5.5) and 5 (SD=5.5) at pre/post-assessment).

Arm Balance	Mean Difference	Stddev	Total N	Power
3:1	5	5.5	200	1
3:1	5	5.5	160	1
3:1	5	5.5	120	1
3:1	5	8.5	200	0.99
3:1	5	8.5	160	0.96

3:1	5	8.5	120	0.90
3:1	3	5.5	200	0.95
3:1	3	5.5	160	0.89
3:1	3	5.5	120	0.79
3:1	3	8.5	200	0.64
3:1	3	8.5	160	0.54
3:1	3	8.5	120	0.43
1:1	5	8.5	200	0.99
1:1	5	8.5	160	0.99
1:1	5	8.5	120	0.96

Table 1. Power estimates of intervention effects pre- and post-intervention (1 week-9 weeks) under different conditions.

This calculation assumes correlation structure among residuals of repeated-measures ANOVA. $\rho_{jj'} = \rho^{c}$ where ρ=0.7 and $c=|j-j'|^{\theta}$ =0.8 (i.e., ρ represents correlation between adjacent weeks (week 1 versus week 2) and c represents the decay in correlation at additional subsequent weeks (week 1 versus week 3, …, week 9).

Based on Table 1 and assuming results of the current study are comparable to previous intervention studies involving CBT-I and ISI, differences in protocol in the current study with respect to arm balance and N should have negligible effects for evaluation of intervention effects with respect to ISI score between 1 week and 9 weeks.

Power curves were also generated to examine differences in intervention effects with respect to changes in ISI between 1 week and 3 months. The plots below depict different power estimates based on the effect size of the Intervention x Time interaction in the models described previously. Differences in power were examined assuming differences in the standard deviation of the ISI measure at the 3 month assessment and assuming a dropout rate of 20% and 40% at that time point (Figures 9-10).

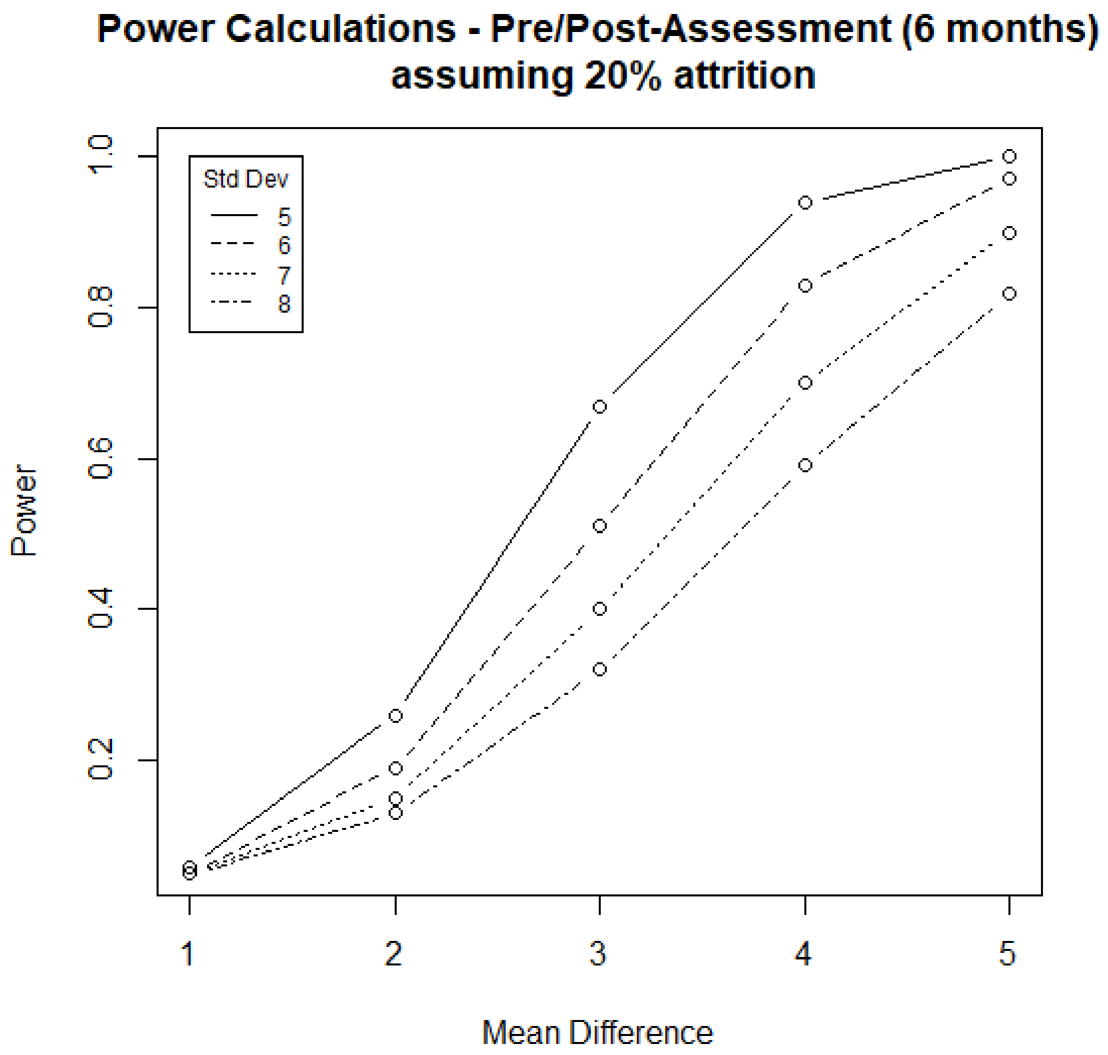

Figure 9. Calculated power of mean difference analysis between pre- versus post-intervention assessments assuming 20% attrition rate.

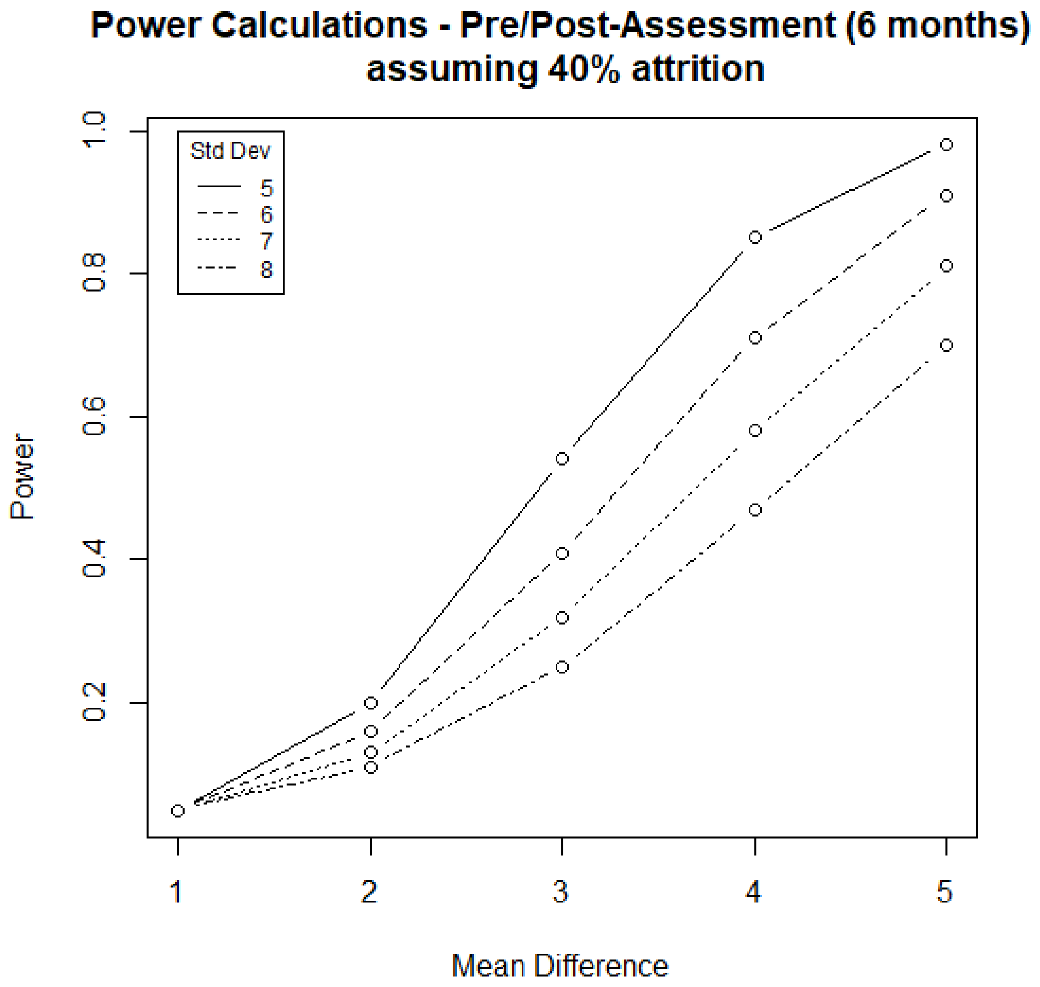

Figure 10. Calculated power of mean difference analysis between pre- versus post-intervention assessments assuming 40% attrition rate.

Ritterband et al. (2017) reported significant mean differences between active and control groups of ~ 4 at the 3 month follow-up assessment, a standard deviation of 5.6 in ISI score (at month 6) in each group (N =114 eCBT-I and 129 control), respectively. Based on Figures 9 and 10 above, there would be sufficient power to detect similar effects based on a smaller sample assuming 20% attrition (i.e., group N = 120 and 40) and 40% attrition (i.e., group N=90 and 30) at 3 month follow-up. Based on this and previous study data, the study investigators predict smaller rates of attrition associated with a 3 month follow-up as part of this investigation.

Lastly, power curves were generated also to examine detection of secondary outcome measures, specifically changes in these measures, in the treated group that would hypothetically follow from eCBT-I intervention for insomnia. For the secondary measures, we utilized surrogate measures based on the Posttraumatic Stress Disorder Checklist Military Version (PCL-M) and Beck Depression Inventory II (BDI-II), which have been examined with insomnia in previous reports. Briefly, these reports examined prevalence of ISI and its correlation with measures of PTSD and depression in both pre-deployed service members (Taylor et al., 2016) and deployed service members who had sustained repeated TBI (Bryan, 2013). This literature has reported differences in PTSD and depression in those with clinical insomnia (defined by the authors as ISI > 15) versus those with no insomnia (ISI < 15) (Bryan, 2013; Taylor, 2016). Although these represent surrogate measures for the variables planned for the study (i.e., PCL-5

and PHQ-9), differences with respect to the power analysis should be negligible and the results should provide a reasonable representation of those based on the actual study measures.

Based on the marginal and joint distributions (i.e., correlations) of the measures from these studies, multivariate normal data for ISI, PTSD, and depression were simulated pre- and post-assessment (e.g. week 1 – week 9) in 150 participants (i.e., number of participants in the treated group for whom changes in secondary measures would be examined). Changes in ISI score in treated participants were generated based on previous reports (e.g. week 1 – week 9) (Ritterband et al., 2017). Correlations between ISI, PTSD and depression from the literature were assumed to remain constant both pre- and post-assessment. Power to detect mean differences in PTSD > 12 (PCL-M scale) or depression > 7 (BDI-II scale), for each measure separately, that correspond with reduction in insomnia severity, using a paired two-sided t-test with α=0.05, is large (e.g. power > 0.99).

Power curves were generated also to examine detection of change in PTSD where depression was included as a covariate (for example, as a covariate in a linear model as shown previously) (Figure 11), and change in depression where PTSD was included as a covariate (Figure 12). Power estimates are based on a paired two-sided t-test with α=0.05, where effects relate to the extent PTSD and depression are correlated (i.e., a smaller mean difference effect size indicates stronger correlation between the measures).

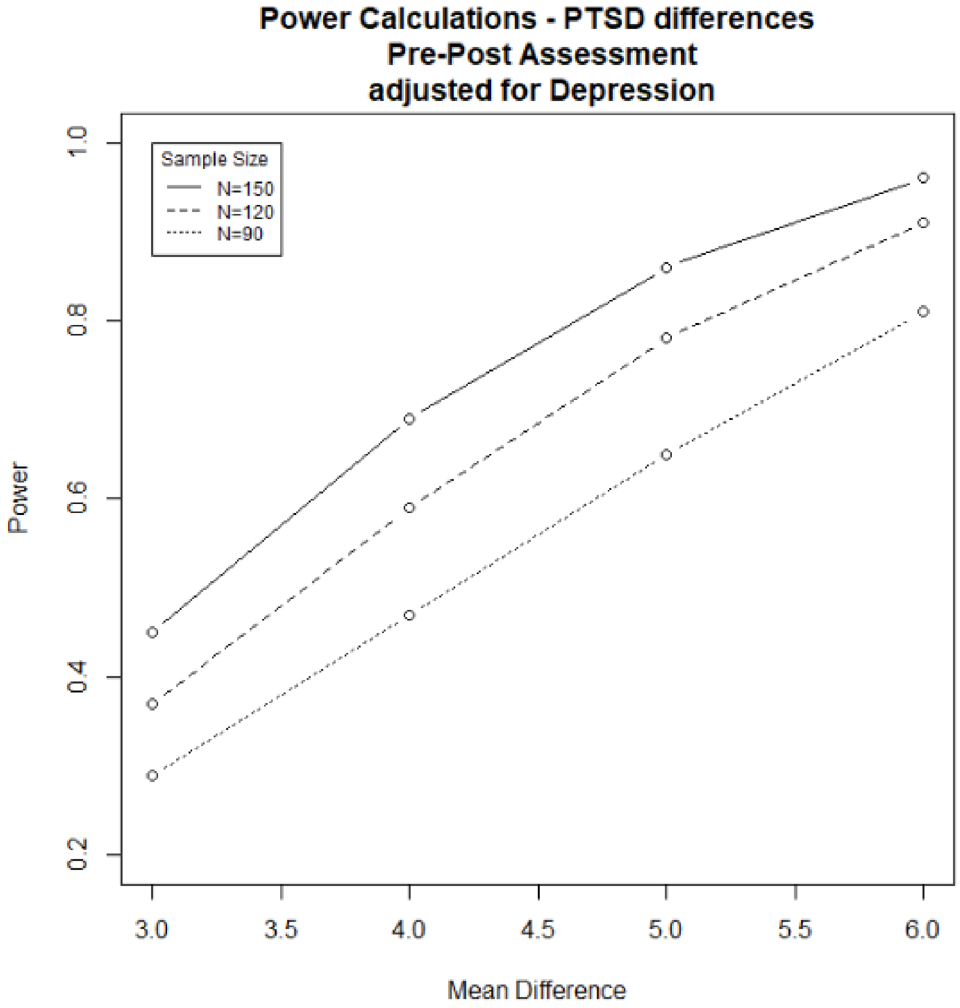

Figure 11. Power curve for detection of change in PTSD with depression as a covariate.

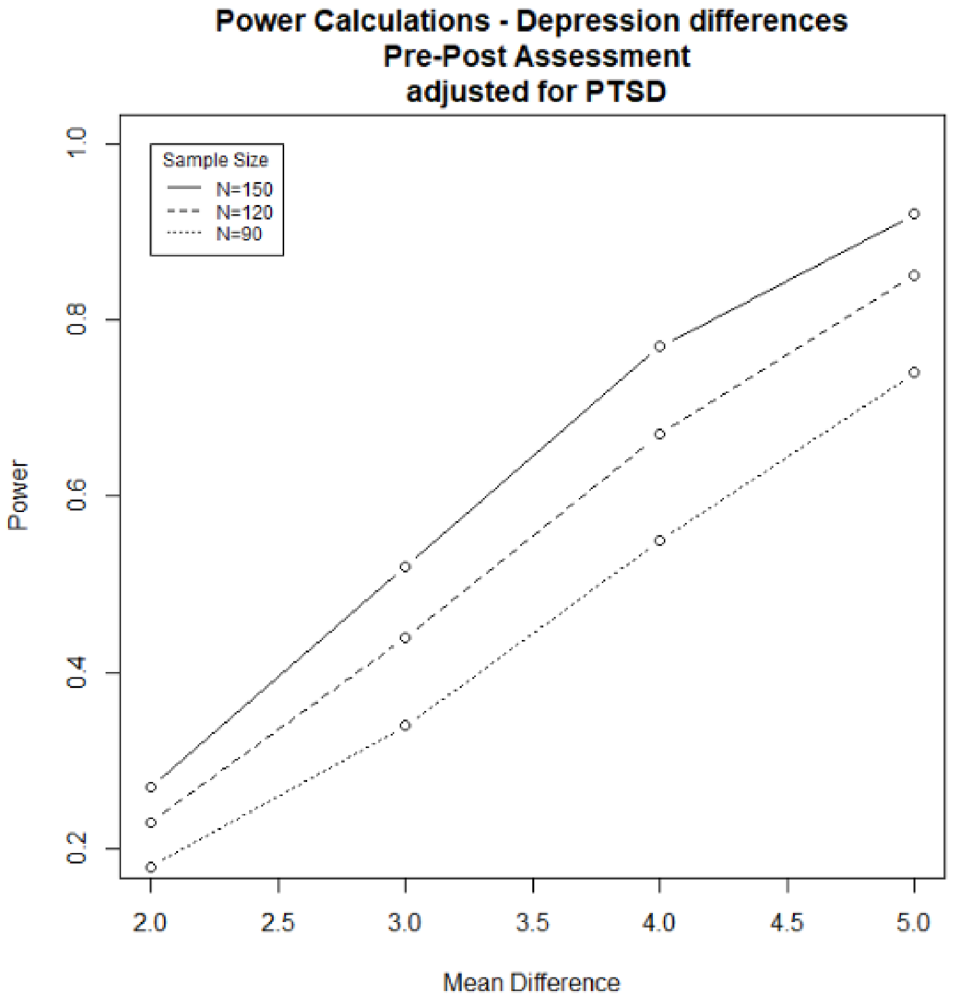

Figure 12. Power curve for detection of change in depression with PTSD as a covariate.

Power analyses were conducted using SAS version 9.4 and R version 3.4.4.

Ongoing Safety Analysis

This clinical trial is designed to be minimal risk. ECBT-I has been documented as a highly safe and efficacious intervention for insomnia in a general population. This investigation provides a means for helping active and retired service members that may otherwise not seek assistance due to lack of access to qualified practitioners, unwillingness to physically travel to healthcare facilities, or perceived stigmatization associated with receiving a psychologically-focused therapy. However, if AEs or SAEs are reported by participants during interactions with the study team, they will be immediately referred for appropriate intervention at their nearest Military Treatment Facility (MTF) and the findings reported to the IRB. The study team will inquire as to the presence of AEs and/or SAEs during the already scheduled interactions with study participants. Furthermore, if during the course of the study a statistically significant number of AEs or SAEs are reported, the study team will perform a partial early database lock and execute an unblinded safety analysis or halt study activities entirely until an appropriate corrective action plan can be implemented.

PARTICIPANT INFORMATION

Subject Population:

Male and female active or retired service members between the ages 18 and 64 with insomnia and history of TBI.

Inclusion Criteria:

1. Age 18-64
2. Active or retired service members with DEERS eligibility
3. Ability to provide verbal and electronic informed consent and follow study-related instructions
4. Presence of clinical insomnia for a period of at least 1 month prior to consent as confirmed by self-reported ISI (score >=15) and PSQI (score >= 5)
5. History of TBI >= 6 months prior to consent, including blast-related, as confirmed by a telephone-administered TBI Screener
6. Reliable access to a telephone and the Internet via their computer or smartphone
7. Stable regimen of medications for sleep or potentially affecting sleep over prior 1 month as confirmed by clinical history review.

Exclusion Criteria:

1. Current or previous CBT-I or eCBT-I intervention; participants may still receive other approved therapies provided standard of care
2. Life expectancy of less than 6 months
3. Rapidly progressive illnesses such as late stage cancer, neurodegenerative conditions, major organ failure, etc.
4. History of moderate to severe substance use disorders with the exception of nicotine
5. Active bipolar disorder or psychosis that could be worsened by mild sleep restriction as part of eCBT-I.
6. Routine irregular work schedules or sleep patterns defined as shift work greater than 1 day per week
7. Discontinuation of DEERS eligibility resulting in immediate subject withdrawal.

RECRUITMENT AND CONSENT

Identification and Selection of Participants:

Potential participants will be referred to the study team through multiple channels so as to maximize potential therapeutic benefit for the highest number of interested persons. At no point during the study will potential participant names or contact information be directly transmitted to non-approved recipients or third-parties. Recruitment materials will be uniform across all channels and locations. All recruitment materials will be IRB-approved by USU and institutionally approved by local oversight committees as necessary. Interested persons will initiate contact the with study team using information provided via one of the following advertisement channels:

CNRM Recruitment Core

The study will utilize the CNRM Recruitment Core to assist in the recruitment of research participants. The core coordinates an open-referral recruitment protocol for all persons interested in learning more about clinical research opportunities in TBI. Participants are pointed to a web-based consent and questionnaire describing the overall scope of CNRM research efforts and provide contact information requesting further information. Persons are screened based on self-report for basic health information and exclusion criteria (i.e., age, presence of traumatic brain injury, affiliation with the MHS). Screening information is then channeled to study investigators matching them with eligible referrals for specific clinical investigations.

DVBIC

Providers in the Defense and Veterans Brain Injury Center (DVBIC) TBI Recovery Support Program will make available contact details, flyers, cards, and posters providing basic information should patients express interest in learning more about the study. Regional Education Coordinators may also be offered recruitment materials for distribution at events.

MTFs

Providers at MTFs will make available contact details, flyers, cards, and posters providing basic information should patients express interest in learning more about the study. Regional Education Coordinators may also be offered recruitment materials for distribution at events. These MTFs may include Walter Reed National Military Medical Center, Camp Pendleton, Fort Bragg, Joint Base San Antonio, Camp Lejeune, Fort Campbell, Fort Carson, Fort Hood, Fort Bliss, and Fort Belvoir.

Media

Study personnel will publicize the study through various forms of media including but not limited to: MTF bulletin boards, social media, or other Internet-based advertisements. Prior to initiating recruitment with such media outlets, approval from the respective outlets will be established. Study personnel may contact coordinators of relevant organizations (e.g., Wounded Warrior Project, BrainLine) and also ask for a post on their social media sites (e.g., Facebook, Twitter). The study will also use institution intramural and extramural websites, including ClinicalTrials.gov, and DoD/VA/USUHS centers.

Recruitment Process:

Once a potential participant has contacted the research team, they will be contacted by a study investigator to gauge their interest in research. Interested participants will be sent an initial message (via email or telephone/voicemail) to acknowledge receipt of their contact information. After the initial message, three subsequent contact attempts will be made by the study investigators to schedule and consent potential participants. Once contact is initiated, they will be invited to participate and will be provided with the standard consent procedures including the background of the study, study procedures, alternatives to participation, and potential risks and benefits. Potential participants will then be screened by study investigators according to the inclusion-exclusion criteria and a two-factor verification of identity.

Compensation for Participation:

Monetary compensation is not being offered as a part of this study.

Eligibility Assessment Process:

Screening for eligibility based on inclusion-exclusion criteria will be performed over the phone and through AHLTA with all potential participants after consent has been provided. Screening procedures can happen during multiple sessions via telephone so as to accommodate participant availability. Study investigators will provide a detailed explanation of the study along with the informed consent and HIPAA form for review prior to verbal and electronic consenting. Participants will be provided with an opportunity to ask any questions they may have regarding the study before providing consent. We plan to enroll up to 200 participants in the study to ensure estimations of statistical power and attrition are adequately met.

A phone consultation for eligibility assessment will be scheduled with the participant after obtaining informed consent. During the telephone discussion, the study team will collect medical history relevant to the study inclusion-exclusion criteria. The study team will also perform a two-factor verification of identity and DEERS eligibility using AHLTA and a provided SSN. In order to do this, the study team will first locate the participant's electronic medical record within MHS. Once DEERS eligibility is confirmed as indicated by the electronic system, investigators will verify identity by requesting the patient verbally

confirms one or more specific elements of their electronic medical record. For example, the study investigator may request the participant provides the name of their designated case manager, most recent visit date to their local MTF, or similar question, and confirm that the provided answer accurately corresponds with official record.

Persons who do not meet eligibility criteria or fail two-factor identification will be excluded from participation and documented as screening failures. Persons who are ineligible for participation will be educated on alternative therapeutic options and/or provided contact information for a sleep specialist clinic local to them.

Consent Process:
Are you requesting a waiver or alteration of informed consent?

○ Yes ● No

Please explain why you qualify for a waiver or alteration of informed consent:
N/A

Please explain the consent process:
Potential participants will be provided with an IRB-approved electronic informed consent, institutionally approved by the Department of Research Programs, USU, during their initial telephone consultation prior to the start of research activities. Persons expressing interest in participating will complete the informed consent process via a telephone and web-based process described below. For each participant, every effort will be made to ensure breadth of subject understanding of study expectations, voluntariness to participate, alternative intervention options, and adherence to safety precautions. The study team will also explain to potential participants that their involvement is strictly for research purposes only, will not become part of or influence their electronic health record within MHS, and will have no repercussions regarding the standard care they receive elsewhere or their military duty status. The work for this study is purely exploratory in scope and has no legal influence on decisions such as disability assessment or health care benefits.

Potential participants may only complete the consenting process via the established telephone and web-based procedure. After the study team is contacted by the potential participant informing them of their initial interest, they will then undergo a consultation via telephone provided by the study team. During the telephone consultation, the study investigator will provide the patient with information directing them to the study-designated online consent portal. Once the participant verifies the document is open, the study investigator will review study procedures, voluntariness and alternatives to participation, the consent form, and HIPAA authorization. The study team will provide sufficient time to answer any questions before consenting. There will be no specific time limit between participant contact and informed consent; potential participants can ask questions and consider participating as long as they want while the study is open to enrollment.

Once the potential participant verbally agrees to be part of the study, they will be asked to digitally check a box in the consent portal while providing their name with date and time, verifying willingness to participate in the research study. As part of the consenting process, the participant will be required to provide simultaneous telephone and digital confirmation of consent noted by their name, date, and time.

Withdrawal from Study Participation:
Participants have the right to withdraw from participation in the study at any point without the need to provide a reason for withdrawal. Study investigators may also withdraw participants from the study for medical, administrative, or non-compliance concerns. Participants wishing to be withdrawn

from the study must do so with a written request to the PI. Coded data collected up until the point of withdrawal may be used in data analysis.

RISKS AND BENEFITS

Risks of Harm:

General

There are risks, discomforts, and inconveniences associated with any research study. For participants randomized to the education control group, there is the possibility that their insomnia symptoms may worsen during the period of non-active participation. Participants will be advised to continue their current standard of care throughout participation in the study including use of medications as prescribed by their primary physician. Participants in the education control group will be offered free open-label intervention following their final 3 month follow-up evaluation so as to provide the potential of therapeutic benefit for all persons regardless of group randomization assignment.

Outcome Assessments

Some of the questions asked during outcome assessments are sensitive in nature. Answering these assessments may cause participants psychological distress. Participants will be advised they are free to stop the outcome assessments and study participation at any time. Participants that present a risk of harm to themselves or others will initiate immediate activation of the Emergency Action Plan. For more information on the Emergency Action Plan, see section titled ‘Measures to Minimize Risks of Harm (Precautions, Safeguards)’.

Research Records

All private study information, PHI, and PII, will be kept in a locked file cabinet behind locked door or on an appropriately secured data server with access limited to approved study team members in order to minimize risk. Participants will be given a coded study ID that will be used on all data forms following successful enrollment, including information being transmitted electronically through AWS and Qualtrics (as administered the investigators and collaborators at UVA). For additional information on the safeguarding of protected information, see sections titled ‘Data Management’ and ‘Managing Data (Data Management and/or Sharing Plan) for this Study’.

ECBT-I/CBT-I

As Internet-guided delivery of CBT-I is designed to be mimetic of traditional in-person therapy in terms of technique and efficacy, so too are the potential for risks and benefits identical with the exception of potential electronic information breach generally associated with use of the Internet or similar telemedicine-based technologies.

Sleep restriction, which imposes a strictly controlled sleep duration and sleep-wake schedule during intervention, is hypothesized to be the most influential determinant in intervention success (Morin et al., 2006; Spielman et al., 1987). The effects of mild sleep deprivation for participants may therefore include feeling tired, fatigued, or anxious for a period of several weeks.

Measures to Minimize Risks of Harm (Precautions, Safeguards):

Emergency Action Plan

If a study team member has indication suggesting a participant may be in danger, either to themselves or others, a triage-based emergency action plan consisting of (1) urgent response, (2) direct referral, and (3) outside referral, will be initiated to ensure comprehensive and rapid follow up. Prioritization of action will be based on level of risk perceived by the study team.

All situations requiring initiation of the emergency action plan will reported to IRB and the CNRM within 48 hours. As appropriate and at the recommendation IRB, the participant will be directed

to local resources. If judged necessary by IRB in order to protect the welfare of the participant or another person, confidentiality may be breached as part of the referral process. The participant may also be advised or removed from participation in the study if there is evidence of safety concern to the participant or others. The study team is not obligated to report results to military or civilian authorities unless there is evidence of imminent danger to the participant or others.

(1) Urgent Response

In event of immediate threat of or confirmed harm to themselves or others, the study team will contact 911 or the appropriate local emergency response authority. As part of the enrollment and medical history review process, emergency contact info, physical address, and name of primary care provider are collected by the study team. This information may be provided to emergency responders to coordinate an immediate response on the participant’s behalf. Urgent response actions may be initiated in the event of threat of suicide, imminent violence, or similarly dire circumstances necessitating immediate intervention.

(2) Direct Referral

In scenarios with perceived but less-than-urgent risk, the PI and study team may direct participants to local resources where appropriate. This may include referral to their local MTF, VA, or non-military relevant care provider. Direct referrals may be initiated on if significant cause for concern is indicated. The PI and study team will perform necessary correspondence with the participant and outside referral or provider(s) so as to ensure appropriate follow-through. Potential participants failing to meet inclusion-exclusion criteria may also be subject to this guidance in the event that cause for concern is discovered as part of their screening procedures.

(3) Outside Referral

If a participant declines direct referral or other support attempts offered by the study team, they will be referred to an external public hotline service capable of providing 24/7/365 emergency consultation. The Veterans Crisis Line (www.veteranscrisisline.net), formerly known as the National Suicide Prevention Lifeline, is a well-established public suicide prevention organization founded in 2007 that is funded and operated by the VA and US Department of Health and Human Services (DHHS). Since its inception, the Veterans Crisis Line has answered more than 3.3 million calls and initiated more than 93,000 emergency dispatches (US Department of Health and Human Services, 2018). The organization also offers support services via online chat and text messaging. All services are confidential and voluntary.

Safety Monitoring Plan

Monitoring is a major component of research support to ensure research participant safety, verify accurate data collection, identify problem areas, and take corrective action to resolve problem areas when necessary. The monitoring process includes verifying all enrolled participants have undergone necessary protocol eligibility and regulatory compliance according to the International Conference on Harmonization, Good Clinical Practice, DoD, NIH, DHHS, and FDA guidelines as applicable. Descriptions of preparation, performance, and follow-up for monitoring visits are also described below.

The study team, in conjunction regulatory monitoring representatives at the CNRM, will exercise routine vigilance through examination of the assessments, measures, and other electronic records. The independent regulatory monitor at the CNRM will contact the PI and study team with information to review in preparation for a monitoring visit in accordance with the established schedule outlined in the monitoring plan.

During the visit, the regulatory monitor will compare the medical records and research files to the protocol documents and submitted forms to verify compliance and accurate data collection. The

regulatory monitor will follow standardized procedures according to the established CNRM monitoring plan.

Safety Analysis Plan

Confidentiality Protections (for research records and data):

See sections titled ‘Data Management’ and ‘Managing Data (Data Management and/or Sharing Plan) for this Study’.

Potential Benefits:

Participants may benefit from participation in this study in terms of a reduction in clinical insomnia symptoms due to eCBT-I intervention. However, this benefit cannot be guaranteed as no psychological therapies are universally effective. This study is likely to yield important information about the feasibility and efficacy of eCBT-I intervention in regards to insomnia with history of TBI that could inform future clinical guidance. Participants will also receive additional clinical assessments not part of their standard care that can be shared with their primary physician or mental health provider if requested by the participant.

Privacy for Participants:

Records of participant participation in this research study may only be disclosed in accordance with state and federal law, including the Federal Privacy Act, 5 U.S.C.552a, and its implementing regulations. DD Form 2005, Privacy Act Statement - Military Health Records, contains the Privacy Act Statement for the records.

Procedures will be taken to protect the confidentiality and privacy of study participants. Upon providing verbal and written electronic informed consent, participants will be assigned a coded study ID. The coded study ID will be used to mask personal information such as name and other identifiable information in research records. A Master List linking the real names of participants with coded study IDs will be kept in a locked office and file cabinet or in an electronic database located behind a secure firewall. Only approved study personnel will have access to information that could be used to distinguish or trace an individual’s identity. Study collaborators and vendors at the University of Virginia, Qualtrics, and Amazon Web Services will not have access to identifiable participant information.

Data collected during this study will be shared with CNRM. This data will not contain any identifiable information. Representatives of CNRM, USU, the Henry M. Jackson Foundation (HJF), and NIH may have access to study data for audit purposes. We may share unidentifiable data with outside investigators or collaborators. This data may be used for a variety of research purposes that we may not be able to specify at this time.

Researchers will make every effort to protect participant privacy and confidentiality; however, there are always risks of breach of information security and information loss. Complete confidentiality cannot be promised for military personnel, because information regarding participants may be required to be reported to appropriate medical or command authorities to ensure the proper execution of the military mission, including evaluation of fitness for duty.

Incidental or Unexpected Findings:

It is possible that during execution of the protocol we will encounter participants with potential imminent danger or findings that ethically require immediate action. This information may be revealed during the course of the initial screening interview or at subsequent follow up assessments during the period of the study. If a research staff member has any information indicating that the participant may be

in danger, dangerous to themselves, or dangerous to others, the Safety Monitoring Plan will be initiated. The incidental or unexpected findings will be reported to IRB within 48 hours. As appropriate and at the recommendation of the PI and/or the IRB, the participant will be directed to local resources. If judged necessary by IRB in order to protect the welfare of the participant or another person, confidentiality may be breached as part of the referral process. The participant may also be advised or removed from participation in the study if there is evidence of safety concern to the participant or others. The study team is not obligated to report results to military or civilian authorities unless there is evidence of imminent danger to the participant or others.

STUDY MONITORING

Data Monitoring Plan:

Data monitoring will be coordinated by the study data manager. The data manager will supervise data collection ensuring accuracy and participant protection on an ongoing basis. The data manager will review all data collection forms on an ongoing basis, to include: (1) quality assurance of assessments and forms for completeness, (2) quality assurance of electronically entered data, (3) integrity checks of coded research records determining variables are within expected ranges, (4) quality assurance of documents for proper identifier redaction, and (4) protection of databases through the appropriate use of secure networks and password protection. The PI will ensure that all analyses are completed and disseminated.

For additional information regarding data security, see sections titled ‘Data Management’ and ‘Managing Data (Data Management and/or Sharing Plan) for this Study’.

Safety Monitoring Plan:

See section titled ‘Measures to Minimize Risks of Harm (Precautions, Safeguards)’.

REPORTABLE EVENTS

Reportable Events:

Adverse Events (Expected)

Expected adverse events which are not serious will be reported on the Continuing Review Progress Report. More frequent Progress Reports may be provided based on the discretion of the IRB. A summary of adverse events study-wide will be included as part of the Continuing Review Progress Report.

Adverse Events (Unexpected)

Unexpected, but not serious, adverse events occurring in participants which, in the opinion of the PI, are possibly related to participation in the protocol will be reported by the PI within 10 working days to the IRB and the CNRM using the same procedure. Unexpected, but not serious, adverse events occurring in participants which, in the opinion of the PI, are possibly related to participation AND places participants or others at a greater risk of harm that was previously known or recognized in the protocol will be reported by the PI within 24 hours of delivery by email or phone to the IRB. Additionally, a follow-up written report within 10 business days to the IRB will be completed.

Serious Adverse Events

The PI, within 24 hours, will report all serious adverse events (SAE). This is accomplished by submitting an adverse event report to the IRB. All serious adverse events will also be reported to CNRM within 24 hours of site notification.

Unanticipated problems

Unanticipated problems involving risks to subjects or others (UPIRTSOs) will be reported to the IRB and CNRM and via email or telephone within 24 hours of discovery and a written follow-up report within 10 business days.

Protocol Deviations

When a deviation occurs, the investigator will report the occurrence to the IRB. The investigator will make the determination whether the deviation meets the criteria for an UPIRTSO. Deviations that are determined to be minor will be reported on the CR Progress Report.

Additional information regarding potential risk of adverse events, safety measures, and action plans is provided in the section entitled "Risks and Benefits."

EQUIPMENT/NON-FDA REGULATED DEVICES

Does the study involve the use of any unique non-medical devices/equipment?

○ Yes ◉ No

Please describe:

FDA-REGULATED PRODUCTS

Will any drugs, dietary supplements, biologics, or devices be utilized in this study?

Yes

Reporting Requirements for FDA-regulated research under IND and IDE:

Active eCBT-I intervention and education control portals will be operated through Sleep Healthy Using the Internet (SHUTi). SHUTi is a minimal risk medical device intended to decrease symptoms of insomnia through the use of self-guided lifestyle intervention techniques. Although numerous blinded clinical trials have demonstrated a strong track record in terms of efficacy and safety, SHUTi has not been officially evaluated by FDA.

An abbreviated IDE is proposed for this investigation whereby the study team has confirmed the necessary regulatory standards. A full IDE application and interaction with FDA is therefore not required. According to 21 CFR 812.2, section (b), all required criteria for the pathway are considered herein:

1. The medical device is not currently banned per FDA.
2. The device is labeled in accordance with 21 CFR 812.2, Investigational Labeling Standards.
3. The study team will obtain IRB approval for the proposed investigational plan and has presented data to support that the device is not a significant risk device.
4. Each participating investigator will obtain from each participating subject verification of documented informed consent under 21 CFR 50. A formal waiver of documented consent may also be approved by IRB under appropriate circumstances in studies involving minimal risk to participants.
5. Compliance with monitoring requirements for clinical investigations per 21 CFR 812.46.
6. The study team maintains record keeping under standards listed in 21 CFR 812.140(b), parts 4 and 5, and makes reports to IRB under 21 CFR 812.150(b), parts 1 through 3 and 5 through 10.
7. The study team maintains record keeping under standards listed in 21 CFR 812.140(a), part 3(i), and makes the reports required under 21 CFR 812.150(a), parts 1, 2, 5, and 7.
8. The study team will comply with prohibitions listed in 21 CFR 812.7 against promotion and other related practices.

Sponsor (organization/institution/company):

This study is sponsored by the CNRM, a congressionally-funded affiliate organization of USU, and is receiving no funding from industry or other private sources. The CNRM was established as a collaborative intramural program in May 2008. As part of the CNRM mission, this study will optimize the scientific advantages of comparing military and civilian cohorts, and position the CNRM to transition advances in the field of TBI research from civilian studies to military populations.

REFERENCES

1. Ancoli-Israel S, Cole R, Alessi C, Chambers M, Moorcroft W, Pollak CP. 2003. The Role of of Actigraphy in the Study of Sleep and Circadian Rythms. Sleep 26: 342-92.
2. Ancoli-Israel S, Roth T. 1999. Characteristics of insomnia in the United States: results of the 1991 National Sleep Foundation Survey. I Sleep 22(Suppl 2): S347–53.
3. Bagalman E. 2015. Health Care for Veterans: Traumatic Brain Injury. Congressional Research Service. Access 7 Mar 2018
4. Bastien CH, Vallieres A, Morin CM. 2001. Validation of the Insomnia Severity Index as an outcome measure for insomnia research. Sleep Med 2(4): 297-307.12.
5. Bell ML, Kenward MG, Fairclough DL, Horton NJ. 2013. Differential dropout and bias in randomised controlled trials: when it matters and when it may not. BMJ Jan 21;346:e8668.
6. Benca RM, Peterson MJ. 2008. Insomnia and depression. Sleep Med 9(1):S3-S9.
7. Blackwell T, Redline S, Ancoli-Israel S et al. 2008. Comparison of Sleep Parameters from Actigraphy and Polysomnography in Older Women: The SOF Study. Sleep 31(2): 283-291.
8. Blevins CA, Weathers FW, Davis MT, Witte TK, Domino JL. 2015. The Posttraumatic Stress Disorder Checklist for DSM-5 (PCL-5): Development and initial psychometric evaluation. Journal of Traumatic Stress, 28, 489-498.
9. Buscemi N, Vandermeer B, Friesen C, Bialy L, Tubman M, Ospina M, Klassen TP, Witmans M. 2005. Manifestations and Management of Chronic Insomnia in Adults. Evidence Report/ Technology Assessment No. 125. AHRQ Publication No. 05-E021-2. Rockville: Agency for Healthcare Research and Quality.
10. Butt Z, Lai JS, Rao D, Heinemann AW, Bill A, Cella D. 2013. Measurement of fatigue in cancer, stroke, and HIV using the Functional Assessment of Chronic Illness Therapy - Fatigue (FACIT-F) scale. J Psychosom Res 74(1): 64-8.
11. Buysse DJ, Germain A, Moul DE, et al. 2011. Efficacy of brief behavioral treatment for chronic insomnia in older adults. Arch Intern Med. 171(10): 887-95.
12. Buysse DJ, Reynolds, CF III, Monk TH, Berman SR, Kupfer DJ. 1989. The Pittsburgh sleep quality index: A new instrument for psychiatric practice and research. Psychiat Res 28(2): 193-213.
13. Bryan CJ. 2013. Repetitive Traumatic Brain Injury (or Concussion) Increases Severity of Sleep Disturbance among Deployed Military Personnel. Sleep 36(6): 941-946.
14. Caldwell JA, Knapik JJ, Lieberman HR. 2017. Trends and factors associated with insomnia and sleep apnea in all United States military service members from 2005 to 2014. J Sleep Res 26(5): 665-670.
15. Carpenter JS, Andrykowski MA. 1998. Psychometric evaluation of the Pittsburgh Sleep Quality Index. J Psychosom Res 45(1): 5-13.
16. Christensen H, Batterham PJ, Gosling JA, Ritterband LM, Griffiths KM, Thorndike FP, Glozier N, O'Dea B, Hickie IB, Mackinnon AJ. 2016. Effectiveness of an online insomnia program (SHUTi) for prevention of depressive episodes (the GoodNight Study): a randomised controlled trial. Lancet 3(4): p333-341.
17. Corrigan JD, Bogner J. 2007. Initial reliability and validity of the Ohio State University TBI Identification Method. J Head Trauma Rehabil 22(6): 318-29.
18. Cozza SJ, Benedek DM, Bradley JC, Grieger TA, Nam TS, Waldrep DA. 2004. Topics specific to the psychiatric treatment of military personnel. In: Schnurr PP, Cozza SJ, editors. The Iraq War Clinician Guide. edn 2nd. National Center for PTSD and Department of Defense.
19. Faraone SV, et al. 2000. The drug-placebo response curve: a new method for assessing drug effects in clinical trials. J Clin Pscyhopharmacology 20(6): 673-679.
20. Field TA, Beeson ET, Jones LK. 2005. The New ABCs: A Practitioner's Guide to Neuroscience-Informed Cognitive-Behavior Therapy. Journal of Mental Health Counseling, 37 (3): 206–220.

21. Davidson JR, Dawson S, Krsmanovic A. 2017. Effectiveness of Group Cognitive Behavioral Therapy for Insomnia (CBT-I) in a Primary Care Setting. Behav Sleep Med 2:1-13.
22. Davy Z, Middlemass J, Siriwardena AN. 2013. Patients' and clinicians' experiences and perceptions of the primary care management of insomnia: qualitative study. Health Expectations 18(5):1371–1383.
23. Defense and Veterans Brain Injury Center. 2017. *DoD Worldwide Numbers for TBI*. Retrieved from http://dvbic.dcoe.mil/dod-worldwide-numbers-tbi
24. Demyttenaere K, De Fruyt J. 2003. Getting what you ask for: on the selectivity of depression rating scales. Psychother Psychosom 72(2): 61-70.
25. Devine JK, Scott-Sutherland J, Mullington J, Haack M. 2017. USEFULNESS OF WRIST ACTIGRAPHY SLEEP MEASURES IN PREDICTING FATIGUE IN INSOMNIA DISORDER. Sleep 40(Suppl 1): A154.
26. DeViva JC, Zayfert C, Mellman TA. 2004. Factors Associated With Insomnia Among Civilians Seeking Treatment for PTSD: An Exploratory Study. Behav Sleep Med 2(3): 162-176.
27. Epstein DR, Sidan S, Bootzin RR, Belyea MJ. 2012. Dismantling multicomponent behavioral treatment for insomnia in older adults: a randomized controlled trial. Sleep 35(6): 797-805.
28. Gagnon C, Belanger L, Ivers H, Morin CM. Validation of the Insomnia Severity Index in primary care. 2013. Validation of the Insomnia Severity Index in primary care. J Am Board Fam Med 26(6): 701-10.
29. Geiger-Brown JM, Rogers VE, Liu W, Ludeman EM, Downton KD, Diaz-Abad M. Cognitive behavioral therapy in persons with comorbid insomnia: A meta-analysis. 2015. Sleep Med Rev 23: 54-67.
30. Germain A, Hall M, Krakow B, Katherine Shear M, Buysse DJ. 2005. A brief sleep scale for Posttraumatic Stress Disorder: Pittsburgh Sleep Quality Index Addendum for PTSD. J Anxiety Disord 19(2):233-44.
31. Grandner MA, Kripke DF, Yoon I, Youngsted SD. 2006. Criterion validity of the Pittsburgh Sleep Quality Index: Investigation in a non-clinical sample. Sleep Biol Rhythms 4(2): 129-139.
32. Hauri PJ, Wisbey J. 1992. Wrist actigraphy in insomnia. Sleep 15: 293-301.
33. Hou L, Han X, Sheng P, Tong W, Li Z, Xu D, et al.. 2013. Risk Factors Associated with Sleep Disturbance following Traumatic Brain Injury: Clinical Findings and Questionnaire Based Study. PLoS ONE 8(10): e76087.
34. Jacobs GD, Pace-Schott EF, Stickgold R, et al. 2004. Cognitive Behavior Therapy and Pharmacotherapy for Insomnia. Arch Intern Med 164(17):1888-1896.
35. Jackowska M, Ronaldson A, Brown J, Steptoe A. 2016. Biological and psychological correlates of self-reported and objective sleep measures. J Psychosom Res 84: 52-55.
36. Jenkins MM, Colvonen PJ, Norman SB, Afari N, Allard CB, Drummond SPA. 2005. Prevalence and Mental Health Correlates of Insomnia in First-Encounter Veterans with and without Military Sexual Trauma. Sleep 38(10): 1547-1554.
37. Ji K, Kang M. 2017. STOP-Bang Questionnaire in Patients with Rapid Eye Movement Sleep Behavior Disorder. Sleep Med Res 8(2): 102-106.
38. Knutson KL, Ryden AM, Mander BA, Van Cauter E. 2006. Role of sleep duration and quality in the risk and severity of type 2 diabetes mellitus. Arch Intern Med 166:1768–1774.
39. Koffel E, Khawaja IS, Germain A. 2016. Sleep Disturbances in Posttraumatic Stress Disorder: Updated Review and Implications for Treatment. Psychiatr Ann 46(3): 173-176.
40. Koffel E, Kuhn E, Petsoulis N, et al. 2016. A randomized controlled pilot study of CBT-I Coach: Feasibility, acceptability, and potential impact of a mobile phone application for patients in cognitive behavioral therapy for insomnia. Health Inf J 24(1): 3-13.
41. Kravitz HM, Zheng H, Bromberger JT, Buysse DJ, Owens J, Hall MH. 2015. An Actigraphy Study of Sleep and Pain in Midlife Women – The SWAN Sleep Study. 22(7): 710-718.
42. Kroenke K, Spitzer RL, Williams JB. 2001. The PHQ-9: validity of a brief depression severity measure. J Gen Intern Med 16(9): 606-613.

43. Kuhn E, Weiss BJ, Taylor KL, Hoffmann JE, et al. 2016. CBT-I Coach: A Description and Clinician Perceptions of a Mobile App for Cognitive Behavioral Therapy for Insomnia. J Clin Sleep Med 12(4): 597-606.
44. Li, SX, Lam SP, Yu MWM, Zhang J, Wing YK. 2010. Nocturnal sleep disturbances as a predictor of suicide attempts among psychiatric outpatients: A clinical, epidemiologic, prospective study. J Clin Psychaitry 71(11): 1440-1446.
45. Margolies SO, Rybarczyk B, Vrana SR, Leszczyszn DJ, Lynch J. 2013. Efficacy of a Cognitive Behavioral Treatment for Insomnia and Nightmares in Afghanistan and Iraq Veterans With PTSD. J Clin Psyc 69(10): 1026-1042.
46. Marino M, Li Y, Rueschman MN et al. 2013. Measuring Sleep: Accuracy, Sensitivity, and Specificity of Wrist Actigraphy Compared to Polysomnography. Sleep 36(11): 1747-1755.
47. Mathews EE, Arnedt JT, McCarthy MS, Cuddihy LJ, Aloia MS. 2013. Adherence to Cognitive Behavioral Therapy for Insomnia: A Systematic Review. Sleep Med Rev 17(6): 453-464.
48. Mitchell MD, Gehrman P, Perlis M, Umscheid CA. 2012. Comparative effectiveness of cognitive behavioral therapy for insomnia: a systematic review. MC Fam Pract 13: 40.
49. Morin CM, Belleville G, Bélanger L, Ivers H. 2011. The insomnia severity index: psychometric indicators to detect insomnia cases and evaluate treatment response. SLEEP 34(5): 601-608.
50. Morin CM, Bootzin RR, Buysse DJ, Edinger JD, Espie CA, Lichstein KL. 2006. Psychological and behavioral treatment of insomnia: update of the recent evidence (1998–2004) Sleep 29:1398–1414.
51. Morin CM, Culbert JP, Schwartz SM. 1994. Nonpharmacological interventions for insomnia: a meta-analysis of treatment efficacy. Am J Psychiatry 151(8): 1172-1180.
52. Morgenthaler T, Alessi C, Friedman L, Owens J, Kapur V, Boehlecke B et al. 2007. Practice parameters for the use of actigraphy in the assessment of sleep and sleep disorders: an update for 2007. Sleep 30: 519–529.
53. Mysliwiec V, Gill J. Lee H. Baxter T, Pierce R, Barr TL, Krakow B, Roth BJ. 2013. Sleep Disorders in US Military Personnel. Chest 144(2): 549-557.
54. Nappi CM, Drummond SPA, Hall JMH. 2012. Treating Nightmares and Insomnia in Posttraumatic Stress Disorder: A Review of Current Evidence. Neuropharmacology 62(2):: 576-585.
55. Naqappa M, Liao P, Wong J, et al. 2015. Validation of the STOP-Bang Questionnaire as a Screening Tool for Obstructive Sleep Apnea among Different Populations: A Systematic Review and Meta-Analysis. PLoS One 10(12): e0143697.
56. O'Brien P and Flemming T. 1979. A multiple testing procedure for clinical trials. Biometrics 35:549-556.
57. Okajima I, Komada Y, Inoue Y. 2010. A meta analysis on the treatment effectiveness of cognitive behavioral therapy for primary insomnia. Sleep and Biological Rhythms 9(1): 24-34.
58. Parthasarathy S, Vasquez MM, Halonen M, et al. 2015. Persistent Insomnia is Associated with Mortality Risk. Am J Med 128(3): 268-275.e2.
59. Percileous P. 2016. Parametric joint modeling for longitudinal and survival data (Doctoral thesis). School of Computing Sciences. University of East Anglia, UK. Accessed at https://ueaeprints.uea.ac.uk/id/eprint/59673
60. Peters S et al. 2012. Multiple imputation of missing repeated outcome measurements did not add to linear mixed-effects models. J Clin Epidem 65(6): 686-695.
61. Phillips B, Mannino DM. 2007. Do insomnia complaints cause hypertension or cardiovascular disease? J Clin Sleep Med 3:489–494.
62. Ponsford JL, Ziino C, Parcell DL, Shekleton JA, Roper M, Redman JR, Phipps-Nelson J, Rajaratnam S. 2012. Fatigue and Sleep Disturbance Following Traumatic Brain Injury - Their Nature, Causes, and Potential Treatments. J Head Trauma 27(3): 224-233.
63. President's Commission on Care for America's Returning Wounded Warriors. 2007. *Serve, support, simplify: Report of the President's commission on care for America's returning wounded*

warriors, in President's Commission on Care of America's Returning Wounded Warriors. Retrieved from Veterans for America Website.

64. Revel FG, Gottowik J, Gatti S, Wettstein JG, Moreau JL. 2009. Rodent models of insomnia: a review of experimental procedures that induce sleep disturbances. Neurosci Biobehav Rev 33:874–899.
65. Ribeiro JD, Pease JL, Gtuerrez PM, Silva C, Berenert RA, Rudd MD, Joiner TE. 2012. Sleep problems outperform depression and hopelessness as cross-sectional and longitudinal predictors of suicidal ideation and behavior in young adults in the military. J Affective Dis 136(3): 743-750.
66. Riedal B, Lichstein KL, Peterson BA, Means MK, Epperson MT, Aguillarel RN. 1998. A comparison of the efficacy of stimulus control for medicated and nonmedicated insomniacs. Behavior Modification 22: 3-28.
67. Riemann D, Voderholzer U. 2003. Primary insomnia: a risk factorto develop depression? J Affective Dis 76(1-3): 255-259.
68. Richardson GS. 2007. Human physiological models of insomnia. Sleep Med 8 Suppl. 4:S9–S14
69. Ritterband LM, Thorndike FP, Gonder-Frederick LA, Magee JC, Bailey ET, Saylor DK, Morin CM. 2009. Efficacy of an Internet-based behavioral intervention for adults with insomnia. Arch Gen Psychiatry 66(7): 692-8.
70. Ritterband LM et al. 2017. Effect of a web-based cognitive behavior therapy for insomnia intervention with 1-year follow-up. JAMA Pschiatry 74(1): 68-75.
71. Robins JM et al. 2000. Marginal structural models and causal inference in epidemiology. Epidemiology 11(5): 550-560.
72. Roth T. 2007. Insomnia: Definition, Prevalence, Etiology, and Consequences. J Clin Sleep Med 3(5 Suppl): S7-S10.
73. Sack RL, Auckley D, Auger RR, Carskadon MA, Wright KP, Vitiello MV, et al. 2007. Circadian rhythm sleep disorders: part II, advanced sleep phase disorder, delayed sleep phase disorder, free-running disorder, and irregular sleep- wake rhythm. Sleep 30: 1484-501.
74. Sadeh A. 2011. The role and validity of actigraphy in sleep medicine: An update. Sleep Med Rev 15(4): 259-267.
75. Siebern AT, Manber R. 2011. New developments in cognitive behavioral therapy as the first-line treatment of insomnia. Psychol Res Behav Manag 4: 21-28.
76. Singareddy R, Vgontzas AN, Fernandez-Menodoza J, Liao D, Calhoun, Shaffer ML, Bixler EO. 2012. Risk factors for incident chronic insomnia: A general population prospective study. Sleep Med 13(4): 346-353.
77. Spielman AJ, Saskin P, Thorpy MJ. 1987. Treatment of chronic insomnia by restriction of time in bed. Sleep 10(1): 45-56.
78. Stepanski EJ, Wyatt JK. 2003. Use of sleep hygiene in the treatment of insomnia. Sleep Med Rev 7(3): 215-225.
79. Stewart WF, Lipton RB, Dowson AJ, Sawyer J. 2001. Development and testing of the Migraine Disability Assessment (MIDAS) Questionnaire to assess headache-related disability. Neurology 56(6 Suppl 1): S20-8.
80. Swanson TM, Isaacson BM, Cyborski CM, French LM, Tsao JW, Pasquina PF. 2017. Traumatic Brain Injury Incidence, Clinical Overview, and Policies in the US Military Health System Since 2000. Public Health Reports 132(2):251-259.
81. Taylor DJ, Lichstein KL, Weinstock, Sanford S, Temple JR. 2007. A Pilot Study of Cognitive-Behavioral Therapy of Insomnia in People with Mild Depression. Beh Ther 38(1): 49-57.
82. Taylor DJ, Peterson AL, Pruiksma KE, Young-McCaughan S, Nicholson K, Mintz J, and STRONG STAR Consortium. 2017. Internet and In-Person Cognitive Behavioral Therapy for Insomnia in Military Personnel: A Randomized Clinical Trial. Sleep 40(6): online edition.
83. Taylor DJ, Pruiksma KE. 2014. Cognitive and behavioural therapy for insomnia (CBT-I) in psychiatric populations: A systematic review. Int Rev Psychiatry 26(2): 205-213.

84. Taylor DJ, Pruiksma KE, Hale WJ, et al. 2016. Prevalence, Correlates, and Predictors of Insomnia in the US Army prior to Deployment. Sleep 39(10): 1795-1806.
85. Thorndike FP, Ritterband LM, Gonder-Frederick LA, Lord HR, Ingersoll KS, Morin CM. 2013. A randomized controlled trial of an internet intervention for adults with insomnia: effects on comorbid psychological and fatigue symptoms. J Clin Psychol 69(10): 1078-93.
86. Turner RM, Ascher LM. 1979. Controlled comparison of progressive relaxation, stimulus control, and paradoxical intention therapies for insomnia. Journal of Consulting and Clinical Psychology 47: 500-508.
87. Troxel WM, Conrad TS, Germain A, Buysse DJ. 2013. Predictors of treatment response to brief behavioral treatment of insomnia (BBTI) in older adults. J Clin Sleep Med 9(12): 1281-9.
88. Ulmer CS, Bosworth HB, Beckham JC, Germain A, Jeffreys AS, Edelman D, Macy S, Kirby A, Voils CI. 2017. Veterans Affairs Primary Care Provider Perceptions of Insomnia Treatment. J Clin Sleep Med 13(8):991-999.
89. US Department of Health and Human Services. 2018. *Veterans Crisis Line*. Retrieved from the Veterans Crisis Line website.
90. Viola-Saltzman M and Watson NF. Traumatic Brain Injury and Sleep Disorders. Neurol Cin. 2012 Nov; 30(4): 1299-1312.
91. Wu JQ, Appleman ER, Salazar RD, Ong JC. 2015. Cognitive Behavioral Therapy for Insomnia Comorbid With Psychiatric and Medical Conditions: A Meta-analysis. JAMA Intern Med 175(9): 1461-1472.

GLOSSARY

AE – Adverse Event
ANOVA – Analysis of Variance
AUC – Area Under the Curve
BDI-II – Beck Depression Inventory II
CBT – Cognitive Behavioral Therapy
CBT-I – Cognitive Behavioral Therapy for Insomnia
CNRM – Center for Neuroscience and Regenerative Medicine
CRF – Case Report Form
DHA – Defense Health Agency
DHHS – Department of Health and Human Services
DoD – Department of Defense
EDC – Electronic Data Capture
FACIT-F – Functional Assessment of Chronic Illness Therapy for Fatigue
FITBIR – Federal Interagency Traumatic Brain Injury Research
GUID – Global Unique Identifier
HIPAA – Health Insurance Portability and Accountability Act
HJF – Henry M. Jackson Foundation
IRB – Institutional Review Board
ISI – Insomnia Severity Index
ITT – Intention to Treat
MTF – Military Treatment Facility
mTBI – Mild Traumatic Brain Injury
MANOVA – Multivariate Analysis of Variance
MIDAS – Migraine Disability Assessment Test
NIH – National Institutes of Health
OSA – Obstructive Sleep Apnea
PHI – Protected Health Information
PCL-5 – Posttraumatic Stress Disorder Checklist for DSM-5
PCL-M – Posttraumatic Stress Disorder Checklist Military Version
PHQ-9 – Patient Health Questionnaire-9 for Depression
PI – Principal Investigator
PII – Personal Identifying Information
PSQI - Pittsburgh Sleep Quality Index
PSQI-A – Pittsburgh Sleep Quality Index Addendum for PTSD
RBD – Rapid-Eye Movement Sleep Behavior Disorder
SAE – Serious Adverse Event
TBI – Traumatic Brain Injury
UPIRTSO – Unanticipated Problems Involving Risks to Subjects or Others
USU – Uniformed Services University
VA – Department of Veteran Affairs

SHUTI

SPONSOR: MTBI^2 / USUHS

STATISTICAL ANALYSIS PLAN

VERSION DATE: September 26, 2022

This document is proprietary to MTBI^2
It is intended for those specifically authorized to receive it.
Any other use or disclosure is prohibited.

ACKNOWLEDGEMENT

SPONSOR: MTBI^2/ USUHS

PROTOCOL NUMBER: CNRM-92-9662

The signatures below indicate that these individuals have reviewed this project-specific statistical analysis plan and acknowledgement this document as governing these particular tasks.

Thaddeus Haight, PhD	MTBI^2 Statistician
**Print Name**	**Title**
	9-26-2022
**Signature**	**Date**

David Brody	Principal Investigator (PI)
**Print Name**	**Title**
	26SEPT2022
**Signature**	**Date**

This analysis plan should be reviewed by all study staff and signed by the PI, acknowledging understanding of study operations and requirements.

VERSION HISTORY

This Statistical Analysis Plan (SAP) for protocol number CNRM-92-9662 is based on protocol version 1.11.

Version	Date	Description of Change	Brief Rationale
SAP Version 1	09-26-2022	Initial version of statistical plan developed based on protocol version 1.11.	Statistical plan development based on protocol.

TABLE OF CONTENTS

1. INTRODUCTION 7
 1.1. Objectives and Endpoints 7
  1.1.1. Objectives 7
  1.1.2. Endpoints 8
 1.2. Study Design 9
 1.3. Randomization and Blinding 9
2. STATISTICAL HYPOTHESES 9
3. SAMPLE SIZE DETERMINATION 10
 3.1. Background 10
 3.2. Sample size calculation for mean difference in ISI score between treatment and control groups 10
 3.3. Sample size calculation for AUC 11
4. ANALYSIS SETS 12
5. GENERAL CONSIDERATIONS 12
 5.1. Participant Dispositions 12
 5.2. Missing Data 13
6. PRIMARY ENDPOINT ANALYSIS 14
 6.1. Definition of Endpoints 14
 6.2. Analytical Approach 14
7. SECONDARY ENDPOINT ANALYSIS 16
 7.1. Definition of Endpoints 16
 7.2. Analytical Approach 17
8. EXPLORATORY ANALYSIS 18
 8.1. Definition of Endpoints 18
 8.2. Analytical Approach 18
9. SAFETY ANALYSIS 19
10. INTERIM ANALYSIS 19
11. ADDITIONAL ANALYSES 19

11.1. Sensitivity analyses of missing data 19
12. REFERENCES 20

ABBREVIATIONS

AE	Adverse Event
AUC	Area under the curve
CASA	CNRM Collection, Access, Sharing and Analytics system
CNRM	Center for Neuroscience and Regenerative Medicine
DSMB	Data Safety Monitoring Board
eCBT-I	electronic cognitive behavioral therapy for insomnia
FACIT-F	Functional Assessment of Chronic Illness Therapy – Fatigue
IA	Interim Analysis
IRB	Institutional Review Board
ISI	Insomnia Severity Index
LMM	Linear Mixed Effects Model
MAR	Missing at random
MIDAS	Migraine Disability Assessment
MNAR	Missing not at random
mTBI	mild traumatic brain injury
MTF	military treatment facility
PCL-5	PTSD Checklist for DSM-5
PHQ-9	Patient Health Questionnaire-9
PSQI	Pittsburgh Sleep Quality Index
SAE	serious adverse event
SAP	Statistical Analysis Plan
TBI	traumatic brain injury

1. INTRODUCTION

The following document describes the Statistical Analysis Plan (SAP) for the study entitled, A Randomized, Controlled Study of Internet-guided Cognitive Behavioral Therapy for Insomnia in Military Traumatic Brain Injury. It is to be used in conjunction with the study design and statistical plan described in the study protocol. The document makes references to the study protocol.

1.1. Objectives and Endpoints

1.1.1. Objectives

The objective of the study is to determine the feasibility and efficacy of eCBT-I compared to an active control condition for primary insomnia in US military service members with history of TBI.

Primary Objective

To evaluate changes in insomnia severity index (ISI) score in an eCBT-I treatment group versus a sleep education control group in active and retired service members who have insomnia with a history of TBI.

Secondary Objectives

- To assess changes in depression symptom severity as measured by the Patient Health Questionnaire 9 for Depression (PHQ-9) (Kroenke et al., 2001) with sleep question #3 redacted for redundancy.
- To assess changes in PTSD-related symptoms as measured by the PTSD Checklist for DSM-5 (PCL-5) (Belvins et al., 2015).
- To assess changes in migraine-related symptoms as measured by the Migraine Disability Assessment (MIDAS) (Stewart et al., 2001).
- To assess changes in sleep quality as measured by the Pittsburgh Sleep Quality Index (PSQI) (Germain et al., 2005).
- To assess changes in fatigue-related symptoms as measured by the Functional Assessment of Chronic Illness Therapy-Fatigue (FACIT-F) (Butt et al., 2013)

Exploratory Objectives

- To assess participant blinding efficacy as measured by believed treatment group assignment and actual treatment group assignment.
- To assess participant expectation of benefit of intervention as measured by pre-treatment and post-treatment questionnaires and changes in ISI score from baseline to post-intervention.

- To assess participant likelihood of recommending intervention to friends and family and changes in ISI score from baseline to post-intervention.
- To assess participant rating of efficacy of intervention and changes in ISI score from baseline to post-intervention.
- To assess participant rating of usability of intervention and changes in ISI score from baseline to post-intervention.

1.1.2. Endpoints

Primary Endpoint

- **Insomnia Severity Index (ISI)**. Change in ISI score, where ISI score is measured by a personally filled questionnaire, between baseline (Day 2-7 of study) and post-treatment evaluation (Day 71 of study).

Secondary Endpoints

- **Insomnia Severity Index (ISI)**. Change in ISI score, where ISI score is measured by a personally filled questionnaire, between baseline (Day 2-7 of study) and 3-month follow-up evaluation (Day 160 of study).

- **Pittsburgh Sleep Quality Index (PSQI).** Change in PSQI score, where PSQI score is measured by a personally filled questionnaire, between baseline (Day 2-7 of study) and post-treatment (Day 71) and 3-month follow-up evaluations (Day 160 of study), respectively.

- **PTSD Checklist for DSM-5 (PCL-5).** Change in PCL-5 score, where PCL-5 score is measured by a personally filled questionnaire, between baseline (Day 2-7 of study) and post-treatment (Day 71) and 3-month follow-up evaluations (Day 160 of study), respectively.
- **Patient Health Questionnaire (PHQ-9).** Change in PHQ-9 score, where PHQ-9 score is measured by a personally filled questionnaire, between baseline (Day 2-7 of study) and post-treatment (Day 71) and 3-month follow-up evaluations (Day 160 of study), respectively.
- **Migraine Disability Assessment (MIDAS).** Change in MIDAS score, where MIDAS score is measured by a personally filled questionnaire, between baseline (Day 2-7 of study) and post-treatment (Day 71) and 3-month follow-up evaluations (Day 160 of study), respectively.
- **Functional Assessment of Chronic Illness Therapy – Fatigue (FACIT-F).** Change in FACIT-F score, where FACIT-F score is measured by a personally filled questionnaire, between baseline (Day 2-7 of study) and post-treatment (Day 71) and 3-month follow-up evaluations (Day 160 of study), respectively.

Exploratory Endpoints

- **Participant blinding efficacy.** Participant believed treatment group assignment (3-levels: uncertain, control group, eCBT-I group)
- **Participant rating of expected treatment benefit.** (3 levels: Low, Moderate, High)
- **Participant rating of perceived treatment efficacy.** (3 levels: Low, Moderate, High)
- **Participant rating of perceived treatment usability.** (3 levels: Low, Moderate, High)
- **Participant rating of likelihood in recommending treatment to family and friends.** (3 levels: Unlikely, Moderately likely, Highly likely)

1.2. Study Design

This is an internet-based, controlled, prospective, randomized interventional trial with an optional open-label treatment. Up to 200 subjects will be randomized to either eCBT-I or sleep education control groups in a 3:1 ratio, respectively. The study will be entirely internet-based with no in-person contact between the study team and the participants. At the end of the study, participants randomized to control will be offered open-label access to the eCBT-I treatment. The study includes a patient assessment at baseline, a post-treatment assessment at approximately 9 weeks following consent, and a 3-month follow-up evaluation. Immediately following the 3-month follow-up evaluation, the study includes an open-label/safety extension phase of approximately 9 weeks.

1.3. Randomization and Blinding

This study minimizes potential bias through use of randomized assignment of treatment group. Participants will not be formally blinded, but will not be informed which arm, eCBT-I or sleep education (i.e., control), is expected to be more effective. After signing the informed consent, participants will be randomized 3:1 to either receive eCBT-I or control, respectively. Randomization will take place through a centralized database utilizing the CASA system. Participant information will be entered into the system, and a randomization code will be provided to the study team. This number will correspond with eCBT-I or control.

2. STATISTICAL HYPOTHESES

The primary hypothesis of interest of the study and statistical analysis is that the decrease in ISI score between the baseline assessment and post-treatment assessment will be *greater* in the eCBT-I group *relative to* the control group.

3. SAMPLE SIZE DETERMINATION

The study sample size was pre-specified for N=200. Power analyses were applied to assess the sample size needed to detect a significant change in ISI score between eCBT-I vs. control groups that assumed this study N, as well as for varying sample size N < 200.

3.1.Background

Previous reports that have examined ISI with respect to eCBT-I intervention have found significant effects (Ritterband et al., 2009; Ritterband et al., 2017). Given measures of mean difference, and variability of the ISI measure, in these reports, data were simulated and power was evaluated based on different conditions including: (1) sample size (pre-specified for N=200, but investigated for lower N); (2) mean difference in ISI between treatment and control groups; (3) assumed arm imbalance 3:1 vs. 1:1; (4) variability of the outcome measures under evaluation; and (5) study attrition.

3.2. Sample size calculation for mean difference in ISI score between treatment and control groups

To assess if the sample size for the current study was sufficient, we applied power analyses for detection of a significant treatment effect for the primary analysis---i.e., analysis of treatment difference (i.e., treatment vs. control group) in ISI score (9 week – 1 week), using a linear mixed effects model for the effects of interest – i.e., Y~Treatment + Time + Treatment x Time. Power was evaluated with respect to the Treatment x Time effect from this model – i.e., the parameter that would indicate an effect of treatment on change in ISI score (week 9 - week 1), based on a two-sided test and α=0.05. In this analysis, different conditions were modified to evaluate their respective effects on power. Differences in group means in ISI score were simulated to be close to zero at week 1 and approximately 5 at week 9 (note: Ritterband et al. (2017) reported mean differences of ~ 0 (SD = 5.5) and 5 (SD=5.5) at pre/post-assessment).

Table 1. Power estimates of treatment effects pre- and post-treatment (1 week-9 weeks) under different conditions.

Arm Balance	Mean Difference	Std dev	Total N	Power
3:1	5	5.5	200	1
3:1	5	5.5	160	1
3:1	5	5.5	120	1
3:1	5	8.5	200	0.99
3:1	5	8.5	160	0.96
3:1	5	8.5	120	0.90
3:1	3	5.5	200	0.95
3:1	3	5.5	160	0.89

3:1	3	5.5	120	0.79
3:1	3	8.5	200	0.64
3:1	3	8.5	160	0.54
3:1	3	8.5	120	0.43
1:1	5	8.5	200	0.99
1:1	5	8.5	160	0.99
1:1	5	8.5	120	0.96

Although the pre-specified study N is 200 (eCBT-I N=150 and control arm N=50, respectively), the analysis above indicates that the study would still be sufficiently powered given study attrition. Power would vary under reduced study N given mean difference in ISI score between the eCBT-I and control groups, and the variability of the ISI score. For example, in Table 1, for N=120 with a 3:1 arm allocation, given a mean difference in change in ISI score between treatment and control groups = 3 and ISI score variability in both groups SD = 5.5, the power is approximately 80%.

3.3.Sample size calculation for AUC

Power curves were generated in the case of proposed analyses to examine AUC (see sec 6.2.2 below) assuming different AUC (treatment vs. control responses) for ISI score (note: AUC=0.5 represents no treatment effect). Differences in sample size were compared for the different curves to reflect potential dropout rates of 0%, 20% and 40% in the study (Fig 1).

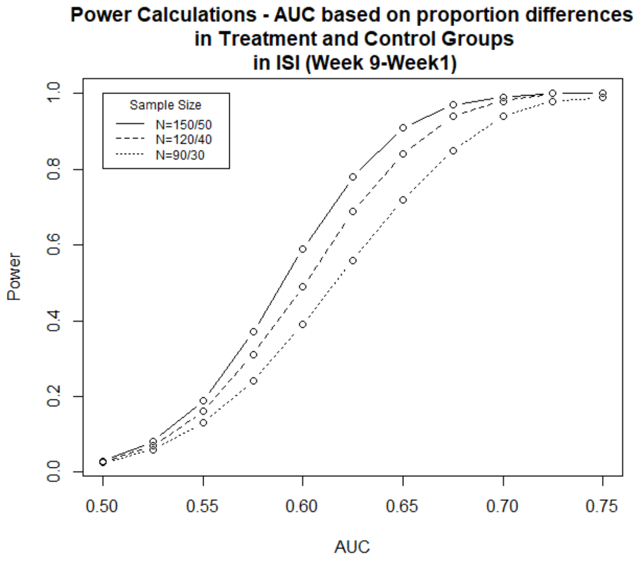

Fig 1. Calculated power of AUC analysis as affected by varying estimated sample size.

4. ANALYSIS SETS

The full analysis set (FAS) will be participants who are randomized to the study. The efficacy analysis set, which is a subset of the FAS, will be used to analyze the efficacy endpoints using intention-to-treat analysis. Therefore, the efficacy analysis set will include all participants with the primary endpoint measured at the baseline assessment. Participants may or may not be measured at the post-treatment or 3-month follow-up assessment but nevertheless be included in the efficacy analysis set. Analysis will be conducted under a missing at random assumption (MAR) (note: sensitivity analyses will be conducted in the event MAR cannot be assumed), and based according to the original treatment arm assignment to which each participant was randomized. The per-protocol analysis set will be a subset of the efficacy analysis set that includes participants who completed all eCBT-I or sleep education modules (‘as-treated’), completed all assessments (i.e., baseline, post-treatment, 3-month follow-up), and had no major protocol violations.

5. GENERAL CONSIDERATIONS

Analysis of the primary outcome of interest in the trial will be to compare changes in ISI score between Days 2-7 (week 1) and Day 71 (week 9) of the study between the eCBT-I and control groups. Analysis of secondary outcomes of interest will be to examine change for a variety of secondary outcome measures between Days 2-7 and Day 71 of the study in the eCBT-I group and to compare these with changes observed in ISI score. Exploratory analyses will examine various measures reported by the participants with respect to: 1) perceived treatment assignment; 2) perceived treatment benefit; 3) perceived treatment efficacy; 4) perceived treatment usability; and 5) patient recommendation of treatment to family and friends, given participants treatment group assignment.

Summary descriptive statistics by treatment group will be tabulated at each visit. For continuous endpoints, descriptive statistics will include number of participants, mean, median, standard deviation, 25th and 75th percentiles, minimum and maximum values. For categorical endpoints, descriptive statistics will include number of participants and percentages.

5.1. Participant Dispositions

The study population includes male or female military service members or civilians $\geq$ 18 and $\leq$ 64 years of age, with an indication of clinical insomnia for at least 1 month prior to consent (ISI

score >14 and PSQI >4), as well as a history of TBI >=6 months prior to consent as documented in medical records or confirmed by a TBI Screener.

We provide a schematic outline that describes patient dispositions for the study.

A. Screened for eligibility
    a. Included in study
    b. Excluded from study
B. Randomization
    a. Allocated to placebo
        i. Received allocated placebo
    b. Allocated to treatment
        i. Received allocated treatment
C. Follow-up
    a. Completed Study
    b. Discontinued Study
        i. Patient Request
        ii. Lost to follow-up
D. Analysis
    a. Analysis for Efficacy
    b. Excluded from analysis
    c. Safety Analysis

5.2. Missing Data

In order to advance through the different eCBT-I training and education modules (online study portal), participants need to complete weekly assessments related to the primary and some secondary outcome measures (i.e., ISI, fatigue). Therefore, data will not be missing intermittently during the trial period (i.e., 1 week - 9 week). However, participants may decide to drop out of the study before completion of the entire protocol which could result in right censored or missing data beyond the time point at which participant leaves the trial. Different analytical strategies will be applied. Descriptive statistics of treatment arm assignment, demographic data, and baseline assessments related to the primary and secondary outcomes will be compared in those with and without missing data at follow-up. If the data are missing at random (i.e., distributions of demographic data and baseline assessments for the missing and non-missing groups are similar), the current analysis plan utilizing linear mixed-effects models will be implemented, which essentially imputes the missing values in subjects who are missing with mean data of subjects who completed the protocol (Peters et al., 2012; Bell et al., 2013; Carpenter and Smuk, 2021). If data are not missing at random, an analysis of the data utilizing linear mixed-effects models will still be conducted for the primary and secondary endpoints as indicated below, in addition to a sensitivity analysis for missing data (See Section 11.1 for details).

6. PRIMARY ENDPOINT ANALYSIS

6.1. Definition of Endpoints

Change in ISI score between baseline (Day 2-7) and post-treatment evaluation (Day 71). The endpoint will be evaluated using a linear mixed effect model (LMM) (see below) that analyzes the *change* in ISI score between baseline and post-treatment. ISI score is measured at three different timepoints in the study (baseline, post-treatment, 3-month evaluation). The analysis assumes that ISI score is normally distributed at each of these timepoints, which will be evaluated using the Shapiro-Wilk test. If ISI score is not normally distributed, then suitable transforms will be tested to attempt to normalize the data. If a suitable transform cannot be found, then non-parametric Mann Whitney U-tests will be performed.

6.2. Analytical Approach

6.2.1. Linear Mixed Effects Model (LMM) Analysis. Analyses will be based on intention-to-treat (ITT) as it pertains to participant's original group assignment. First, an initial standard analysis will examine the ISI change between week 1 (pre-treatment) and week 6-9 (post-treatment) between the active and control groups. Assuming ISI measures are normally distributed, the following mixed model could be applied to assess changes in ISI score post-intervention compared to pre-intervention in active and control groups:

$$E[Y_{ijk}|\text{Treatment, Time}] = \alpha_i + \beta_0 + \beta_{1j}\,\text{Treatment} + \beta_{2k}\,\text{Time} + \beta_{3jk}\,\text{Treatment x Time} \quad \text{(Model 1)}$$

where Y_{ijk} represents ISI score in the *i*th person, *j*th group (1=active, 0=control) and *k*th timepoint (1=6-9 weeks, 0=1 week). Based on the model, β_1 represents the mean difference in ISI score between groups at week 1, β_2 represents the mean difference in ISI at 6-9 weeks vs. 1 week in the control group, and β_3 represents the mean difference in ISI score at 6-9 weeks vs. 1 week in the treatment *relative to* the control group. A two-sided test with alpha=0.05 would test the significance of the treatment x time effect represented by the β_3 coefficient. Additional parameters in the model include β_0 which represents the mean ISI score in the control group at 1 week and α_i which represents an individual's random effect to account for within-subject correlation.

For efficiency, Model 1 can be extended to include change in ISI between week 1 and the 3-month evaluation, but can be used to assess the primary endpoint of interest as well. For example, the LMM

$$E[Y_{ij}|\text{Treatment}, \text{Time}_1, \text{Time}_2] = \alpha_i + \beta_0 + \beta_{1j}\,\text{Treatment} + \beta_2\,\text{Time}_1 + \beta_3\,\text{Time}_2 + \beta_{4j}\,\text{Treatment} \times \text{Time}_1 + \beta_{5j}\,\text{Treatment} \times \text{Time}_2 \qquad \text{(Model 2)}$$

includes time indicator variables to represent post-treatment (Time_1) and 3-month (Time_2) assessment periods. Based on the model, β_1 represents the mean difference in ISI score between groups at week 1, β_2 represents the mean difference in ISI score at 6-9 weeks vs. 1 week in the control group, β_3 represents the mean difference in ISI score at 3-months vs. 1 week in the control group, β_{4j} represents the mean difference in ISI score at 6-9 weeks vs. 1 week in the treatment *relative to* the control group, and β_{5j} represents the mean difference in ISI score at 3-months vs. 1 week in the treatment *relative to* the control group. For the primary endpoint analysis, a two-sided test with alpha=0.05 would test the significance of the treatment x time effect represented by the β_{4j} coefficient.

6.2.2. Area under the Curve (AUC) Analysis. Given subject-to-subject variability with respect to completion of the eCBT-I protocol within the treatment window period ranging between 6 to 9 weeks (i.e., time between baseline assessment and post-treatment evaluation may vary between participants), an additional analysis of group difference in ISI score will be examined using area under the curve (AUC). Based on AUC methods proposed by Faraone et al. (2000), the AUC method applied to the current study would examine ISI differences (week 6-9 – week 1) - i.e., negative differences would indicate symptom improvement - between the active and control groups. Specifically, differences in ISI score (D) for the entire study group would be ranked from lowest (i.e. negative) to highest (positive differences). For each D, the cumulative percentage of respondents from the active and control groups would be determined, plotted with respect to y and x axes representing proportion of respondents in these two groups, and a treatment-response curve would be drawn. The AUC, measured with respect to this treatment – response curve, will be examined with respect to AUC=0.5 (i.e., null difference), as typically reported in ROC analyses, using a two-sided test and α=0.05.

7. SECONDARY ENDPOINT ANALYSIS

7.1. Definition of Endpoints

7.1.1. Change in Insomnia Severity Index (ISI) score between baseline (Day 2-7) and 3-month follow-up evaluation (Day 160).

7.1.2. Change in Pittsburgh Sleep Quality Index (PSQI) between baseline (Day 2-7) and post-treatment (Day 71) and 3-month follow-up evaluations (Day 160), respectively.

7.1.3. Change in PTSD Checklist for DSM-5 (PCL-5) between baseline (Day 2-7) and post-treatment (Day 71) and 3-month follow-up evaluations (Day 160), respectively.

7.1.4. Change in Patient Health Questionnaire (PHQ-9) between baseline (Day 2-7) and post-treatment (Day 71) and 3-month follow-up evaluations (Day 160), respectively.

7.1.5. Change in Migraine Disability Assessment (MIDAS) between baseline (Day 2-7) and post-treatment (Day 71) and 3-month follow-up evaluations (Day 160), respectively.

7.1.6. Change in Functional Assessment of Chronic Illness Therapy – Fatigue (FACIT-F) between baseline (Day 2-7) and post-treatment (Day 71) and 3-month follow-up evaluations (Day 160), respectively.

For endpoint 7.1.1, the endpoint will be evaluated using a LMM that analyzes the change in ISI score between baseline and 3-month evaluation. For efficiency, the same model used for the primary endpoint analysis (sec 6.2.1, Model 2) can be used for the secondary endpoint analysis involving ISI. For endpoints 7.1.2 – 7.1.6, the endpoints will be evaluated using LMMs that analyze the change in the respective endpoints between baseline and the 6-9 week and 3-month evaluation periods, respectively. These analyses assume each of the endpoints used in these analyses is normally distributed at each of the different timepoints, which will be evaluated using the Shapiro-Wilk test. If these endpoints are not normally distributed, then suitable transforms will be tested to attempt to normalize the data. If a suitable transform cannot be found, then non-parametric Mann Whitney U-tests will be performed.

7.2. Analytical Approach

7.2.1. Analysis to examine change in ISI score between baseline and 3-month evaluation period.

Analyses will be based on intention-to-treat (ITT) as it pertains to participant's original group assignment. For efficiency, the same model used for the primary endpoint analysis (sec 6.2.1, Model 2) can be used for the secondary endpoint analysis involving ISI:

$$E[Y_{ij}|\text{Treatment, Time}_1\text{, Time}_2] = \alpha_i + \beta_0 + \beta_{1j}\text{ Treatment} + \beta_2\text{ Time}_1 + \beta_3\text{ Time}_2 + \beta_{4j}\text{ Treatment x Time}_1 + \beta_{5j}\text{ Treatment x Time}_2 \quad \text{(Model 2)}$$

where β_{5j} represents the mean difference in ISI score at 3-months vs. 1 week in the treatment *relative to* the control group (See sec 6.2.1 for a description of different parameters included in the model). To evaluate the secondary outcome change measure for ISI (endpoint 7.1.1), a two-sided test with alpha=0.05 would test the significance of the treatment x time effect represented by the β_{5j} coefficient.

7.2.2. Analyses to examine change in secondary endpoint measures between baseline, post-treatment and 3-month evaluation periods.

Change in measures considered to be associated with ISI that could potentially be affected by the eCBT-I intervention will be examined using different models in participants randomized to that intervention group. Separate analyses will examine change in these secondary endpoint measures (7.1.2-7.1.6) between baseline and post-treatment and 3-month evaluation periods, respectively. For example, in the LMM:

$$E[Y_i|\text{Time}_1\text{, Time}_2] = \alpha_i + \beta_0 + \beta_1\text{ Time}_1 + \beta_2\text{ Time}_2 \quad \text{(Model 3)}$$

time indicator variables are used to represent post-treatment (Time_1) and 3-month (Time_2) assessment periods. Based on the model, β_1 represents the mean change in a given endpoint at 6-9 weeks vs. 1 week and β_2 represents the mean change in the same endpoint at 3-months vs. 1 week. Other model parameters include β_0, which represents the mean of a given endpoint at 1 week (i.e., baseline), and α_i which represents an individual's random effect to account for within-subject correlation. Two-sided tests with alpha=0.05 will be used to test the parameter estimates β_1 and β_2, to assess the significance of the mean change for each given endpoint at post-treatment

and the 3-month evaluation, respectively, compared to baseline, in participants assigned to eCBT-I.

7.2.3. Spearman correlations of change in ISI and change in secondary endpoints.

Separate Spearman correlations will be used to examine the change in ISI and change in secondary endpoints (7.1.2-7.1.6) for the time period between a) baseline and post-treatment and b) baseline and the 3-month evaluation, in the eCBT-I and control groups. Two-sided tests of significance with alpha=0.05 will be used to assess the significance of each correlation.

8. EXPLORATORY ANALYSES

8.1. Definition of Endpoints

8.1.1. Participant blinding efficacy. (3-levels: uncertain, control group, eCBT-I group)

8.1.2. Participant rating of expected treatment benefit (3 levels: Low, Moderate, High)

8.1.3. Participant rating of perceived treatment efficacy. (3 levels: Low, Moderate, High)

8.1.4. Participant rating of perceived treatment usability. (3 levels: Low, Moderate, High)

8.1.5. Participant rating of likelihood in recommending treatment to family and friends. (3 levels: Unlikely, Moderately likely, Highly likely)

8.2. Analytical Approach

The study includes different measures of participant perception of the treatment. Each of these measures will be compared with change in ISI from baseline to post-treatment in the eCBT-I and control groups. For the expected benefit of treatment measure, analyses will examine the relationship of change in ISI between baseline and post-treatment and expected benefit, prior to starting treatment, across the assigned groups (i.e., eCBT-I vs control). The same relationship will be examined post-intervention also. Other measures (efficacy, usability, likelihood of recommendation to friend/family member) vs change in ISI, between baseline and post-treatment, will be examined at post-intervention in the two groups. Lastly, participant blinding efficacy will be examined by comparing participants' believed group assignment vs. actual group assignment. The analysis will examine these patient categories against patient change in ISI between baseline and post-treatment.

9. SAFETY ANALYSIS

Data will be collected based on participants' experience of AEs and SAEs. For example, AEs will be reported for the treatment and control arms as percentage occurrence. If there are a sufficient number of events, the distribution of AEs will be assessed using chi-square or similar statistical test of association.

10. INTERIM ANALYSES

Interim analyses will be conducted with n=100 and n=150 participants, respectively, who completed the protocol. To maintain a type 1 error = 0.05 and power = 0.9, based on an analysis of the entire sample (n=200), an alpha-spending approach was applied such that larger critical values were calculated for the first and second interim analyses required for rejecting the null hypothesis (i.e., no mean difference in ISI score between active and control groups) (DeMets and Lan, 1994). These larger critical values correspond with significance tests (p-values) of 0.004 and 0.0196 at the first and second analysis, respectively. The significance test required for the third (and final) analysis, based on the full sample, is slightly lower ($p < 0.045$) as result of including the interim analyses. Stopping boundaries were based on methods developed for sequential design and provide critical values at different stages that would approximate $\alpha=0.05$ given an analysis of the full sample (O'Brien and Fleming, 1979). Analyses that indicate significant difference in ISI score plus depression and PTSD scores jointly, between pre- and post-treatment assessments, based on these reduced p-values, will result in early termination of the trial due to treatment efficacy. To assess group differences with respect to the joint distribution of these measures, we will employ multivariate analysis of variance (MANOVA). Changes in scores for the respective outcome measures will be examined as dependent variables with respect to group assignment. An overall test, as well as individual tests of difference, of the dependent measures will be assessed and a decision will be made to terminate the study.

11. ADDITIONAL ANALYSES

11.1. Sensitivity analyses of missing data

Sensitivity analyses of missing data will occur pending the proportion of participants with missing data at follow-up is > 10% and evidence of informative censoring – i.e., that missing data at follow-up are related to underlying study characteristics (e.g., demographic, baseline assessments). Methods are available that model and account for missingness based on observed study data (e.g. predictors of patients with missing data versus patients without missing data). Patients who complete the study, who share similar covariate distributions as those with missing information or are lost-to-follow-up, are up weighted, based on these predictors, to account for patients without data [Robins, 2002].

Missing data may occur as the result of unmeasured variables – i.e., variables that are not collected as part of the study and, therefore, cannot be modeled using available study data. If required, sensitivity analyses will utilize tipping analysis methods [Yuan, 2014; Mehrotra, 2017]. In tipping analysis, a shift parameter is employed as part of an imputation analysis with the missing data – e.g., an offset of the treatment effect in participants with missing data relative to those with complete data. The shift parameter is changed incrementally until inference related to the intervention changes (e.g., non-significant treatment effect) from the inference based on the complete (i.e., non-missing) data). If the shift parameter represents a plausible value that results in a non-significant treatment effect, the study results may need to be reconsidered.

Inferences based on the efficacy analyses and sensitivity analyses that account for those with missing data as outlined above will be compared to assess the extent to which missing data may affect the study findings.

12. REFERENCES

Bell M. L., Kenward M.G., Fairclough D.L., & Horton N. J. (2013). Differential dropout and bias in randomized controlled trials: when it matters and when it may not. *BMJ*, 346, e8668.

Blevins C. A., Weathers F. W., Davis M. T., Witte T. K., & Domino J. L. (2015). The Posttraumatic Stress Disorder Checklist for DSM-5 (PCL-5): Development and initial psychometric evaluation. *Journal of Traumatic Stress*, 28, 489-498.

Butt Z., Lai J. S., Rao D., Heinemann A. W., Bill A., & Cella D. (2013). Measurement of fatigue in cancer, stroke, and HIV using the Functional Assessment of Chronic Illness Therapy - Fatigue (FACIT-F) scale. *J Psychosom Res*, 74(1): 64-8.

Carpenter J.R. & Smuk M. (2021). Missing data: A statistical framework for practice. *Biometrical Journal*,1-33.

Demets D., & Lan K. (1994). Interim analysis: The alpha spending function approach. *Statistics in Medicine*, 13, 1341-1352.

Faraone S.V., Biederman J., Spencer T.J., & Wilens T.E. (2000). The drug-placebo response curve: a new method for assessing drug effects in clinical trials. *J of Clin Pscyhopharmacology*, 20(6): 673-679.

Germain A., Hall M., Krakow B., Katherine Shear M., & Buysse D.J. (2005). A brief sleep scale for Posttraumatic Stress Disorder: Pittsburgh Sleep Quality Index Addendum for PTSD. *J Anxiety Disord*, 19(2):233-44.

Kroenke K., Spitzer R.L., & Williams J.B. (2001). The PHQ-9: validity of a brief depression severity measure. *J Gen Intern Med*, 16(9): 606-613.

O'Brien P.C. & Fleming T.R. (1979). A multiple testing procedure for clinical trials. *Biometrics*, 35:549–556.

Mehrotra D., Liu F., & Permutt T. (2017). Missing data in clinical trials: control-based imputation and sensitivity analysis. *Pharmaceutical Statistics*, 16:378-392.

Percileous P. (2016). Parametric joint modeling for longitudinal and survival data (Doctoral thesis). School of Computing Sciences. University of East Anglia, UK. Accessed at https://ueaeprints.uea.ac.uk/id/eprint/59673

Peters, S.A., Bots, M.L., den Ruijter, H.M,, Palmer M.K., Grobbee, D.E., Crouse J.R., … & Koffijberg H. The METEOR Study Group. (2012). Multiple imputation of missing repeated outcome measures did not add to linear mixed-effects models. *Journal of Clinical Epidemiology*, 65(6), 686-695.

Ritterband L.M., Thorndike F.P., Gonder-Frederick L.A., Magee J.C., Bailey E.T., Saylor D.K., & Morin C.M. (2009). Efficacy of an internet-based behavioral intervention for adults with insomnia. *Arch Gen Psychiatry*, 2009; 66(7): 692-698.

Ritterband L.M., Thorndike F.P., Ingersoll K.S., Lord H.R., Gonder-Frederick L.A., Frederick C., Quigg M.S., Cohn W.F., & Morin C.M. (2017). Effect of a web-based cognitive behavior therapy for insomnia intervention with 1-year follow-up. *JAMA Pschiatry*,74(1): 68-75.

Robins, J.M., Hernan M.A., & Brumback B. (2002). Marginal structural models and causal inference in epidemiology. *Epidemiology*, *11*(5), 550-560.

Stewart W.F., Lipton R.B., Dowson A.J., & Sawyer J. (2001). Development and testing of the Migraine Disability Assessment (MIDAS) Questionnaire to assess headache-related disability. *Neurology*, 56(6 Suppl 1): S20-8.

Yuan Y. (2014). Sensitivity analysis in multiple imputation for missing data. SAS Institute Inc. Paper SAS270-2014.
