# Supplementary material for: Internet-Guided Cognitive Behavioral Therapy for Insomnia Among Patients With Traumatic Brain Injury: A Randomized Clinical Trial
Source: JAMA Netw Open. 2024 Jul 9;7(7):e2420090. doi: 10.1001/jamanetworkopen.2024.20090 (PMC11234237; doi:10.1001/jamanetworkopen.2024.20090)
Supplement: Supplement 2. — eFigure 1. Expected Benefit Prior to Starting the Intervention vs Change in ISI From Baseline to Post Intervention eFigure 2. Believed Group Assignment vs Change in ISI From Baseline to Post Intervention eFigure 3. Participant Ratings of the Intervention vs Change in ISI From Baseline to Post Intervention eFigure 4. Interaction Between Sleep Medication Use and Group Assignment in Effects on ISI eFigure 5. As-Treated Analyses of Primary and Key Secondary Outcome Measures Involving Only Participants Who Completed All Online Modules and All Assessments eFigure 6. Correlations Between Changes in Self-Reported Insomnia and Changes in PTSD Symptom Severity, With Spearman ρ Values eFigure 7. Correlations Between Changes in Self-Reported Insomnia and Changes in Self-Reported Sleep Quality, With Spearman ρ Values eFigure 8. Correlations Between Changes in Self-Reported Insomnia and Changes in Self-Reported Fatigue Impact, With Spearman ρ Values eFigure 9. Correlations Between Changes in Self-Reported Insomnia and Changes in Migraine-Related Disability, With Spearman ρ Values eTable 1. Demographic Characteristics of Participants Who Completed Immediate Post Intervention Follow-Up Evaluations eTable 2. Demographic Characteristics of Participants Who Completed 3-Month Follow-Up Evaluations eTable 3. Baseline Clinical Scores for Participants Who Completed vs Were Missing Immediate Postintervention Follow-Up Evaluations eTable 4. Baseline Clinical Scores for Participants Who Completed vs Were Missing 3-Month Post-Intervention Follow-Up Evaluation eTable 5. Demographics for Participants Who Completed vs Were Missing Immediate Post-Intervention Follow-Up Evaluations eTable 6. Demographics for Participants Who Completed vs Were Missing at 3-Month Post Intervention Follow-Up Evaluations eMethods. Statistical Analysis eAppendix. Additional Limitations eReferences [file jamanetwopen-e2420090-s002.pdf]

Supplementary Online Content

Malarkey ME, Fu AJ, Mannan N, et al. Internet-guided cognitive behavioral therapy for insomnia among patients with traumatic brain injury: a randomized clinical trial. *JAMA Netw Open*. 2024;7(7):e2420090. doi:10.1001/jamanetworkopen.2024.20090

eFigure 1. Expected Benefit Prior to Starting the Intervention vs Change in ISI From Baseline to Post Intervention
eFigure 2. Believed Group Assignment vs Change in ISI From Baseline to Post Intervention
eFigure 3. Participant Ratings of the Intervention vs Change in ISI From Baseline to Post Intervention
eFigure 4. Interaction Between Sleep Medication Use and Group Assignment in Effects on ISI
eFigure 5. As-Treated Analyses of Primary and Key Secondary Outcome Measures Involving Only Participants Who Completed All Online Modules and All Assessments
eFigure 6. Correlations Between Changes in Self-Reported Insomnia and Changes in PTSD Symptom Severity, With Spearman ρ Values
eFigure 7. Correlations Between Changes in Self-Reported Insomnia and Changes in Self-Reported Sleep Quality, With Spearman ρ Values
eFigure 8. Correlations Between Changes in Self-Reported Insomnia and Changes in Self-Reported Fatigue Impact, With Spearman ρ Values
eFigure 9. Correlations Between Changes in Self-Reported Insomnia and Changes in Migraine-Related Disability, With Spearman ρ Values
eTable 1. Demographic Characteristics of Participants Who Completed Immediate Post Intervention Follow-Up Evaluations
eTable 2. Demographic Characteristics of Participants Who Completed 3-Month Follow-Up Evaluations
eTable 3. Baseline Clinical Scores for Participants Who Completed vs Were Missing Immediate Postintervention Follow-Up Evaluations
eTable 4. Baseline Clinical Scores for Participants Who Completed vs Were Missing 3-Month Post-Intervention Follow-Up Evaluation
eTable 5. Demographics for Participants Who Completed vs Were Missing Immediate Post-Intervention Follow-Up Evaluations
eTable 6. Demographics for Participants Who Completed vs Were Missing at 3-Month Post Intervention Follow-Up Evaluations
eMethods. Statistical Analysis
eAppendix. Additional Limitations
eReferences

This supplementary material has been provided by the authors to give readers additional information about their work.

© 2024 Malarkey ME et al. *JAMA Network Open*.

eFigure 1. Expected Benefit Prior to Starting the Intervention vs Change in ISI From Baseline to

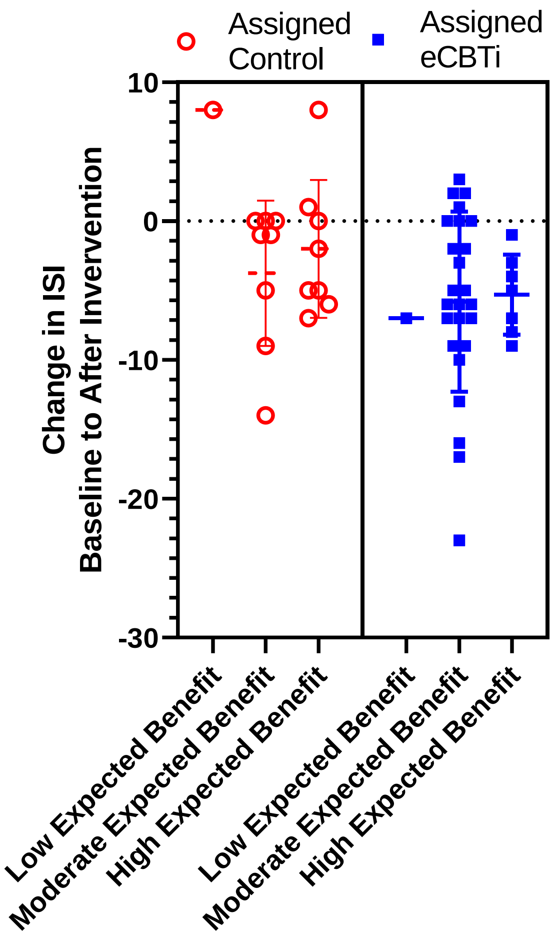

Post Intervention

No significant differences between participants with moderate vs. high expected benefit.

© 2024 Malarkey ME et al. *JAMA Network Open*.

eFigure 2. Believed Group Assignment vs Change in ISI From Baseline to Post Intervention

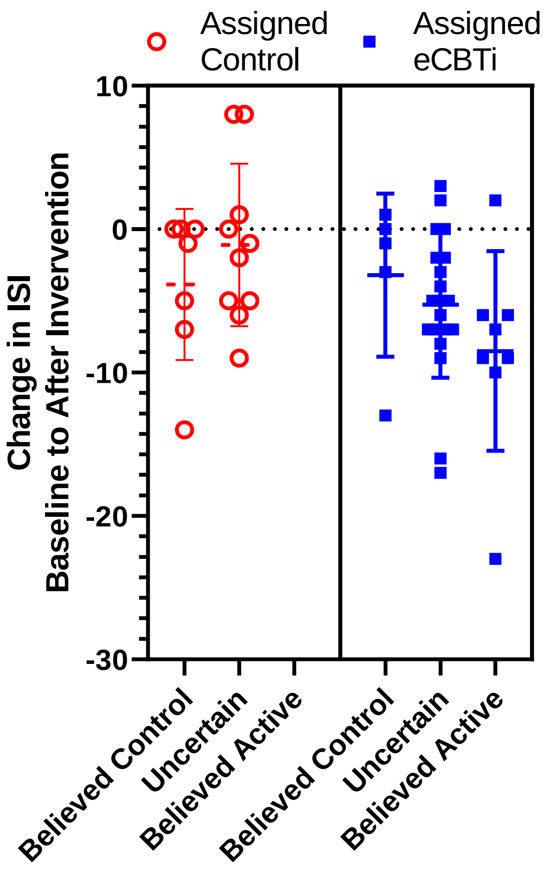

Participants who believed they had received the active intervention also reported greater decreases in insomnia than those who were uncertain or believed they had received the control intervention. No participants who were assigned to control believed that they had been assigned to the active intervention, though 5 participants assigned to active intervention believed that they had been assigned to control.

© 2024 Malarkey ME et al. *JAMA Network Open*.

eFigure 3. Participant Ratings of the Intervention vs Change in ISI From Baseline to Post Intervention

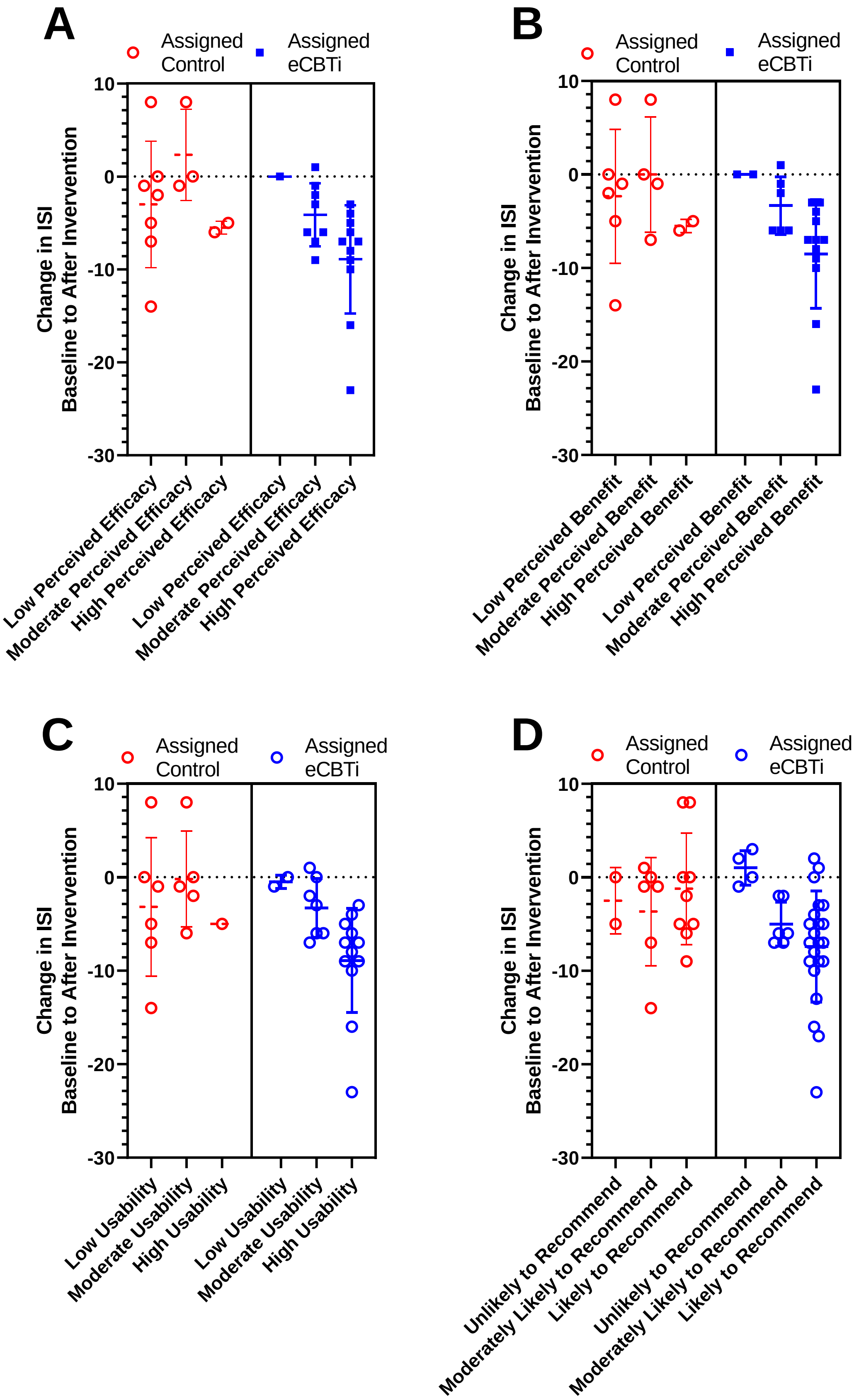

© 2024 Malarkey ME et al. *JAMA Network Open*.

A. Efficacy, **B.** Perceived benefit, **C.** Usability, **D.** Likelihood of recommending the intervention to friends and family.

© 2024 Malarkey ME et al. *JAMA Network Open*.

eFigure 4. Interaction Between Sleep Medication Use and Group Assignment in Effects on ISI

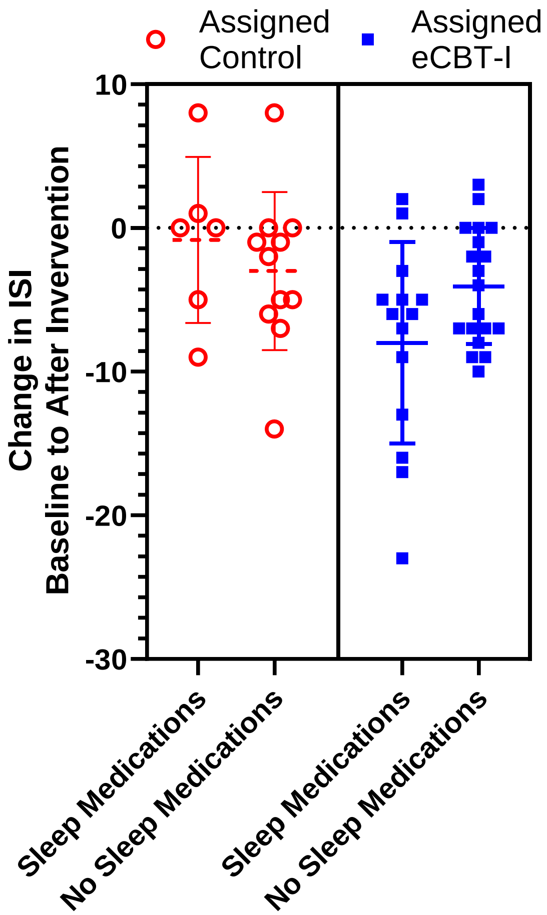

There were reductions in ISI in those assigned to eCBT-I with and without sleep medication use. A two-way ANOVA using sleep medication use (yes vs. no) and group assignment (eCBT-I vs. control) demonstrated a significant main effect of group assignment favoring eCBT-I ($F_{1,46}$=5.8, p=0.0199), no significant main effect of sleep medication use ($F_{1,46}$=0.27, p=0.60), and no significant interaction ($F_{1,46}$=3.2, p=0.079). Medications reported for sleep included clonazepam, eszopiclone, gabapentin, medical marijuana, melatonin, prazosin topiramate, trazodone, and zolpidem.

© 2024 Malarkey ME et al. *JAMA Network Open*.

eFigure 5. As-Treated Analyses of Primary and Key Secondary Outcome Measures Involving Only Participants Who Completed All Online Modules and All Assessments

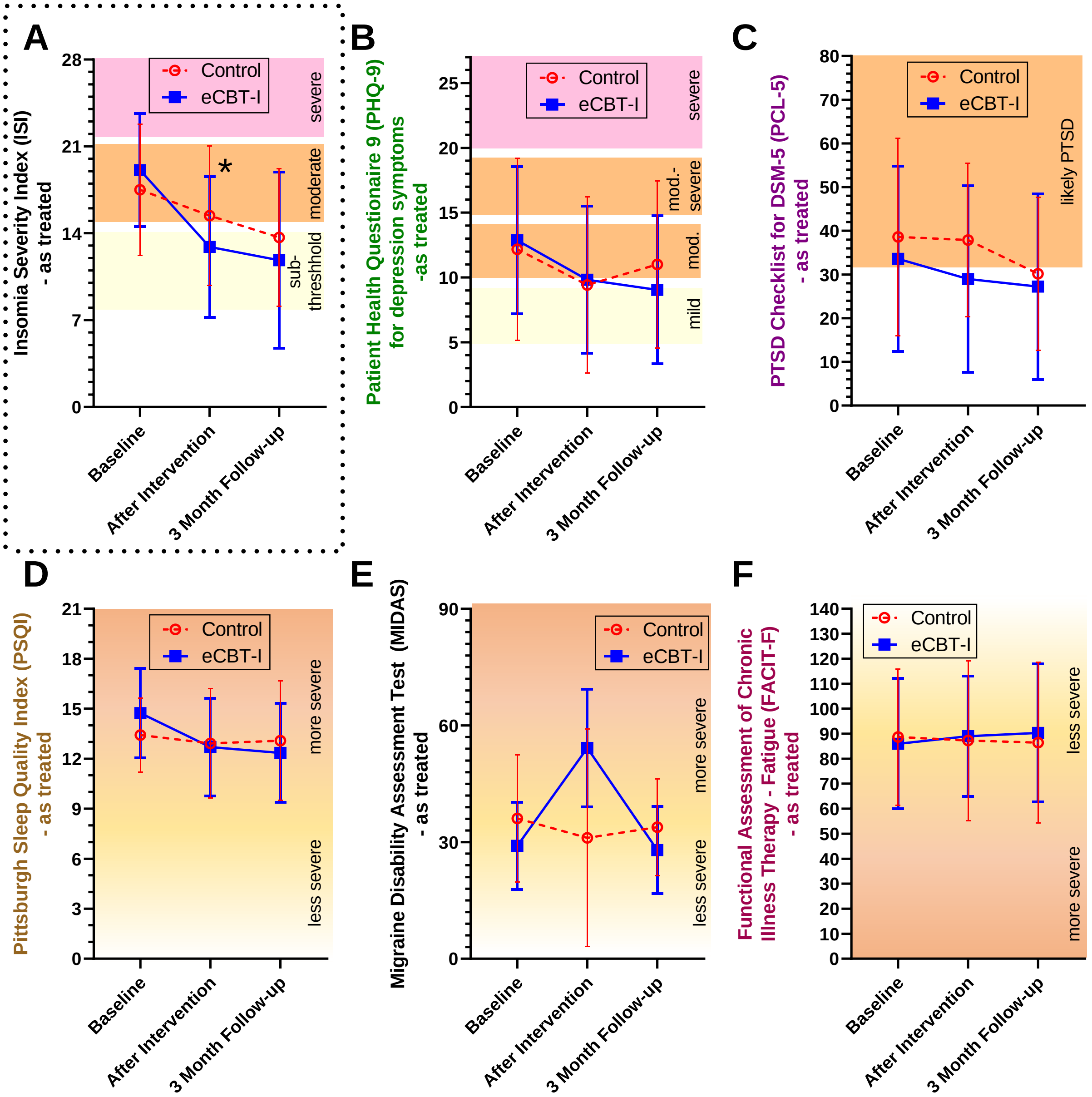

A. Self-reported insomnia severity, the primary outcome measure, **B.** Depression symptom severity, **C.** PTSD symptom severity, **D.** Self-reported sleep quality, **E.** Migraine-related disability, **F.** Fatigue impact. Data reported as a function of group (eCBT-I vs. education control) and assessment time point. Sample sizes were n=23 and n=12 for the eCBT-I and control groups respectively. Error bars indicate standard deviations. * indicates $p<0.05$ difference between groups.

© 2024 Malarkey ME et al. *JAMA Network Open*.

eFigure 6. Correlations Between Changes in Self-Reported Insomnia and Changes in PTSD Symptom Severity, With Spearman ρ Values

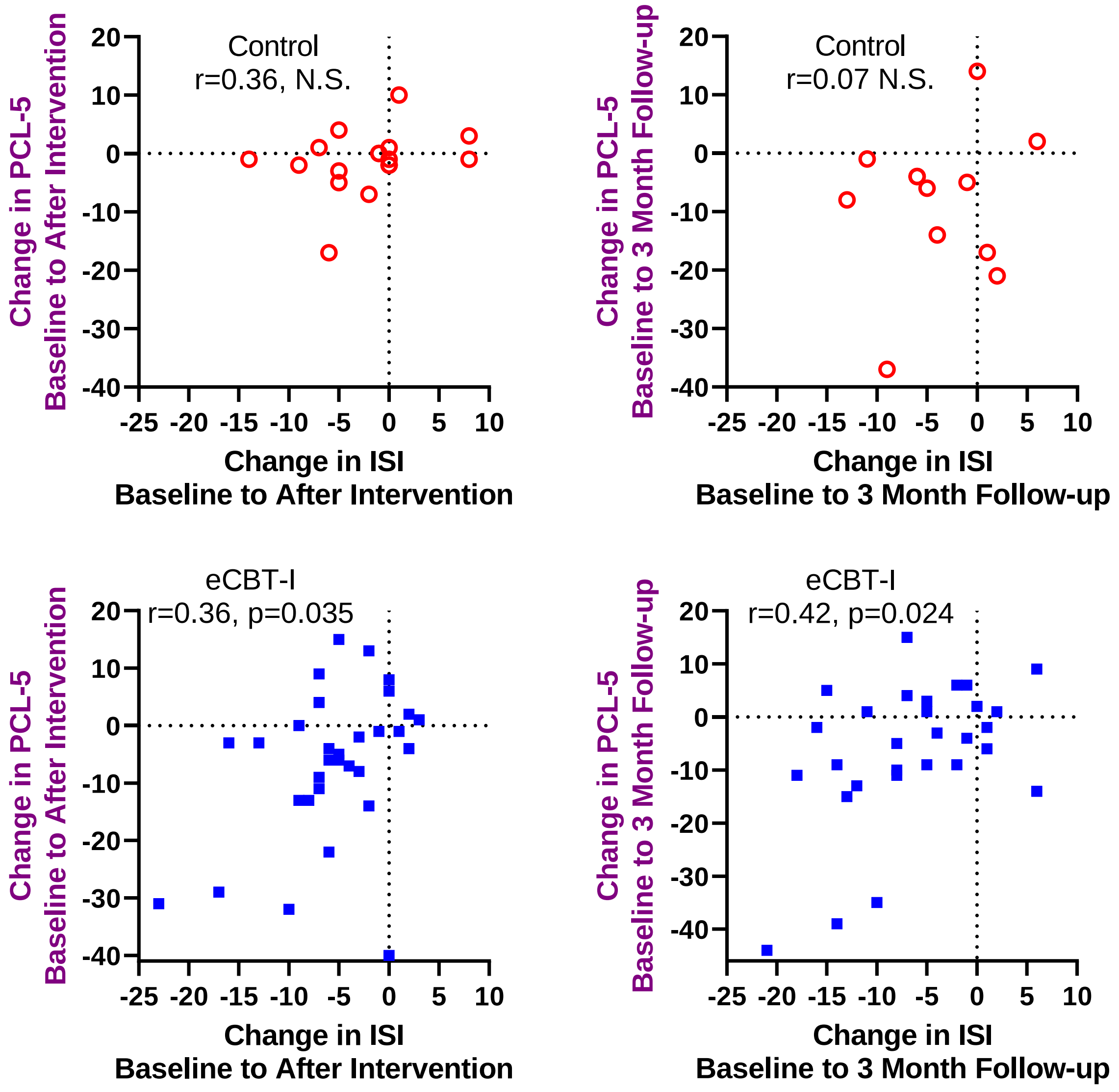

© 2024 Malarkey ME et al. *JAMA Network Open*.

eFigure 7. Correlations Between Changes in Self-Reported Insomnia and Changes in Self-Reported Sleep Quality, With Spearman ρ Values

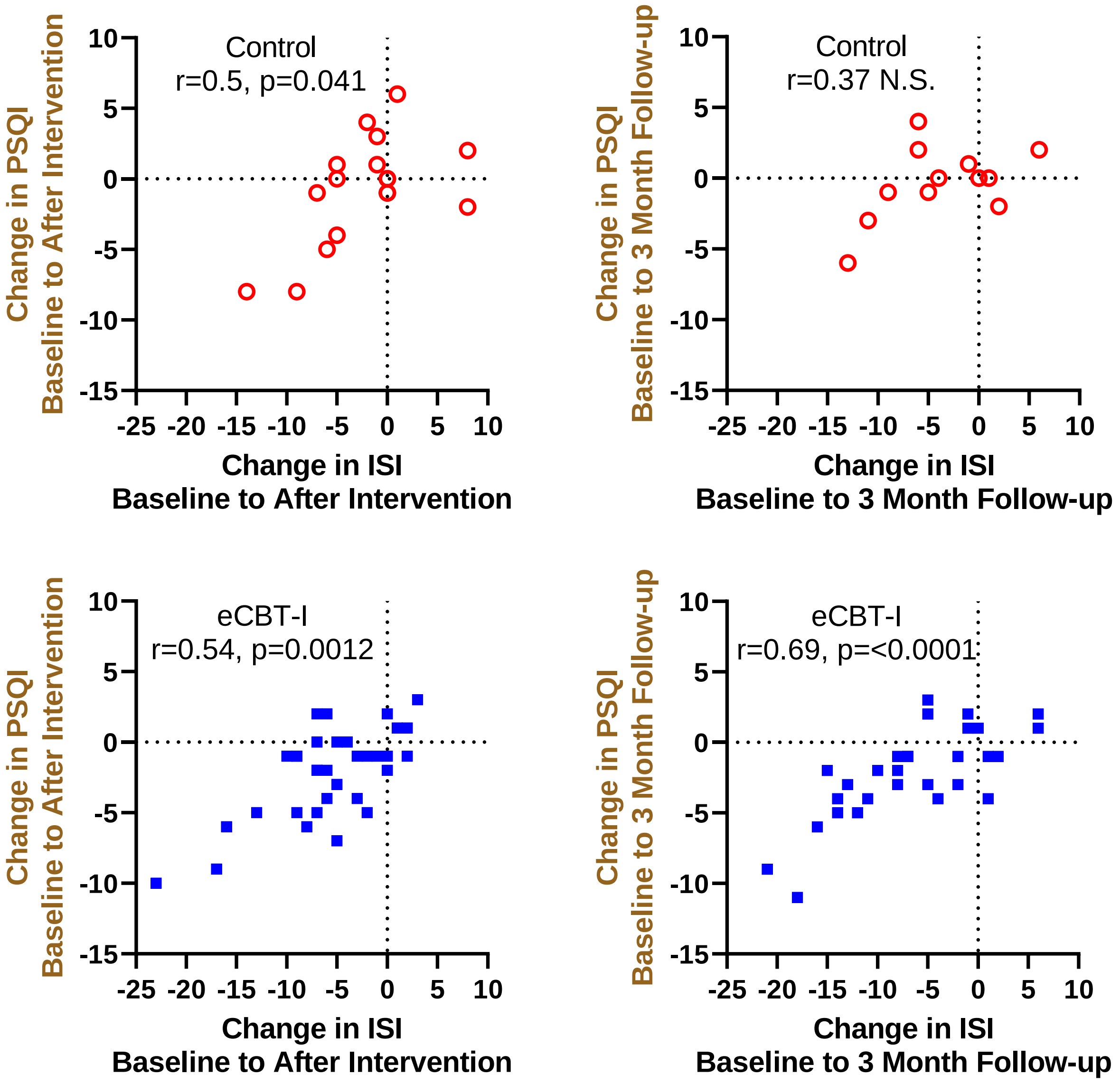

© 2024 Malarkey ME et al. *JAMA Network Open*.

eFigure 8. Correlations Between Changes in Self-Reported Insomnia and Changes in Self-Reported Fatigue Impact, With Spearman ρ Values

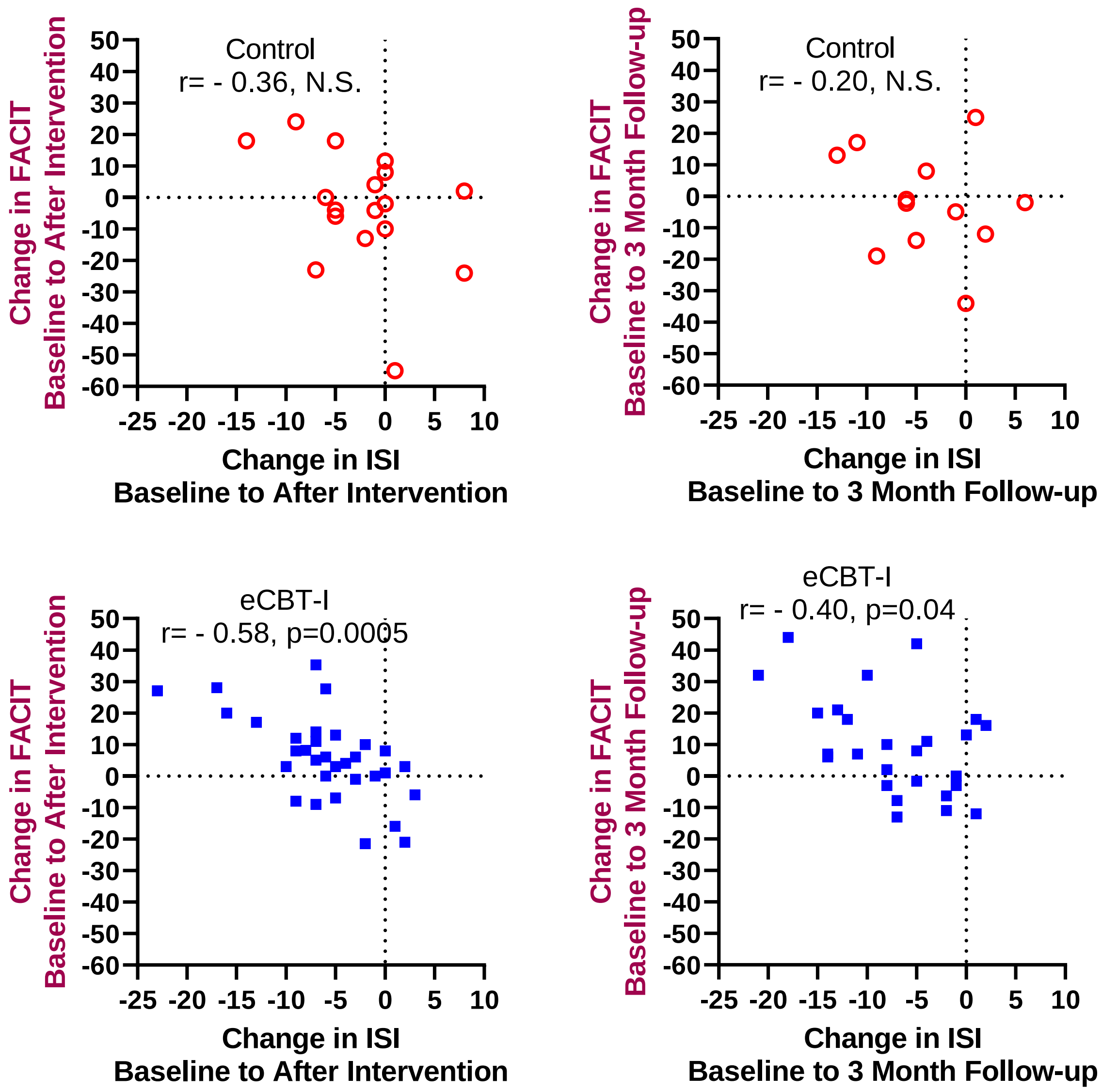

Increased FACIT scores represent improvement in fatigue impact.

© 2024 Malarkey ME et al. *JAMA Network Open*.

eFigure 9. Correlations Between Changes in Self-Reported Insomnia and Changes in Migraine-Related Disability, With Spearman ρ Values

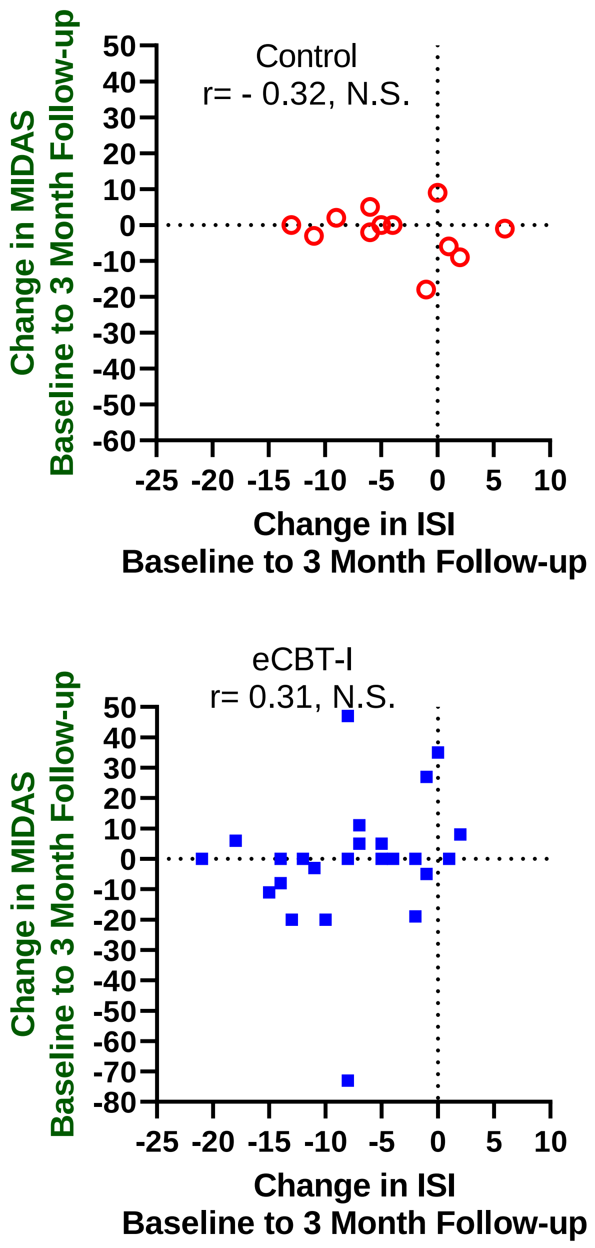

The MIDAS is based on headaches over the previous 90 days, so only changes from baseline to the 3-month follow-up time point are presented. Furthermore, since the MIDAS questions 2 and 4 are often misinterpreted by participants, a modified MIDAS score consisting of the sum of responses to questions 1, 3, and 5 was analyzed.

© 2024 Malarkey ME et al. *JAMA Network Open*.

eTable 1. Demographic Characteristics of Participants Who Completed Immediate Post Intervention Follow-Up Evaluations

Characteristic, No. (%)	Control (n = 17)	eCBT-I (n = 33)
Age, years, mean (SD)	44.06 (11.93)	46.45 (8.55)
Female	2 (11.76%)	9 (27.27%)
Race		
White	10 (58.82%)	26 (78.79%)
Black or African American	2 (11.76%)	2 (6.06%)
Asian	1 (5.88%)	0 (0%)
Native Hawaiian or other Pacific	0 (0%)	1 (3.03%)
Islander	2 (11.76%)	2 (6.06%)
Multiple races	2 (11.76%)	2 (6.06%)
Ethnicity		
Hispanic or Latino	4 (23.53%)	7 (21.21%)
Non-Hispanic or Latino	13 (76.47%)	26 (78.79%)
Educational level		
High school degree or less	1 (5.88%)	2 (6.06%)
Some college or college degree	5 (11.76%)	16 (48.48%)
Graduate Degree	11 (64.71%)	15 (45.45%)
US Geographic Region		
West	2 (11.76%)	2 (6.06%)
Midwest	0 (0%)	1 (3.03%)
South	12 (70.59%)	22 (66.67%)
Northeast	0 (0%)	0 (0%)
No response	3 (17.65%)	8 (24.24%)
Active Duty v. Retired		
Active Duty	13 (76.47%)	24 (72.73%)
Retired	4 (23.53%)	9 (27.27%)
Employment Status		
Full-time	12 (70.59%)	24 (72.73%)
Part-time	0 (0%)	2 (6.06%)
Unemployed	5 (29.41%)	7 (21.21%)
Military Branch		
Air Force	1 (5.88%)	2 (6.06%)
Army	11 (64.71%)	21 (63.64%)
Marine Corps	1 (5.88%)	5 (15.15%)
Navy	3 (17.65%)	5 (15.15%)
Other	1 (5.88%)	0 (0%)
Rank		
Junior Enlisted	5 (29.41%)	3 (9.09%)
Non-Commissioned Officer	5 (29.41%)	10 (30.30%)
Officer	6 (35.29%)	17 (51.52%)
Warrant Officer	0 (0%)	1 (3.03%)
Other	1 (5.88%)	1 (3.03%)
No response		1 (3.03%)
Military Occupation Type		
Combat	12 (70.59%)	22 (66.67%)
Non-Combat	5 (29.41%)	11 (33.33%)

Baseline ISI, mean (SD)	18.3 (5.0)	19.5 (4.1)

No statistically significant differences between groups.

© 2024 Malarkey ME et al. *JAMA Network Open*.

eTable 2. Demographic Characteristics of Participants Who Completed 3-Month Follow-Up Evaluations

Characteristic, No. (%)	Control cohort (n = 12)	Treatment cohort (n = 29)
Age, years, mean (SD)	46.58 (11.54)	46.90 (8.67)
Female	1 (8.33%)	9 (31.03%)
Race		
White	7 (58.33%)	21 (72.41%)
Black or African American	1 (8.33%)	3 (10.34%)
Asian	1 (8.33%)	1 (3.45%)
Multiple races	1 (8.33%)	2 (6.90%)
Other	1 (8.33%)	2 (6.90%)
Unknown	1 (8.33%)	0 (0%)
Ethnicity		
Hispanic or Latino	3 (25.00%)	5 (17.24%)
Non-Hispanic or Latino	9 (75.00%)	24 (82.76%)
Educational level		
High school degree or less	0 (0%)	3 (10.34%)
Some college or college degree	2 (16.67%)	11 (37.93%)
Graduate Degree	10 (83.33%)	15 (51.72%)
US Geographic Region		
West	1 (8.33%)	2 (6.90%)
Midwest	0 (0.00%)	0 (0%)
South	11 (91.67%)	19 (65.52%)
Northeast	0 (0.00%)	0 (0%)
No response	0 (0.00%)	8 (27.59%)
Active Duty v. Retired		
Active Duty	8 (66.67%)	20 (68.97%)
Retired	4 (33.33%)	9 (31.03%)
Employment Status		
Full-time	8 (66.67%)	21 (72.41%)
Part-time	0 (0%)	3 (10.34%)
Unemployed	4 (33.33%)	5 (17.24%)
Military Branch		
Air Force	1 (8.33%)	1 (3.45%)
Army	8 (66.67%)	19 (65.52%)
Marine Corps	0 (0%)	4 (13.79%)
Navy	2 (16.67%)	5 (17.24%)
Other	1 (8.33%)	0 (0%)
Rank		
Junior Enlisted	3 (25%)	3 (10.34%)
Non-Commissioned Officer	4 (33.33%)	6 (20.69%)
Officer	5 (41.67%)	16 (55.17%)
Warrant Officer	0 (0%)	2 (6.90%)
Other	0 (0%)	1 (3.45%)
No Response	0 (0%)	1 (3.45%)
Military Occupation Type		
Combat	9 (75%)	22 (75.86%)
Non-Combat	3 (25%)	7 (24.14%)

© 2024 Malarkey ME et al. *JAMA Network Open*.

Baseline ISI, mean (SD)	19.0 (4.2)	17.5 (5.3)

There were no statistically significant differences between groups.

© 2024 Malarkey ME et al. *JAMA Network Open*.

eTable 3. Baseline Clinical Scores for Participants Who Completed vs Were Missing Immediate Postintervention Follow-Up Evaluations

Baseline score	Completed immediate post-intervention follow-up evaluations	Missing immediate post-intervention follow-up evaluations	p-values
ISI	19.196 (4.67)	19.77 (3.895)	0.494
PHQ	13.179 (5.88)	14.34 (6.095)	0.322
PCL	37.719 (20.15)	41.82 (19.53)	0.291
PSQI	14.938 (2.83)	15.77 (2.42)	0.106
FACIT	81.94 (25.61)	77.59 (25.03)	0.377
Log sqrt (MIDAS)	0.615 (0.195)	0.638 (0.246)	0.598

Mean (SD). P-values based on 2-sided t-tests of differences between groups

© 2024 Malarkey ME et al. *JAMA Network Open*.

eTable 4. Baseline Clinical Scores for Participants Who Completed vs Were Missing 3-Month Post-Intervention Follow-Up Evaluation

Baseline score	Completed 3-month post-intervention follow-up evaluations	Missing 3-month post-intervention follow-up evaluations	p-values
ISI	18.73 (5.28)	19.97 (3.925)	0.153
PHQ	13.01 (7.86)	14.276 (5.63)	0.296
PCL	36.06 (22.56)	42.338 (18.66)	0.110
PSQI	14.39 (2.23)	14.98 (2.458)	0.002
FACIT	84.85 (27.26)	76.42 (23.38)	0.096
Log sqrt (MIDAS)	0.58 (0.22)	0.65 (0.22)	0.116

Mean (SD). P-values based on 2-sided t-tests of differences between groups

© 2024 Malarkey ME et al. *JAMA Network Open*.

eTable 5. Demographics for Participants Who Completed vs Were Missing Immediate Post-Intervention Follow-Up Evaluations

Characteristic, No. (%)	Completed immediate post-intervention follow-up evaluations	Missing immediate post-intervention follow-up evaluations	p-values
Age, years, mean (SD)	45.64 (9.78)	39.46 (10.35)	0.001
Female	11 (22%)	11 (19.64%)	0.765
Race			White v. Other 0.727
White	36 (72%)	42 (75%)	
Black or African American	4 (8%)	7 (12.5%)	
Asian	1 (2%)	2 (3.57%)	
Native Hawaiian or other Pacific Islander	1 (2%)	1 (1.79%)	
Multiple Races	4 (8%)	1 (1.79%)	
Other	4 (8%)	3 (5.36%)	
Ethnicity			
Hispanic or Latino	11 (22%)	11 (19.64%)	0.765
Non-Hispanic or Latino	39 (78%)	45 (80.36%)	
Educational level			0.024
High school degree or less	3 (6%)	10 (17.86%)	
Some college or college degree	21 (42%)	30 (53.57%)	
Graduate Degree	26 (52%)	16 (28.57%)	
US Geographic Region			South v. Other 0.221
West	4 (8%)	3 (5.36%)	
Midwest	1 (2%)	1 (1.79%)	
South	34 (68%)	33 (58.93%)	
Northeast	0 (0%)	2 (3.57%)	
No response	11 (22%)	17 (30.36%)	
Active Duty v. Retired			
Active Duty	37 (74%)	42 (75%)	0.091
Retired	13 (26%)	13 (23.21%)	
No Response		1 (1.79%)	
Employment Status			
Full-time	36 (72%)	42 (75%)	0.728
Part-time	2 (4%)	3 (5.36%)	
Unemployed	12 (24%)	10 (17.86%)	

© 2024 Malarkey ME et al. *JAMA Network Open*.

No Response		1 (1.79%)	
Military Branch			
Air Force	3 (6.67%)	4 (7.14%)	0.8167
Army	32 (66.67%)	33 (58.93%)	
Marine Corps	6 (13.33%)	6 (10.71%)	
Navy	8 (13.33%)	13 (23.21%)	
Other	1 (0%)	0 (0%)	
Rank			
Junior Enlisted	8 (20%)	9 (16.07%)	0.0917
NCO	15 (40%)	28 (50%)	
Officer	23 (33.33%)	14 (25%)	
Warrant Officer	1 (0%)	2 (3.57%)	
Other	2 (0%)	2 (3.57%)	
No Response	1 (0%)	1 (1.79%)	
Military Occupation Type			0.8553
Combat	34 (68%)	39 (69.64%)	
Non-Combat	16 (32%)	17 (30.36%)	

P-values based on 2-sided tests of differences between groups. Missing values removed P-values based on chi-square or Fisher's exact test (categorical variables) and t-tests (continuous variables).

© 2024 Malarkey ME et al. *JAMA Network Open*.

eTable 6. Demographics for Participants Who Completed vs Were Missing at 3-Month Post Intervention Follow-Up Evaluations

Characteristic, No., (%)	Completed 3-month post-intervention follow-up evaluations	Missing 3-month post-intervention follow-up evaluations	p-values
Age, years, mean (SD)	47.325 (8.95)	40.06 (10.50)	0.001
Female	9 (21.95%)	12 (19.05%)	0.514
Race<br>White<br>Black or African American<br>Asian<br>Native Hawaiian or other Pacific<br>Islander<br>Multiple Races<br>Other	 28 (68.29%)<br>4 (9.76%)<br>2 (4.88%)<br>0 (0%)<br><br>3 (7.32%)<br>4 (9.76%)	 49 (77.78%)<br>7 (11.11%)<br>0 (0.00%)<br>2 (3.17%)<br><br>2 (3.17%)<br>3 (4.76%)	White v. Other<br>0.281
Ethnicity<br>Hispanic or Latino<br>Non-Hispanic or Latino	 8 (19.51%)<br>33 (80.49%)	 13 (20.63%)<br>50 (79.37%)	0.889
Educational level<br>High school degree or less<br>Some college or college degree<br>Graduate Degree	 3 (7.32%)<br>13 (31.71%)<br>25 (60.98%)	 9 (14.29%)<br>36 (57.14%)<br>18 (28.57%)	0.005
US Geographic Region<br>West<br>Midwest<br>South<br>Northeast<br>No response	 3 (7.32%)<br>0 (0%)<br>30 (73.17%)<br>0 (0%)<br>8 (19.51%)	 3 (4.76%)<br>1 (1.59%)<br>38 (60.32%)<br>2 (3.17%)<br>19 (30.16%)	South v. Other<br>0.045
Active Duty v. Retired<br>Active Duty<br>Retired	 28 (68.29%)<br>13 (31.71%)	 50 (79.37%)<br>12 (19.05%)<br>1 (1.59%)	 0.238
Employment Status<br>Full-time<br>Part-time<br>Unemployed<br>No Response	 29 (70.73%)<br>3 (7.32%)<br>9 (21.95%)	 48 (76.19%)<br>2 (3.17%)<br>12 (19.05%)<br>1 (1.59%)	 0.444

© 2024 Malarkey ME et al. *JAMA Network Open*.

Military Branch			
Air Force	2 (4.88%)	6 (9.52%)	0.705
Army	27 (65.85%)	37 (58.73%)	
Marine Corps	4 (9.76%)	7 (11.11%)	
Navy	7 (17.07%)	13 (20.63%)	
Other	1 (2.44%)	0 (0%)	
Rank			
Junior Enlisted	6 (14.63%)	9 (14.29%)	0.045
NCO	10 (24.39%)	31 (49.21%)	
Officer	21 (51.22%)	18 (28.57%)	
Warrant Officer	2 (4.88%)	1 (1.59%)	
Other	1 (2.44%)	3 (4.76%)	
No Response	1 (2.44%)	1 (1.59%)	
Military Occupation Type			0.256
Combat	31 (75.61%)	41 (65.08%)	
Non-Combat	10 (24.39%)	22 (34.92%)	

P-values based on 2-sided tests of differences between groups. Missing values removed. P-values based on chi-square or Fisher’s exact test (categorical variables) and t-tests (continuous variables).

© 2024 Malarkey ME et al. *JAMA Network Open*.

eMethods. Statistical Analysis

Power Calculations: In addition to the power calculations used in the design of the study included in the protocol, we conducted subsequent power calculations using a simulation based on varying target sample sizes. We wanted to assess whether power was maintained even if target sample sizes were smaller than originally expected. These power calculations were based on: 1) original assumptions used in the original study design; 2) 3:1 treatment to control group allocation; 3) type I error rate=0.05 and 2-sided test of the parameter of interest (i.e. difference in change from baseline to post-intervention in mean ISI score between the treatment and control groups); and 4) varying levels of within-subject correlation in ISI scores. Details as they relate to the simulation are described in Fu et al. (2023); *Power Simulation Program: An Adaptable Application for Assessment of Power in Planning and Pre-Data Analysis of Clinical Study Data-An MTBI*^{2} *Study* https://zenodo.org/records/8436456.

For the simulation results as they relate to this study (see figure below), we found that for mean differences in change in mean ISI between treatment arms ≥ 3, that power of 0.8 was maintained for a range of sample sizes.

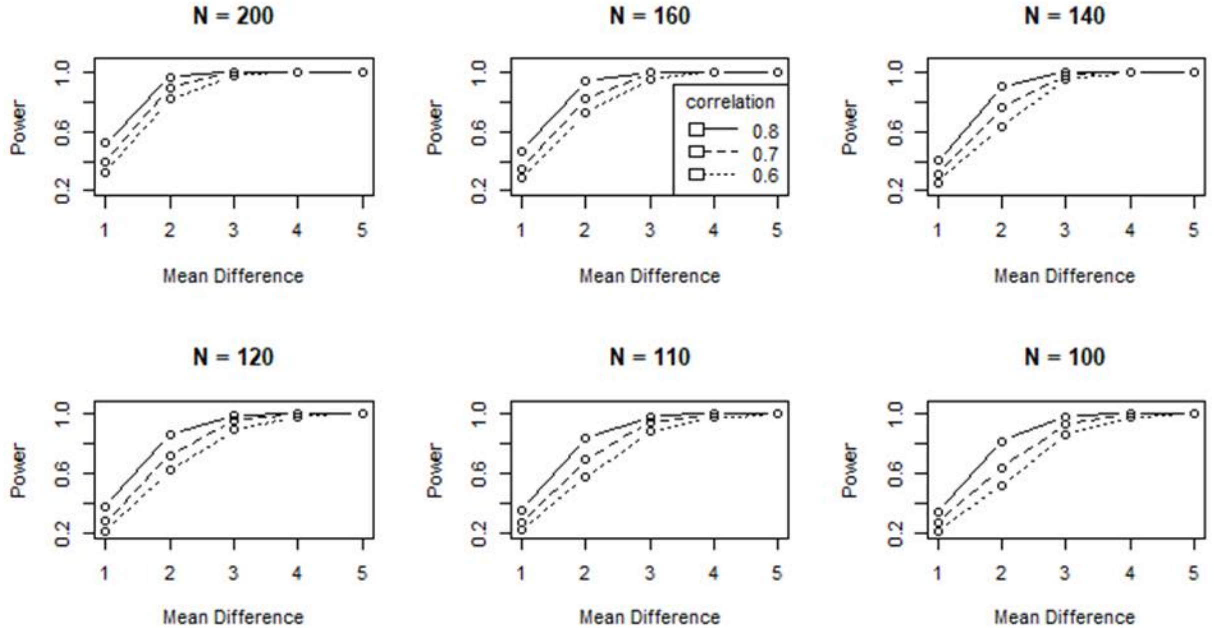

Once the SHUT-I data were collected, prior to the analysis of the study data, we conducted a power analysis given the actual data including the observed mean difference in ISI score for the parameter of interest, the observed variability in the data, and based on the actual observed underlying correlation in ISI score within subjects, using the same power calculation tool as used in the simulation. We assumed a Type I error=0.05, a two-sided test and available study N=106. This analysis indicated that the power based on the actual study data was 0.7.

© 2024 Malarkey ME et al. *JAMA Network Open*.

Given the observed differences in covariate patterns in those missing data vs. those not missing data, we conducted sensitivity analyses to address the differences in attrition rates between the groups. Specifically, we carried out sensitivity analyses as outlined in Section 11.1 of the Statistical Analysis Plan (SAP), using inverse-probability of censoring weight (IPCW) estimation. We assessed the probability of participants missing at follow-up using a pooled logistic model to predict censoring at follow-up, using participants' demographic information available at the baseline visit. We calculated the predicted probability of participants not being censored and used the reciprocal of this to assign weights to any participant with observed data at the follow-up visits, i.e., the smaller the probability of not being censored at follow-up, for example, expected for a younger participant, the greater the weight assigned to a participant with observed data at follow-up of that particular age. We assumed censoring models for different sets of baseline predictors. IPCW(1) included age only and IPCW(2) included age, military rank, and education level as different predictors of loss to follow-up. Distribution of weights are shown below for the different timepoints and across the different covariates found to be associated with missing at follow-up.

Distribution of weights (percentiles) in participants with observed data based on inverse-probability of censoring estimation (min, 25th, 50th, 75th, max).

Parameter	Original Sample*	IPCW (1)	IPCW (2)
Baseline**	NA	1,1,1,1,1	1,1,1,1,1
Follow-up Time1 (immediate)	NA	1.14, 1.23, 1.30, 1.41, 1.88	1.11, 1.20, 1.28, 1.42, 2.14
Follow-up Time2 (3-months)	NA	1.31, 1.48, 1.65, 1.75, 3.54	1.22, 1.42, 1.56, 1.83, 2.68

*No weights were applied in the original analysis of the data—i.e. each participant at each timepoint receives a weight of 1.
**For IPCW (1) and IPCW (2), given all participants in the analysis were observed at baseline, all participants received the same weight at this timepoint.

© 2024 Malarkey ME et al. *JAMA Network Open*.

Model estimates based on analysis of original study data and analyses incorporating IPCW weights

Parameter	Original analyses			
Study variables	Beta Coefficient	SE	95% CI	p-value
Intercept	18.88	1.05	(16.82, 20.94)	0.001
Treatment	0.81	1.19	(-1.52, 3.14)	0.501
Time2	-2.27	1.31	(-4.84, 0.30)	0.087
Time3	-4.48	1.49	(-7.40, -1.56)	0.003
Treatment*Time2	-3.49	1.58	(-6.59, -0.39)	0.027*
Treatment*Time3	-2.23	1.76	(-5.68, 1.22)	0.20
	IPCW (1)			
	Beta Coefficient	SE	95% CI	p-value
Intercept	18.88	1.11	(16.70, 21.06)	0.001
Treatment	0.81	1.26	(-1.66, 3.28)	0.523
Time2	-2.20	1.26	(-4.71, 0.31)	0.086
Time 3	-4.70	1.33	(-7.31, -2.09)	0.001
Treatment*Time2	-3.61	1.53	(-6.61, -0.61)	0.021*
Treatment*Time3	-2.69	1.62	(-5.87, 0.49)	0.101
	IPCW (2)			
	Beta Coefficient	SE	95% CI	p-value
Intercept	18.88	1.11	(16.70, 21.06)	0.001
Treatment	0.81	1.26	(-1.66, 3.28)	0.523
Time2	-2.24	1.28	(-4.75, 0.27)	0.084
Time 3	-4.61	1.37	(-7.30, -1.92)	0.001
Treatment*Time2	-3.54	1.55	(-6.58, -0.50)	0.025*
Treatment*Time3	-2.66	1.66	(-5.91, 0.59)	0.114

Models for loss to follow-up: IPCW(1) included age only and IPCW(2) included age, military rank, and education level as different predictors of loss to follow-up.
*Statistically significant effect in primary analysis measure.

The results of these sensitivity analyses indicate there were negligible differences in terms of inference between the original analysis and analyses that accounted for loss-to-follow based on baseline covariates that were found to differ significantly between those who were present at follow-up and those missing at follow-up.

© 2024 Malarkey ME et al. *JAMA Network Open*.

eAppendix. Additional Limitations

Additional limitations include the following:

- We have not fully analyzed the sleep diaries completed by the participants during the study. This analysis will be reported separately and is beyond the scope of the current communication.
- The intervention was designed for a desktop or laptop computer use and may not have been optimally configured for mobile phones or tablets; we do not know what devices were used by the participants.
- We have not directly assessed cognitive performance. We hypothesize that improvements in healthy sleep would correlate with improved cognitive performance but additional studies will be required to test this hypothesis.
- We do not know whether benefits of the military adapted version of SHUT-I would be similar to other digital interventions in the same domain such as "CBT-i Coach" and "Insomnia Coach" (https://mobile.va.gov/app/insomnia-coach) from the National Center for Telehealth and Technology [41 42], or SleepEZ (https://www.veterantraining.va.gov/insomnia/) from the US Department of Veterans Affairs; these interventions were not available at the time this study was designed.
- We were unable to collect a comprehensive list of reasons for participants' decision to drop out of the study; anecdotally, reasons were mainly related to scheduling issues and/or time constraints. It is not clear whether specific demographic factors or randomized group assignment affected feasibility and loss to follow up.
- We have not assessed the immediate post-intervention effects on headache/migraine disability. The MIDAS addresses a 3-month time window, and there was not enough time between baseline and the post intervention time point to allow this assessment. Future studies involving alternative measures will be required to assess for effects on headache/migraine disability in a more granular fashion.

© 2024 Malarkey ME et al. *JAMA Network Open*.

eReferences

1. Kuhn E, Miller KE, Puran D, et al. A pilot randomized controlled trial of the insomnia coach mobile app to assess its feasibility, acceptability, and potential efficacy. *Behav Ther*. 2022;53(3):440-457. Medline:35473648 doi:10.1016/j.beth.2021.11.003
2. Koffel E, Kuhn E, Petsoulis N, et al. A randomized controlled pilot study of CBT-I Coach: feasibility, acceptability, and potential impact of a mobile phone application for patients in cognitive behavioral therapy for insomnia. *Health Informatics J*. 2018;24(1):3-13. Medline:27354394 doi:10.1177/1460458216656472

© 2024 Malarkey ME et al. *JAMA Network Open*.
