# Supplementary material for: Internet-Guided Cognitive Behavioral Therapy for Insomnia Among Patients With Traumatic Brain Injury: A Randomized Clinical Trial
Source: JAMA Netw Open. 2024 Jul 9;7(7):e2420090. doi: 10.1001/jamanetworkopen.2024.20090 (PMC11234237; doi:10.1001/jamanetworkopen.2024.20090)
Supplement: Supplement 3. — Data Sharing Statement [file jamanetwopen-e2420090-s003.pdf]

Data Sharing Statement

Malarkey. Internet-Guided Cognitive Behavioral Therapy for Insomnia Among Patients With Traumatic Brain Injury. *JAMA Netw Open*. Published July 09, 2024.
doi:10.1001/jamanetworkopen.2024.20090

Data
Data available: Yes
Data types: Deidentified participant data
How to access data: request must be sent to david.brody@usuhs.edu
When available: With publication

Supporting Documents
Document types: None

Additional Information
Who can access the data: anyone requesting the data
Types of analyses: for any purpose
Mechanisms of data availability: with a signed data access agreement
